# Association between SIRT1 gene polymorphisms and susceptibility to coronary artery disease: a systematic review and meta-analysis

**DOI:** 10.3389/fcvm.2026.1850297

**Published:** 2026-07-03

**Authors:** Di An, Huan Wu, Shujin Wu, Yuanpeng Deng, Shuangshuang Yang, Wansheng Wang, Yi Xiang, Xingde Liu

**Affiliations:** 1 The Key Laboratory of Myocardial Remodeling Research, The Affiliated Hospital of Guizhou Medical University, Guiyang, Guizhou, China; 2 Guizhou University of Traditional Chinese Medicine, Guiyang, Guizhou, China; 3 The Second Affiliated Hospital of Guizhou University of Traditional Chinese Medicine, Guiyang, Guizhou, China

**Keywords:** coronary artery disease, meta-analysis, rs7069102, SIRT1 gene, systematic review

## Abstract

**Background:**

This systematic review and meta-analysis aimed to evaluate and quantitatively synthesize the available evidence on the association between SIRT1 gene polymorphisms and susceptibility to coronary artery disease (CAD).

**Methods:**

PubMed, Embase, Web of Science, and the Cochrane Library were systematically searched from inception to February 12, 2026, to identify relevant observational studies. Literature screening, data extraction, and quality assessment were independently performed by two reviewers. Meta-analyses were conducted using Stata 16.0, with odds ratios (ORs) and 95% confidence intervals (CIs) as effect measures. Subgroup and sensitivity analyses were further performed where appropriate.

**Results:**

A total of nine studies were included, with overall methodological quality ranging from moderate to high. Three SIRT1 polymorphisms, rs7069102, rs7895833, and rs4746720, were included in the quantitative synthesis. In the overall analysis, rs7069102 was not significantly associated with CAD susceptibility under any of the five genetic models; however, in the CAD subgroup, it showed a consistent risk effect across all genetic models. Rs7895833 was associated with increased CAD susceptibility only under the recessive model (OR = 1.49, 95% CI: 1.03–2.15). Rs4746720 showed significant associations under the dominant model (OR = 1.26, 95% CI: 1.02–1.55) and the heterozygote model (OR = 1.27, 95% CI: 1.01–1.58).

**Conclusion:**

Current evidence suggests that certain SIRT1 polymorphisms may be associated with CAD susceptibility, but the observed associations appear to vary by SNP locus, genetic model, and population or disease subgroup. Because the pooled estimates were derived mainly from unadjusted genotype frequencies and could not account for major cardiovascular risk factors, these findings should be interpreted cautiously. Further large, well-designed studies with appropriate adjustment for clinical confounders are needed to confirm these associations.

## 1 Introduction

Coronary artery disease (CAD) is a common cardiovascular disorder caused by coronary atherosclerosis and its related pathological processes. It encompasses multiple clinical phenotypes, including stable coronary artery stenosis, coronary heart disease (CHD), and myocardial infarction (MI), and remains one of the leading causes of mortality and disease burden worldwide (1). Although traditional risk factors, such as hypertension, dyslipidemia, smoking, and diabetes mellitus, play critical roles in the development and progression of CAD (2–4), a large body of epidemiological evidence has shown substantial interindividual differences in disease risk under similar exposure backgrounds, suggesting that genetic susceptibility is an important contributor to CAD pathogenesis (5, 6). In recent years, with the rapid advancement of genomics research, identifying key regulatory genes and their polymorphisms associated with coronary atherosclerotic disease has become an important direction for elucidating its molecular mechanisms (7, 8).

The Sirtuin 1 (SIRT1) gene encodes a nicotinamide adenine dinucleotide (NAD⁺)-dependent class III histone deacetylase and is one of the most extensively studied members of the sirtuin family (9). SIRT1 plays a central role in multiple key biological processes, including cellular energy metabolism, oxidative stress, inflammation, and cellular senescence (10). In the cardiovascular system, SIRT1 participates in maintaining vascular endothelial function, suppressing inflammatory responses, and delaying the progression of atherosclerosis through deacetylation of multiple key transcription factors and signaling molecules, such as p53, NF-*κ*B, and eNOS, thereby exerting an important regulatory role in cardiovascular homeostasis (11). Accordingly, SIRT1 is regarded as an important molecular hub linking metabolic dysregulation and vascular injury, and its potential protective role in the development and progression of CAD has attracted increasing attention (12, 13).

Genetic polymorphisms, particularly single nucleotide polymorphisms (SNPs), may alter individual susceptibility to disease by affecting gene transcription levels or protein function (14, 15). In recent years, multiple case-control studies have investigated the association between different SIRT1 SNP loci and susceptibility to CAD, including rs3758391, rs4746720, and rs7069102 (16, 17). Notably, the disease phenotypes involved in existing studies are not entirely consistent, including not only CAD and CHD but also MI, ST-segment elevation myocardial infarction (STEMI), and premature myocardial infarction (PMI). Although these phenotypes differ in clinical presentation, they all essentially belong to the spectrum of coronary atherosclerotic diseases and share a common pathophysiological basis. Therefore, integrating relevant evidence from the perspective of overall susceptibility to CAD is both reasonable and necessary. However, current findings remain inconsistent: some studies suggest that specific alleles may increase disease risk, whereas others have reported no significant association. Such inconsistency may be attributable to limited sample sizes, ethnic differences, gene-environment interactions, phenotypic heterogeneity, and differences in study design, all of which may compromise the stability and generalizability of the conclusions.

Several previous experimental and clinical studies have suggested that SIRT1 plays an important role in cardiovascular biology and atherosclerosis-related processes, including endothelial function, oxidative stress, and inflammatory regulation. Recent studies have further highlighted the potential translational significance of SIRT1 signaling in cardiovascular diseases. However, evidence regarding the association between specific SIRT1 genetic polymorphisms and susceptibility to CAD remains inconsistent, warranting a comprehensive quantitative synthesis of the available data (18–20).

Against this background, a systematic review and meta-analysis of the available evidence may improve statistical power and provide more robust effect estimates through comprehensive evidence synthesis and quantitative pooling. In addition, subgroup and sensitivity analyses may help further explore potential sources of heterogeneity and assess the robustness of the findings. Therefore, the present study aimed to systematically review and meta-analyze the association between SIRT1 gene polymorphisms and susceptibility to CAD, in order to clarify the strength and direction of this association at an overall level and to provide more reliable evidence for understanding the genetic mechanisms underlying CAD and for individualized risk assessment.

## 2 Method

This study strictly follows the Preferred Reporting Items for Systematic Reviews and Meta-Analyses (PRISMA) guidelines (21) and is registered with the PROSPERO international prospective systematic review database Registration number: CRD420261362849.

### 2.1 Search strategy

A systematic search was conducted in PubMed, Embase, Web of Science, and the Cochrane Library from database inception to February 12, 2026. The search strategy combined controlled vocabulary terms (MeSH/Emtree) with free-text terms. The main search terms included “SIRT1” OR “Sirtuin 1” OR “Sirt1”, and “CAD” OR “CHD”, among others. The search strategy was appropriately adapted according to the characteristics of each database. In addition, the reference lists of included studies and relevant reviews were manually screened to identify potentially eligible studies that might have been missed. The full search strategy is provided in Supplementary Material S1.

### 2.2 Inclusion and exclusion criteria

The inclusion criteria were as follows: (1) observational studies, including case-control studies, cross-sectional studies, or cohort studies; (2) human participants; (3) exposure of interest was SIRT1 gene polymorphisms, with at least one SIRT1 single nucleotide polymorphism (SNP) reported; (4) outcomes were CAD or its clinical phenotypes, including CHD, CAD, coronary atherosclerotic lesions, MI, STEMI, or PMI; (5) cases had a clear diagnostic basis, such as coronary angiographic evidence of coronary stenosis, clinical diagnostic criteria, electrocardiographic changes, and/or cardiac enzyme or troponin abnormalities; (6) studies provided extractable or calculable genotype/allele distribution data, or sufficient data to calculate effect estimates (ORs and 95% CIs); and (7) controls were individuals without a definite diagnosis of CAD and were reasonably comparable to the case group.

The exclusion criteria were as follows: (1) duplicate publications, reviews, systematic reviews, meta-analyses, case reports, conference abstracts, editorials, comments, animal studies, or cell experiments; (2) studies in which the population was not related to CAD, or the outcome was not directly associated with CAD; (3) studies that did not investigate SIRT1 gene polymorphisms, or only assessed SIRT1 expression, methylation, or protein levels without providing genotype/allele data for genetic association analysis; (4) studies without a control group, or with an unclear definition of controls; (5) studies from which genotype frequencies, allele frequencies, or effect estimates could not be extracted or calculated; (6) where duplicate publications from the same study population existed, only the study with the largest sample size or the most complete information was included; and (7) studies with evidently poor methodological quality and key missing data that could not be supplemented by contacting the authors.

### 2.3 Literature screening

Literature screening was independently performed by two researchers according to the predefined inclusion and exclusion criteria. First, all records retrieved from the databases were imported into EndNote X9.3.3 for reference management and duplicate removal. Next, titles and abstracts were screened to exclude clearly irrelevant studies. The full texts of the remaining articles were then retrieved and further assessed to determine final eligibility. Any disagreements arising during the screening process were resolved through discussion between the two researchers, and if necessary, adjudicated by a third researcher. The study selection process and reasons for exclusion were recorded, and a PRISMA flow diagram was generated to illustrate the screening process.

### 2.4 Data extraction

Data extraction was independently conducted by two researchers using a predesigned and pilot-tested data extraction form. The extracted information included first author, year of publication, country or region, ethnicity/race of participants, study design, disease phenotype, whether the population was a special population (e.g., patients with type 2 diabetes), sample size of cases and controls, age and sex distribution, SIRT1 polymorphism loci, genotype/allele distributions in cases and controls, and effect estimates with corresponding 95% CIs reported in the original studies, if available. When a single study reported multiple SNP loci, data for each locus were extracted separately. When multiple genetic models were reported for the same locus, raw genotype frequencies were preferentially extracted to allow for unified reanalysis. If study data were incomplete or unclearly reported, attempts were made to contact the original authors for additional information. Any discrepancies during data extraction were resolved through discussion, and if necessary, by consultation with a third researcher.

### 2.5 Risk of bias assessment

The methodological quality of the included studies was independently assessed by two researchers. For case-control and cohort studies, the Newcastle–Ottawa Scale (NOS) was used for quality assessment (22), covering three domains: selection of study groups, comparability between groups, and exposure/outcome assessment, with a total score of 9 points. Studies with NOS scores ≥7 were considered high quality, scores of 5–6 were considered moderate quality, and scores <5 were considered low quality. For cross-sectional studies, the assessment criteria recommended by the Agency for Healthcare Research and Quality (AHRQ) were applied (23). Disagreements between the two researchers were resolved through discussion, and if necessary, by consultation with a third researcher.

### 2.6 Statistical analysis

Statistical analyses were performed using Stata version 16.0. The strength of association was assessed using ORs and 95% CIs. For each SNP locus, pooled analyses were conducted under the following genetic models: allelic, homozygote, heterozygote, dominant, and recessive models. If the original studies did not directly report ORs, the corresponding ORs and 95% CIs were calculated based on genotype frequencies in cases and controls. Between-study heterogeneity was assessed using Cochran's *Q* test and the I^2^ statistic. When I^2^ < 50%, heterogeneity was considered low and a fixed-effect model was applied; otherwise, a random-effects model was used. To explore potential sources of heterogeneity, subgroup analyses were prespecified according to disease phenotype, ethnicity/region, and study population characteristics, including special populations (e.g., patients with type 2 diabetes) vs. general populations. For analyses including at least five studies, sensitivity analyses were performed using a leave-one-out approach to assess the robustness of the pooled results. For analyses including at least ten studies, publication bias was evaluated using Egger's test and visually inspected with funnel plots; when fewer than ten studies were included, formal tests for publication bias were not performed. All statistical tests were two-sided, and *P* < 0.05 was considered statistically significant. Hardy-Weinberg equilibrium (HWE) in the control groups was assessed for each SNP using genotype distributions reported in the original studies. An HWE *P* value < 0.05 was considered indicative of deviation from equilibrium. Where studies deviated from HWE, additional sensitivity analyses were performed by excluding these studies to evaluate the robustness of the pooled estimates.

## 3 Results

### 3.1 Literature search and study selection

**Figure 1:**
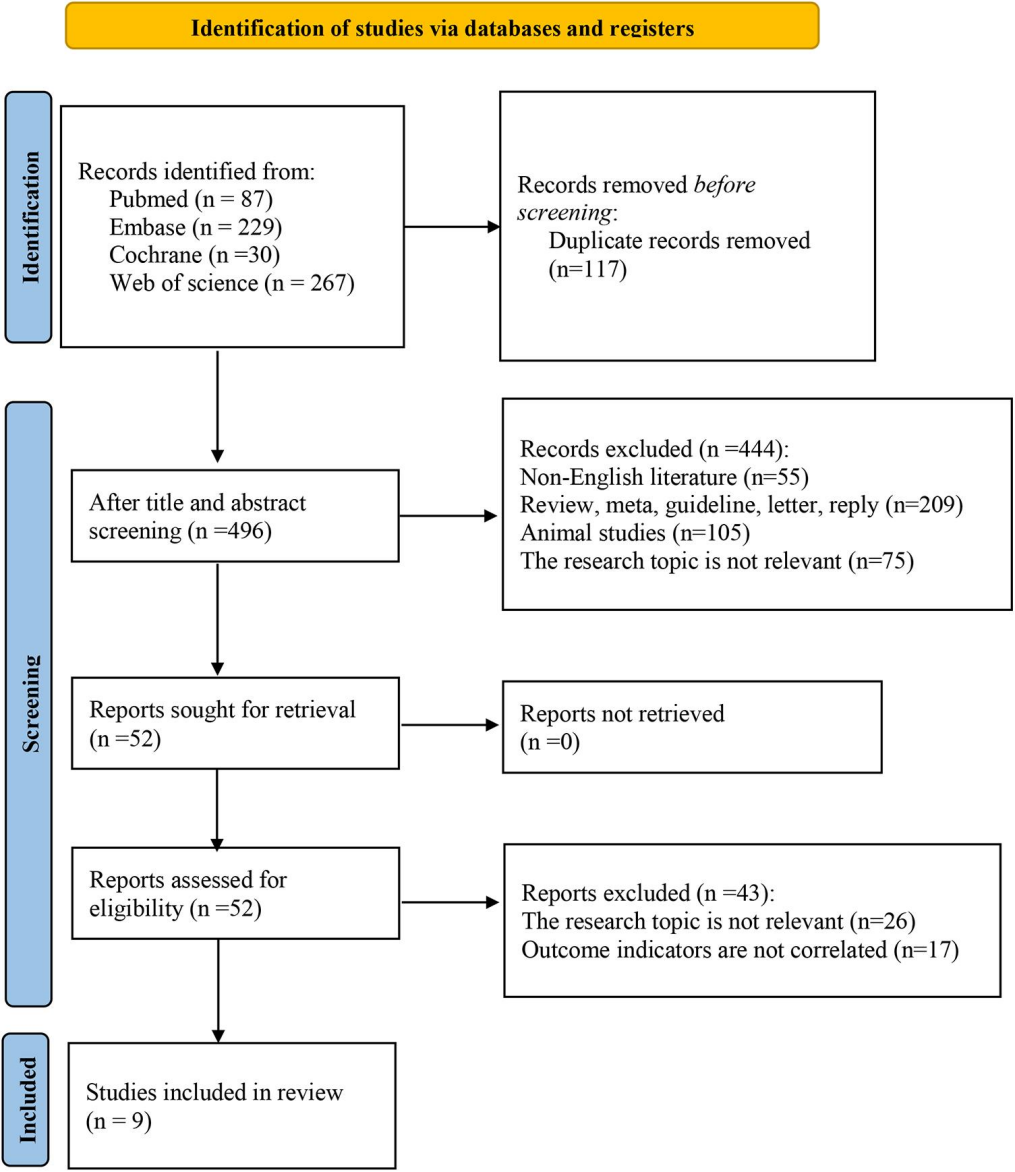
Prisma literature screening flowchart.

A total of 613 potentially relevant records were identified through database searching. After removal of 117 duplicates, 496 records remained for title and abstract screening, of which 444 were excluded. The full texts of 52 articles were then assessed for eligibility, and 43 were further excluded. Ultimately, 9 studies were included in the systematic review and meta-analysis (16, 24–31). The literature screening process is shown in Figure 1.

### 3.2 Characteristics of the included studies

The 9 included studies were published between 2014 and 2024 and involved populations from five countries: China, Iran, Turkey, South Africa, and Egypt. Among them, 7 studies were conducted in Asian populations, and 2 studies were conducted in African populations. In terms of study design, 8 were case-control studies, and 1 was a cross-sectional study. The disease phenotypes mainly included CAD and MI. Most studies were conducted in general populations (16, 24, 25, 28–31), while only 2 studies involved special populations (26, 27). Sample sizes varied substantially across studies, ranging from 50 to 509 in the case groups and from 50 to 654 in the control groups. The proportion of male participants was generally high, with males accounting for more than 75% in several studies; in one study, both the case and control groups consisted entirely of male participants (29). The SIRT1 polymorphisms ultimately included in the quantitative synthesis were rs4746720, rs7069102, and rs7895833. Detailed characteristics of the included studies are presented in Table 1.

**Table 1:** Basic characteristics of the included studies.

Study	Country	Ethnicity	Study design	Disease phenotype	Special population	Samplesize (cases/controls)	Age (cases/controls) (mean ± SD)	Male % (cases/controls)	SIRT1 SNPs	Control genotype counts	HWE *P* value
Song et al. (16)	China	Asian	Case–control	CAD	General population	509/552	56.99 ± 10.75/56.14 ± 11.40	47.2%/46.9%	**rs4746720,**	**CC/CT/TT** **=** **129/246/177**	**0**.**016**
									rs7895833	GG/GA/AA = 292/225/35	0.385
Nasiri et al. (24)	Iran	Asian	Case–control	CAD	General population	150/150	62.09 ± 10.31/62.18 ± 10.50	50.7%/51.3%	**rs4746720**	**TT/TC/CC** **=** **97/53/0**	**0**.**005**
Cheng et al. (25)	China	Asian	Case–control	MI	General population	287/654	61.67 ± 11.95/61.37 ± 12.34	77.7%/58.3%	rs4746720	CC/CT/TT = 119/326/209	0.750
									rs7069102	CC/CG/GG = 486/156/12	1.000
Wang et al. (26)	China	Asian	Case–control	CAD	T2D patients	297/195	64.67 ± 10.37/62.33 ± 11.11	63.3%/40.0%	rs4746720	TT/TC/CC = 54/110/31	0.059
Heidari et al. (27)	Iran	Asian	Case–control	CAD	T2D patients	50/50	63.56 ± 8.77/60.36 ± 8.15	54.0%/54.0%	rs7069102	CC/CG/GG = 33/14/3	0.380
Kilic et al. (28)	Turkey	Asian	Case–control	CAD	General population	278/135	58.99 ± 10.56/48.52 ± 5.87	78.1%/88.9%	rs7895833	AA/AG/GG = 98/36/1	0.470
									rs7069102	CC/CG/GG = 23/67/45	0.862
Ramkaran et al. (29)	South Africa	African	Case–control	CAD	General population	204/198	NR	100%/100%	rs7895833	AA/AG/GG = 34/48/17	1.000
Yamac et al. (30)	Turkey	Asian	Case–control	MI	General population	108/91	40.74 ± 3.82/32.7 ± 6.3	87.0%/57.1%	**rs7895833**	**AA/AG/GG** **=** **52/39/0**	**0**.**010**
									rs7069102	CC/CG/GG = 10/46/35	0.497
Altaher et al. (31)	Egypt	African	Cross-sectional	MI	General population	140/140	37.4 ± 2.9/37.4 ± 2.7	77.9%/75.7%	**rs7069102**	**CC/CG/GG** **=** **7/72/61**	**0**.**017**

SIRT1,  sirtuin 1; SNPs, single nucleotide polymorphisms; CAD, coronary artery disease; MI, myocardial infarction; T2D, type 2 diabetes; ECG, electrocardiogram; NR, not reported; SD, standard deviation; HWE, Hardy-Weinberg equilibrium.

*P* < 0.05 was considered statistically significant.

### 3.3 Quality assessment

Methodological quality assessment showed that the 8 case-control studies (16, 24–30) had NOS scores ranging from 6 to 8, indicating overall moderate to high quality. Cheng et al. (25), Heidari et al. (2023), Kilic et al. (28), and Ramkaran et al. (29) received relatively high scores, suggesting that these studies were generally well conducted in terms of case definition, control selection, exposure assessment, and group comparability. Song et al. (16), Nasiri et al. (24), Wang et al. (26), and Yamac et al. (30) were rated as moderate quality, with main limitations including insufficient representativeness of controls, limited adjustment for confounding factors, or lack of information on response rates. In addition, one cross-sectional study (31) was assessed using the AHRQ criteria and was rated as moderate quality overall. Taken together, the methodological quality of the included studies was considered acceptable. Detailed quality assessment results are provided in Supplementary Material S2.

### 3.4 Meta-analysis

#### 3.4.1 Association between rs7069102 polymorphism and susceptibility to CAD

A total of 5 studies (25, 27, 28, 30, 31) were included in the quantitative synthesis of the SIRT1 rs7069102 locus. In the overall analysis, no significant association was observed between the rs7069102 polymorphism and susceptibility to CAD under the allelic model (G vs. C) (OR = 1.16, 95% CI: 0.61–2.21), with substantial between-study heterogeneity (I^2^ = 93.7%). Likewise, under the dominant model (GG + CG vs. CC), recessive model (GG vs. CG + CC), homozygote model (GG vs. CC), and heterozygote model (CG vs. CC), the pooled results were all statistically non-significant, with ORs of 1.24 (95% CI: 0.64–2.42, I^2^ = 81.0%), 1.10 (95% CI: 0.34–3.50, I^2^ = 93.4%), 1.17 (95% CI: 0.29–4.76, I^2^ = 91.1%), and 1.24 (95% CI: 0.80–1.93, I^2^ = 52.0%), respectively. These findings suggest that no stable significant association was observed between the rs7069102 locus and CAD risk in the overall population, although moderate to high heterogeneity was present in most genetic models. Detailed results are presented in Table 2, and sensitivity analysis results are shown in Supplementary Material S3; no single study was found to substantially influence the pooled estimates.

**Table 2:** Meta-analysis results of the association between SIRT1 rs7069102 polymorphism and coronary artery disease susceptibility.

SIRT1 SNPs	Gene model	Subgroup	Subgroup type	No.	OR (95% CI)	I-square	*P* value	Model
rs7069102 (Exp = G)	Allelic model (G vs. C)	Total	/	5	1.16 (0.61, 2.21)	93.70%	0.648	Random
		Population	General	4	1.02 (0.49, 2.10)	95.00%	0.968	Random
			Special	1	2.15 (1.14, 4.08)	/	0.019	Random
		Disease	MI	3	0.76 (0.37, 1.57)	93.00%	0.457	Random
			CAD	2	2.37 (1.79, 3.14)	0.00%	<0.001	Fixed
		Ethnicity	Asian	4	1.48 (0.87, 2.54)	88.10%	0.151	Random
			African	1	0.44 (0.31, 0.63)	/	<0.001	Random
		Study type	Case-control	4	1.48 (0.87, 2.54)	88.10%	0.151	Random
			Cross-sectional	1	0.44 (0.31, 0.63)	/	<0.001	Random
	Dominant model (GG + CG vs. CC)	Total	/	5	1.24 (0.64, 2.42)	81.00%	0.529	Random
		Population	General	4	1.07 (0.47, 2.39)	84.60%	0.877	Random
			Special	1	2.28 (1.02, 5.11)	/	0.046	Random
		Disease	MI	3	0.74 (0.28, 2.01)	84.50%	0.56	Random
			CAD	2	2.66 (1.60, 4.43)	0.00%	<0.001	Fixed
		Ethnicity	Asian	4	1.72 (1.10, 2.69)	52.50%	0.018	Random
			African	1	0.27 (0.11, 0.65)	/	0.003	Random
		Study type	Case-control	4	1.72 (1.10, 2.69)	52.50%	0.018	Random
			Cross-sectional	1	0.27 (0.11, 0.65)	/	0.003	Random
	Recessive model (GG vs. CG + CC)	Total	/	5	1.10 (0.34, 3.50)	93.40%	0.873	Random
		Population	General	4	0.90 (0.24, 3.30)	94.90%	0.872	Random
			Special	1	2.98 (0.74, 11.99)	/	0.123	Random
		Disease	MI	3	0.57 (0.20, 1.66)	86.90%	0.303	Random
			CAD	2	3.09 (2.05, 4.67)	0.00%	<0.001	Fixed
		Ethnicity	Asian	4	1.61 (0.55, 4.73)	88.70%	0.387	Random
			African	1	0.25 (0.15, 0.44)	/	<0.001	Random
		Study type	Case-control	4	1.61 (0.55, 4.73)	88.70%	0.387	Random
			Cross-sectional	1	0.25 (0.15, 0.44)	/	<0.001	Random
	Homozygote model (GG vs. CC)	Total	/	5	1.17 (0.29, 4.76)	91.10%	0.829	Random
		Population	General	4	0.90 (0.18, 4.57)	92.90%	0.895	Random
			Special	1	3.83 (0.92, 15.98)	/	0.066	Random
		Disease	MI	3	0.50 (0.09, 2.71)	89.80%	0.424	Random
			CAD	2	4.56 (2.42, 8.58)	0.00%	<0.001	Fixed
		Ethnicity	Asian	4	2.09 (0.75, 5.82)	78.60%	0.157	Random
			African	1	0.11 (0.04, 0.30)	/	<0.001	Random
		Study type	Case-control	4	2.09 (0.75, 5.82)	78.60%	0.157	Random
			Cross-sectional	1	0.11 (0.04, 0.30)	/	<0.001	Random
	Heterozygote model (CG vs. CC)	Total	/	5	1.24 (0.80, 1.93)	52.00%	0.343	Random
		Population	General	4	1.12 (0.67, 1.89)	59.90%	0.66	Random
			Special	1	1.95 (0.81, 4.66)	/	0.134	Random
		Disease	MI	3	0.94 (0.45, 1.94)	69.60%	0.867	Random
			CAD	2	1.81 (1.06, 3.12)	0.00%	0.031	Fixed
		Ethnicity	Asian	4	1.45 (1.12, 1.88)	0.00%	0.005	Fixed
			African	1	0.40 (0.16, 0.98)	/	0.044	Random
		Study type	Case-control	4	1.45 (1.12, 1.88)	0.00%	0.005	Fixed
			Cross-sectional	1	0.40 (0.16, 0.98)	/	0.044	Random

SNPs, single nucleotide polymorphisms; OR, odds ratio; CI, confidence interval; Exp, exposure allele; No., number of included studies; CAD, coronary artery disease; MI, myocardial infarction.

Subgroup analysis revealed marked differences across disease phenotypes. In the CAD subgroup (27, 28), the rs7069102 locus was significantly associated with increased disease risk under all five genetic models, with low or no heterogeneity: allelic model, OR = 2.37 (95% CI: 1.79–3.14, I^2^ = 0.0%); dominant model, OR = 2.66 (95% CI: 1.60–4.43, I^2^ = 0.0%); recessive model, OR = 3.09 (95% CI: 2.05–4.67, I^2^ = 0.0%); homozygote model, OR = 4.56 (95% CI: 2.42, 8.58, I^2^ = 0.0%); and heterozygote model, OR = 1.81 (95% CI: 1.06, 3.12, I^2^ = 0.0%). In contrast, no statistically significant association was observed in the MI subgroup under either the allelic model or the other genetic models, and heterogeneity remained high. These findings suggest that differences in disease phenotype may be an important source of heterogeneity in the overall analysis.

After stratification by population characteristics, no significant association was observed under any of the five genetic models in the general population subgroup. In the special population subgroup, however, significant associations were observed only under the allelic model (OR = 2.15, 95% CI: 1.14–4.08) and the dominant model (OR = 2.28, 95% CI: 1.02–5.11). As this subgroup included only one study, the results should be interpreted with caution.

After stratification by ethnicity, in the Asian population subgroup, the dominant model (OR = 1.72, 95% CI: 1.10–2.69) and the heterozygote model (OR = 1.45, 95% CI: 1.12–1.88) suggested that the rs7069102 locus might be associated with an increased risk of CAD, whereas the allelic, recessive, and homozygote models did not reach statistical significance. The African subgroup included only one study, which suggested a protective association of the G allele under all genetic models; however, given the limited evidence, no robust conclusion can be drawn.

After stratification by study design, the results of the case-control studies were generally consistent with those of the Asian subgroup, whereas the single cross-sectional study showed an effect direction different from that observed in the case-control studies. Overall, although the rs7069102 locus was not significantly associated with susceptibility to CAD in the overall analysis, it showed a relatively consistent and stable risk effect in the CAD subgroup, suggesting that its association may be influenced by disease phenotype, population characteristics, and study design.

#### 3.4.2 Association between rs7895833 polymorphism and susceptibility to CAD

A total of 4 studies (16, 28, 29, 31) were included in the quantitative synthesis of the SIRT1 rs7895833 locus. In the overall analysis, no statistically significant association was observed between the rs7895833 polymorphism and susceptibility to CAD under the allelic model (G vs. A) (OR = 1.08, 95% CI: 0.93–1.26, *P* = 0.290), and no substantial heterogeneity was detected (I^2^ = 0.0%). Similarly, under the dominant model (GG + AG vs. AA), homozygote model (GG vs. AA), and heterozygote model (AG vs. AA), the pooled results were also non-significant, with ORs of 1.02 (95% CI: 0.85–1.23, *P* = 0.822), 1.45 (95% CI: 0.99–2.14, *P* = 0.059), and 0.96 (95% CI: 0.78–1.16, *P* = 0.651), respectively. A statistically significant association was observed only under the recessive model (GG vs. AG + AA) (OR = 1.49, 95% CI: 1.03–2.15, *P* = 0.035), with no obvious heterogeneity (I^2^ = 0.0%). Detailed results are shown in Table 3 and Supplementary Material S4.

**Table 3:** Meta-analysis results of the association between SIRT1 rs7895833 polymorphism and coronary artery disease susceptibility.

SIRT1 SNPs	Gene model	Subgroup	Subgroup type	No.	OR (95% CI)	I-square	*P* value	Model
rs7895833 (Exp = G)	Allelic model (G vs. A)	Total	/	4	1.08 (0.93, 1.26)	0.00%	0.29	Fixed
		Disease	MI	1	1.21 (0.75, 1.93)	/	0.433	Fixed
			CAD	3	1.07 (0.91, 1.26)	0.00%	0.395	Fixed
		Ethnicity	Asian	3	1.11 (0.94, 1.30)	0.00%	0.224	Fixed
			African	1	0.96 (0.65, 1.44)	/	0.853	Fixed
	Dominant model (GG + AG vs. AA)	Total	/	4	1.02 (0.85, 1.23)	0.00%	0.822	Fixed
		Disease	MI	1	1.31 (0.75, 2.30)	/	0.348	Fixed
			CAD	3	0.99 (0.81, 1.21)	0.00%	0.923	Fixed
		Ethnicity	Asian	3	1.03 (0.85, 1.26)	0.00%	0.748	Fixed
			African	1	0.93 (0.52, 1.66)	/	0.807	Fixed
	Recessive model (GG vs. AG + AA)	Total	/	4	1.49 (1.03, 2.15)	0.00%	0.035	Fixed
		Disease	MI	1	0.85 (0.05, 13.81)	/	0.91	Fixed
			CAD	3	1.50 (1.03, 2.18)	0.00%	0.032	Fixed
		Ethnicity	Asian	3	1.71 (1.11, 2.62)	0.00%	0.015	Fixed
			African	1	0.99 (0.47, 2.07)	/	0.974	Fixed
	Homozygote model (GG vs. AA)	Total	/	4	1.45 (0.99, 2.14)	0.00%	0.059	Fixed
		Disease	MI	1	0.96 (0.06, 15.80)	/	0.979	Fixed
			CAD	3	1.46 (0.99, 2.16)	0.00%	0.056	Fixed
		Ethnicity	Asian	3	1.64 (1.06, 2.55)	0.00%	0.028	Fixed
			African	1	0.94 (0.42, 2.14)	/	0.891	Fixed
	Heterozygote model (AG vs. AA)	Total	/	4	0.96 (0.78, 1.16)	0.00%	0.651	Fixed
		Disease	MI	1	1.31 (0.75, 2.30)	/	0.348	Fixed
			CAD	3	0.91 (0.74, 1.13)	0.00%	0.403	Fixed
		Ethnicity	Asian	3	0.96 (0.78, 1.18)	0.00%	0.694	Fixed
			African	1	0.92 (0.50, 1.71)	/	0.804	Fixed

SNPs, single nucleotide polymorphisms; OR, odds ratio; CI, confidence interval; Exp, exposure allele; No., number of included studies; CAD, coronary artery disease; MI, myocardial infarction.

After stratification by disease phenotype, a statistically significant association remained under the recessive model in the CAD subgroup (OR = 1.50, 95% CI: 1.03–2.18, *P* = 0.032), whereas the homozygote model showed only borderline significance (OR = 1.46, 95% CI: 0.99–2.16, *P* = 0.056). No significant association was observed in the remaining models. Because only one study was included in the MI subgroup, no statistically significant association was observed under any genetic model.

After stratification by ethnicity, both the recessive model (OR = 1.71, 95% CI: 1.11–2.62, *P* = 0.015) and the homozygote model (OR = 1.64, 95% CI: 1.06–2.55, *P* = 0.028) showed statistically significant associations in the Asian population subgroup, whereas the allelic, dominant, and heterozygote models did not. Only one study was included in the African subgroup, and no significant association was observed under any genetic model.

#### 3.4.3 Association between rs4746720 polymorphism and susceptibility to CAD

A total of 4 studies (16, 24–26) were included in the quantitative synthesis of the SIRT1 rs4746720 locus. In the overall analysis, no statistically significant association was observed between the rs4746720 polymorphism and susceptibility to CAD under the allelic model (T vs. C) (OR = 1.07, 95% CI: 0.96–1.20, *P* = 0.227), and no substantial heterogeneity was detected (I^2^ = 0.0%). A statistically significant association was observed under the dominant model (TT + CT vs. CC) (OR = 1.26, 95% CI: 1.02–1.55, *P* = 0.033). Under the recessive model (TT vs. CT + CC) and the homozygote model (TT vs. CC), the pooled results were not statistically significant, with ORs of 1.01 (95% CI: 0.86–1.19, *P* = 0.918) and 1.24 (95% CI: 0.98–1.58, *P* = 0.074), respectively. A statistically significant association was also observed under the heterozygote model (CT vs. CC) (OR = 1.27, 95% CI: 1.01–1.58, *P* = 0.038). Heterogeneity was low across all genetic models (I^2^ = 0.0%). Detailed results are shown in Table 4 and Supplementary Material S5.

**Table 4:** Meta-analysis results of the association between SIRT1 rs4746720 polymorphism and coronary artery disease susceptibility.

SIRT1 SNPs	Gene model	Subgroup	Subgroup type	No.	OR (95% CI)	I-square	*P* value	Model
rs4746720(Exp = T)	Allelic model (T vs. C)	Total	/	4	1.07 (0.96, 1.20)	0.00%	0.227	Fixed
		Population	General population	3	1.07 (0.95, 1.21)	22.50%	0.287	Fixed
			Special population	1	1.08 (0.83, 1.40)	/	0.567	Fixed
		Disease	MI	1	1.04 (0.85, 1.27)	/	0.699	Fixed
			CAD	3	1.09 (0.95, 1.24)	0.00%	0.231	Fixed
	Dominant model (TT + CT vs. CC)	Total	/	4	**1.26** (**1.02, 1.55)**	0.00%	0.033	Fixed
		Population	General population	3	**1.29** (**1.02, 1.63)**	0.00%	0.032	Fixed
			Special population	1	1.12 (0.68, 1.84)	/	0.667	Fixed
		Disease	MI	1	1.17 (0.80, 1.69)	/	0.421	Fixed
			CAD	3	**1.30** (**1.01, 1.68)**	0.00%	0.041	Fixed
	Recessive model (TT vs. CT + CC)	Total	/	4	1.01 (0.86, 1.19)	0.00%	0.918	Fixed
		Population	General population	3	0.99 (0.83, 1.18)	0.00%	0.895	Fixed
			Special population	1	1.12 (0,75, 1.67)	/	0.587	Fixed
		Disease	MI	1	0.99 (0.73, 1.33)	/	0.94	Fixed
			CAD	3	1.02 (0.84, 1.24)	0.00%	0.863	Fixed
	Homozygote model (TT vs. CC)	Total	/	4	1.24 (0.98, 1.58)	0.00%	0.074	Fixed
		Population	General population	3	1.26 (0.96, 1.63)	0.00%	0.091	Fixed
			Special population	1	1.19 (0.67, 2.11)	/	0.555	Fixed
		Disease	MI	1	1.13 (0.74, 1.71)	/	0.579	Fixed
			CAD	3	1.30 (0.98, 1.74)	0.00%	0.073	Fixed
	Heterozygote model (CT vs. CC)	Total	/	4	**1.27** (**1.01, 1.58)**	0.00%	0.038	Fixed
		Population	General population	3	**1.31** (**1.02, 1.68)**	0.00%	0.032	Fixed
			Special population	1	1.08 (0.64, 1.82)	/	0.768	Fixed
		Disease	MI	1	1.19 (0.80, 1.76)	/	0.383	Fixed
			CAD	3	1.31 (1.00, 1.71)	0.00%	0.054	Fixed

SNPs, single nucleotide polymorphisms; OR, odds ratio; CI, confidence interval; Exp, exposure allele; No., number of included studies; CAD, coronary artery disease; MI, myocardial infarction.

*P* < 0.05 was considered statistically significant.

After stratification by population type, statistically significant associations were observed in the general population subgroup under the dominant model (OR = 1.29, 95% CI: 1.02–1.63, *P* = 0.032) and the heterozygote model (OR = 1.31, 95% CI: 1.02–1.68, *P* = 0.032), whereas the allelic, recessive, and homozygote models remained non-significant. Only one study was included in the special population subgroup, and no statistically significant association was observed under any genetic model.

After stratification by disease phenotype, a statistically significant association was observed under the dominant model in the CAD subgroup (OR = 1.30, 95% CI: 1.01–1.68, *P* = 0.041), whereas no significant association was found under the allelic, recessive, homozygote, or heterozygote models. Because only one study was included in the MI subgroup, no statistically significant association was observed under any genetic model.

### 3.5 Sensitivity analysis based on Hardy–Weinberg equilibrium

To evaluate the robustness of the pooled estimates, additional sensitivity analyses were performed after excluding studies in which the control populations deviated from HWE (Supplementary Material S6). For rs7069102, after excluding the study by Altaher et al. (31), significant associations were observed under the dominant model (OR = 1.72, 95% CI: 1.10–2.69, *P* = 0.018) and the heterozygote model (OR = 1.45, 95% CI: 1.12–1.88, *P* = 0.005), whereas the allelic, recessive, and homozygote models remained non-significant. For rs7895833, after excluding the study by Yamac et al. (30), the previously significant association under the recessive model was no longer observed (OR = 1.02, 95% CI: 0.81–1.28, *P* = 0.887). For rs4746720, after excluding the studies by Song et al. (16) and Nasiri et al. (24), the associations under the dominant and heterozygote models were no longer statistically significant. Overall, these findings indicate that the pooled estimates for rs7895833 and rs4746720 were sensitive to the inclusion of studies deviating from HWE, whereas the association for rs7069102 varied after exclusion of the HWE-violating study. Therefore, these results should be interpreted with caution.

## 4 Discussion

This study systematically reviewed and quantitatively synthesized the available evidence on the association between SIRT1 gene polymorphisms and susceptibility to CAD. The results showed that the associations between different SIRT1 polymorphic loci and susceptibility to CAD were not consistent, and varied substantially across different genetic models, disease phenotypes, and population subgroups. In the overall analysis, rs7069102 did not show a stable significant association with susceptibility to CAD; however, in the CAD subgroup, this locus exhibited directionally consistent and statistically significant risk effects across all five genetic models, accompanied by a marked reduction in heterogeneity. Rs7895833 showed a significant association only under the recessive model in the overall analysis, suggesting that its effect may primarily exist in individuals with homozygous variation. In contrast, rs4746720 showed statistically significant associations with increased susceptibility to CAD under the dominant and heterozygote models, with overall low heterogeneity. These findings suggest that the relationship between SIRT1 polymorphisms and CAD is not a single, stable main effect, but rather a complex genetic association characterized by locus specificity, phenotype dependence, and population background dependence.

One of the most notable findings of this study was the inconsistency between the overall analysis and the CAD subgroup analysis for rs7069102. In the overall analysis, no significant association was observed under any genetic model, and most models showed high heterogeneity. However, in the CAD subgroup, this locus showed relatively consistent risk effects under the allelic, dominant, recessive, homozygote, and heterozygote models, with heterogeneity almost completely eliminated. This phenomenon suggests that differences in disease phenotype may be an important source of instability in the overall results. Although CAD, CHD, MI, STEMI, and PMI can all be classified within the spectrum of coronary atherosclerotic diseases, the pathological stages and clinical implications they represent are not entirely the same. CAD more often reflects the burden of coronary atherosclerosis and the formation of chronic stenosis (32), whereas MI, especially STEMI or premature MI, more strongly reflects terminal pathological processes such as plaque instability, local inflammatory activation, thrombosis, and acute ischemic events (33, 34). Therefore, if a given SIRT1 locus is mainly involved in relatively early atherosclerotic processes, such as endothelial injury, maintenance of chronic inflammation, or lipid deposition, it may be more likely to show a stable association in the CAD phenotype, whereas in MI-related phenotypes, which are more strongly influenced by acute triggering factors, such an observed genetic association may be diluted or obscured by acute disease-specific factors. In other words, although combining different clinical phenotypes into a unified outcome may improve statistical power, it may also introduce genuine clinical heterogeneity, thereby weakening the association signal of specific loci at particular disease stages. It should be noted that the CAD subgroup for rs7069102 was based on only two studies, one of which involved a special population rather than a general population. Therefore, the observed association may reflect not only differences in disease phenotype but also differences in underlying population characteristics. Because disease phenotype and population background are not completely independent in the available evidence, their potential interaction cannot be excluded. Consequently, the apparent subgroup effect should be interpreted cautiously until additional studies become available.

Compared with rs7069102, rs7895833 and rs4746720 displayed different genetic model characteristics. In the overall analysis, rs7895833 was statistically significant only under the recessive model, whereas no significant association was observed under the allelic, dominant, or heterozygote models. This suggests that the potential effect of this locus may depend more on the homozygous variant state, meaning that only when two risk alleles are simultaneously present is the effect sufficient to produce a detectable impact on disease susceptibility. This pattern usually implies that its genetic effect may have a threshold characteristic, or that the locus itself may not be the true functional variant but rather reflects underlying risk more clearly in specific genetic models through linkage disequilibrium with a functional variant (35, 36). By contrast, rs4746720 showed significant associations under the dominant and heterozygote models, whereas the recessive model did not reach statistical significance, suggesting that the effect of this locus may be manifested by the presence of a single variant allele and may not depend on homozygosity. These differences may suggest that different SIRT1 loci could be associated with CAD susceptibility through different biological pathways, but may instead operate through distinct layers of gene regulation, such as transcriptional activity, RNA stability, splicing regulation, or interactions with adjacent regulatory elements (37).

From a mechanistic perspective, the locus-specific differences observed in this study are biologically plausible. The role of SIRT1 in the development and progression of CAD may be more closely related to the regulation of chronic vascular wall injury rather than to the direct determination of acute cardiovascular events (38, 39). The formation and progression of coronary atherosclerosis involve multiple processes, including endothelial dysfunction, enhanced oxidative stress, persistent activation of inflammatory cascades, macrophage lipid uptake, vascular smooth muscle cell migration and proliferation, and cellular senescence (40), all of which are closely linked to SIRT1. If certain polymorphic loci are associated with reduced SIRT1 expression or activity, as suggested by previous experimental studies, they may potentially weaken its inhibitory effects on inflammatory pathways and oxidative stress responses, thereby potentially contributing to persistent endothelial injury, unresolved vascular inflammation, and plaque formation or progression, unresolved chronic vascular inflammation, and further promotion of plaque formation and progression (41). In addition, it has been proposed that reduced SIRT1 function may affect vasodilatory responses, lipid homeostasis, and cell survival regulation, potentially increasing susceptibility to developing an atherosclerosis-susceptible background (38, 39). Within this framework, SIRT1-related variants may be more likely to influence the baseline risk of “whether coronary lesions are more likely to develop,” whereas their explanatory power for “whether these lesions further progress to acute infarction” may be relatively limited, as the latter is also influenced by coagulation status, plaque rupture tendency, amplification of acute inflammation, and external triggering factors. This may partly explain why some loci showed more stable effects in the CAD subgroup than in the MI subgroup.

The subgroup differences observed across ethnic groups and study population characteristics further suggest that genetic background and metabolic environment may modulate the phenotypic expression of SIRT1 polymorphism effects. In the ethnicity-stratified analyses, some loci showed clearer risk associations in Asian populations, whereas the results in African populations were less stable and even showed different effect directions for certain loci. At least two explanations may account for this discrepancy. First, allele frequencies and linkage disequilibrium structures differ across ethnic groups (42). The same SNP locus may be closely linked to a true functional variant in one population but not in another, thereby resulting in differences in the direction and magnitude of the observed risk effect. Second, the number of studies involving African populations was limited, and the sample sizes were relatively small, leading to unstable estimates and making it difficult to draw reliable conclusions. Therefore, the current findings regarding ethnic differences should be regarded as suggestive rather than definitive. At the same time, differences between special-population subgroups and general-population subgroups also suggest that the baseline metabolic state may influence the manifestation of SIRT1 genetic effects. For example, in individuals with type 2 diabetes or abnormal glucose metabolism, the body is already under stronger oxidative stress, chronic low-grade inflammation, and endothelial dysfunction (43). Under such conditions, the marginal effects of SIRT1-related variants may be amplified, making significant associations easier to detect. However, because the special-population subgroup included only a limited number of studies, most of which were based on single studies, these findings should still be interpreted with caution. Therefore, the current evidence primarily reflects the genetic characteristics of Asian and African populations and should not be directly extrapolated to other ethnic groups without further validation.

The sources of heterogeneity in this study likely reflect more than differences in sample size alone; they may also indicate real differences at multiple levels. First, there were differences in disease definitions and outcome phenotypes. Although it is reasonable to integrate CAD, CHD, and MI-related phenotypes from the perspective of the coronary atherosclerotic disease spectrum, differences in diagnostic criteria, outcome definitions, and case inclusion across studies may nevertheless introduce clinical heterogeneity. Second, differences in population composition, including age distribution, sex ratio, comorbid diabetes, and control source, may also affect the expression of genetic effects. Third, differences in study design should not be overlooked. Case-control and cross-sectional studies differ inherently in sample selection, bias control, temporal interpretation of exposure and outcome, and adjustment for confounders, which may lead to inconsistencies in effect direction and magnitude. In particular, the present meta-analysis included one cross-sectional study together with multiple case-control studies. Although both study designs can provide comparable odds ratio estimates for genetic association analyses and therefore were considered eligible for pooling, they differ in participant selection, temporal interpretation, and susceptibility to selection and prevalence-incidence biases. Notably, the cross-sectional study showed an effect direction different from that observed in the case-control studies for rs7069102, suggesting that study design itself may contribute to heterogeneity. Because only one cross-sectional study was available, it was not possible to draw definitive conclusions regarding design-specific effects, and the pooled findings should therefore be interpreted with caution. Finally, only a small number of studies were included for some loci, and certain subgroup analyses were based on only one study, making the pooled results more vulnerable to the influence of individual study weights. Therefore, the phenomenon observed in this study, in which the overall analysis was non-significant whereas subgroup analyses were significant, does not necessarily indicate that the conclusions are unreliable. Rather, it may suggest that genuine locus-specific effects were averaged out or diluted when different disease phenotypes and population backgrounds were combined.

From a broader perspective, the findings of this study suggest that SIRT1 gene polymorphisms may indeed be associated with susceptibility to CAD, but that such associations are not universally applicable across all loci, populations, or disease phenotypes. Rather than focusing solely on whether an association exists, future studies should pay more attention to in which phenotypes the association exists, under which genetic models it is observed, and in which population backgrounds it becomes evident. This implies that future research designs should be more refined, not only by increasing sample sizes, but also by standardizing disease definitions, stratifying analyses according to specific clinical phenotypes, and fully considering the modifying effects of ethnicity, metabolic status, and other environmental factors. In addition, statistical associations alone are insufficient to fully explain the biological significance of SIRT1 polymorphisms. Functional studies are still needed to determine whether and to what extent different SNP loci affect SIRT1 expression or activity, and to clarify whether these loci are functionally involved in vascular inflammation, endothelial injury, and plaque formation. Only by integrating genetic associations, molecular functions, and clinical phenotypes can the true role of SIRT1 in the pathogenesis of CAD be more accurately understood. It should also be emphasized that the present study is based on genetic association data rather than functional or mechanistic experiments. Therefore, although previous experimental studies have suggested that SIRT1 is involved in endothelial function, inflammation, oxidative stress, and atherosclerosis, our findings do not establish a direct causal relationship between SIRT1 polymorphisms and these biological processes. The proposed mechanisms should therefore be regarded as potential explanations rather than confirmed pathways and require further experimental validation.

This study has several strengths. First, it systematically integrated the currently available evidence on the relationship between SIRT1 gene polymorphisms and susceptibility to CAD, and conducted separate quantitative analyses for different SNP loci, thereby avoiding the bias that may arise from combining different loci into a single analysis. Second, the included studies were conducted predominantly in Asian and African populations, with very limited representation from European, Latin American, and other ethnic groups. Therefore, the external validity and generalizability of the present findings to the global population remain limited. Third, for loci with relatively high heterogeneity, such as rs7069102, subgroup analyses revealed that differences in disease phenotype may be an important source of heterogeneity, providing a new perspective for understanding inconsistencies in previous findings. Nevertheless, this study has several limitations. First, the number of included studies was relatively small, especially for certain loci and subgroup analyses, some of which were based on only a few studies or even a single study. This limited the stability and generalizability of the pooled estimates. Second, the included studies were mainly conducted in Asian and African populations, with limited data from other ethnic groups, thereby restricting the external validity of the findings. Third, differences in disease phenotype definitions, case inclusion criteria, control sources, study design, and adjustment for confounders may have affected the pooled results. Although CAD- and MI-related phenotypes were integrated from the perspective of the CAD spectrum, these phenotypes represent different pathological stages and clinical characteristics, and the findings should therefore be interpreted in the context of specific disease backgrounds. Fourth, the meta-analysis was mainly based on crude genotype frequencies rather than adjusted effect estimates. Therefore, potential confounding factors, including age, sex, smoking status, hypertension, diabetes mellitus, and dyslipidemia, could not be uniformly controlled across studies. Moreover, some included studies enrolled specific populations, such as patients with type 2 diabetes mellitus, which may have introduced additional clinical heterogeneity. Consequently, the observed associations should be interpreted as genetic susceptibility associations rather than evidence of an independent causal effect of SIRT1 polymorphisms on CAD. Fifth, HWE-based sensitivity analyses showed that some statistically significant associations became non-significant after exclusion of studies deviating from Hardy–Weinberg equilibrium, whereas significant associations emerged for rs7069102 under certain genetic models. These findings suggest that the observed associations may be influenced by study quality, population characteristics, and limited sample size. Sixth, multiple SNPs, genetic models, and subgroup analyses were evaluated, increasing the possibility of type I error. Therefore, nominally significant findings, particularly those with marginal *P* values, should be interpreted cautiously and require validation in larger, well-designed studies. Finally, because the number of included studies for each SNP was limited, formal assessment of publication bias using funnel plots or statistical tests such as Egger's or Begg's test was not appropriate or sufficiently reliable. Nevertheless, small-study effects, selective reporting, and positive publication bias cannot be completely excluded. In addition, because some disease-specific subgroup analyses were based on a limited number of studies and did not completely overlap with population-type subgroup classifications, residual confounding arising from the interaction between disease phenotype and population background may remain. Accordingly, the present findings should be interpreted as evidence of genetic association rather than proof of biological mechanism or causality.

## 5 Conclusion

This systematic review and meta-analysis suggests that SIRT1 gene polymorphisms may be associated with susceptibility to CAD; however, the strength and direction of these associations vary across SNP loci, genetic models, and population subgroups. Among the included polymorphisms, rs7069102 showed no stable association in the overall analysis but exhibited a relatively consistent risk effect in the CAD subgroup; rs7895833 was associated with increased disease risk only under the recessive model; and rs4746720 was associated with increased CAD susceptibility under the dominant and heterozygote models. Overall, these results indicate that the contribution of SIRT1 genetic variation to CAD susceptibility may be locus-specific and may be modified by disease phenotype, ethnic background, metabolic status, and other factors. Because of the limited number of available studies, heterogeneity in population characteristics and disease definitions, multiple statistical comparisons, and the possibility of small-study effects or publication bias, the observed associations should be interpreted cautiously. Larger, multicenter, multiethnic studies with consistent phenotype definitions and appropriate adjustment for clinical confounders are needed, together with functional experiments to clarify the biological relevance of these polymorphisms.

## Data Availability

The original contributions presented in the study are included in the article/Supplementary Material, further inquiries can be directed to the corresponding authors.
