# Supplementary material for: Association between SIRT1 gene polymorphisms and susceptibility to coronary artery disease: a systematic review and meta-analysis
Source: Front Cardiovasc Med. 2026 Jul 3;13:1850297. doi: 10.3389/fcvm.2026.1850297 (PMC13376308; doi:10.3389/fcvm.2026.1850297)
Supplement: Supplementary file 1 [file Table1.docx]

**Supplementary materials 1. Search strategy**

Cochrane search manager search

| **ID** | **Search** | **Hits** |
| --- | --- | --- |
| #1 | MeSH descriptor: [Sirtuin 1] explode all trees | 77 |
| #2 | (Sirt1):ti,ab,kw OR (Silent Mating Type Information Regulation 2 Homolog 1):ti,ab,kw OR (protein SIR2L1):ti,ab,kw OR (protein SIRT1):ti,ab,kw OR (Sir2 like 1 protein):ti,ab,kw | 261 |
| #3 | (Sir2 like protein 1):ti,ab,kw OR (SIR2L1 protein):ti,ab,kw OR (SIRT1 protein):ti,ab,kw OR (Sirtuin 1):ti,ab,kw OR (Sirtuin 1):ti,ab,kw | 279 |
| #4 | MeSH descriptor: [Coronary Disease] explode all trees | 19288 |
| #5 | (Coronary Diseases):ti,ab,kw OR (Disease, Coronary):ti,ab,kw OR (Diseases, Coronary):ti,ab,kw OR (Coronary Heart Disease):ti,ab,kw OR (Disease, Coronary Heart):ti,ab,kw | 46007 |
| #6 | (Diseases, Coronary Heart):ti,ab,kw OR (Heart Disease, Coronary):ti,ab,kw OR (Heart Diseases, Coronary):ti,ab,kw OR (coronary disease):ti,ab,kw OR (multivessel coronary artery disease):ti,ab,kw | 45075 |
| #7 | (Coronary Disease):ti,ab,kw | 44227 |
| #8 | #1 OR #2 OR #3ss | 379 |
| #9 | #4 OR #5 OR #6 OR #7 | 47254 |
| #10 | #8 AND #9 | 30 |

Embase queries

| **No.** | **Query** | **Results** |
| --- | --- | --- |
| #7 | #3 AND #6 | 229 |
| #6 | #4 OR #5 | 568515 |
| #5 | 'coronary diseases':ab,ti OR 'disease, coronary':ab,ti OR 'diseases, coronary':ab,ti OR 'coronary heart disease':ab,ti OR 'disease, coronary heart':ab,ti OR 'diseases, coronary heart':ab,ti OR 'heart disease, coronary':ab,ti OR 'heart diseases, coronary':ab,ti OR 'coronary disease':ab,ti OR 'multivessel coronary artery disease':ab,ti | 115831 |
| #4 | 'coronary artery disease'/exp | 494933 |
| #3 | #1 OR #2 | 27596 |
| #2 | sirt1:ab,ti OR 'silent mating type information regulation 2 homolog 1':ab,ti OR 'protein sir2l1':ab,ti OR 'protein sirt1':ab,ti OR 'sir2 like 1 protein':ab,ti OR 'sir2 like protein 1':ab,ti OR 'sir2l1 protein':ab,ti OR 'sirt1 protein':ab,ti OR 'sirtuin 1':ab,ti | 22134 |
| #1 | 'sirtuin 1'/exp | 23177 |

PubMed Search History

| **Search number** | **Query** | **Sort By** | **Filters** | **Search Details** | **Results** |
| --- | --- | --- | --- | --- | --- |
| 3 | (((((((((((Sirt1[Title/Abstract]) OR (Silent Mating Type Information Regulation 2 Homolog 1[Title/Abstract])) OR (protein SIR2L1[Title/Abstract])) OR (protein SIRT1[Title/Abstract])) OR (Sir2 like 1 protein[Title/Abstract])) OR (Sir2 like protein 1[Title/Abstract])) OR (SIR2L1 protein[Title/Abstract])) OR (SIRT1 protein[Title/Abstract])) OR (Sirtuin 1[Title/Abstract])) OR ("Sirtuin 1"[Mesh]))) AND ((((((((((((Coronary Diseases[Title/Abstract]) OR (Disease, Coronary[Title/Abstract])) OR (Diseases, Coronary[Title/Abstract])) OR (Coronary Heart Disease[Title/Abstract])) OR (Disease, Coronary Heart[Title/Abstract])) OR (Diseases, Coronary Heart[Title/Abstract])) OR (Heart Disease, Coronary[Title/Abstract])) OR (Heart Diseases, Coronary[Title/Abstract])) OR (coronary disease[Title/Abstract])) OR (multivessel coronary artery disease[Title/Abstract])) OR ("Coronary Disease"[Mesh]))) | Most Recent |  | ("sirt1"[Title/Abstract] OR "silent mating type information regulation 2 homolog 1"[Title/Abstract] OR (("protein s"[All Fields] OR "proteinous"[All Fields] OR "proteins"[Supplementary Concept] OR "proteins"[All Fields] OR "protein"[All Fields] OR "proteins"[MeSH Terms]) AND "SIR2L1"[Title/Abstract]) OR "protein sirt1"[Title/Abstract] OR ("Sir2"[All Fields] AND "like 1 protein"[Title/Abstract]) OR "sir2 like protein 1"[Title/Abstract] OR ("SIR2L1"[All Fields] AND "protein"[Title/Abstract]) OR "sirt1 protein"[Title/Abstract] OR "Sirtuin 1"[Title/Abstract] OR "Sirtuin 1"[MeSH Terms]) AND ("coronary diseases"[Title/Abstract] OR "disease coronary"[Title/Abstract] OR "diseases coronary"[Title/Abstract] OR "coronary heart disease"[Title/Abstract] OR "disease coronary heart"[Title/Abstract] OR "diseases coronary heart"[Title/Abstract] OR "heart disease coronary"[Title/Abstract] OR "heart diseases coronary"[Title/Abstract] OR "Coronary Disease"[Title/Abstract] OR "multivessel coronary artery disease"[Title/Abstract] OR "Coronary Disease"[MeSH Terms]) | 87 |
| 2 | (((((((((((Coronary Diseases[Title/Abstract]) OR (Disease, Coronary[Title/Abstract])) OR (Diseases, Coronary[Title/Abstract])) OR (Coronary Heart Disease[Title/Abstract])) OR (Disease, Coronary Heart[Title/Abstract])) OR (Diseases, Coronary Heart[Title/Abstract])) OR (Heart Disease, Coronary[Title/Abstract])) OR (Heart Diseases, Coronary[Title/Abstract])) OR (coronary disease[Title/Abstract])) OR (multivessel coronary artery disease[Title/Abstract])) OR ("Coronary Disease"[Mesh])) | Most Recent |  | "coronary diseases"[Title/Abstract] OR "disease coronary"[Title/Abstract] OR "diseases coronary"[Title/Abstract] OR "coronary heart disease"[Title/Abstract] OR "disease coronary heart"[Title/Abstract] OR "diseases coronary heart"[Title/Abstract] OR "heart disease coronary"[Title/Abstract] OR "heart diseases coronary"[Title/Abstract] OR "Coronary Disease"[Title/Abstract] OR "multivessel coronary artery disease"[Title/Abstract] OR "Coronary Disease"[MeSH Terms] | 285,015 |
| 1 | ((((((((((Sirt1[Title/Abstract]) OR (Silent Mating Type Information Regulation 2 Homolog 1[Title/Abstract])) OR (protein SIR2L1[Title/Abstract])) OR (protein SIRT1[Title/Abstract])) OR (Sir2 like 1 protein[Title/Abstract])) OR (Sir2 like protein 1[Title/Abstract])) OR (SIR2L1 protein[Title/Abstract])) OR (SIRT1 protein[Title/Abstract])) OR (Sirtuin 1[Title/Abstract])) OR ("Sirtuin 1"[Mesh])) | Most Recent |  | "sirt1"[Title/Abstract] OR "silent mating type information regulation 2 homolog 1"[Title/Abstract] OR (("protein s"[All Fields] OR "proteinous"[All Fields] OR "proteins"[Supplementary Concept] OR "proteins"[All Fields] OR "protein"[All Fields] OR "proteins"[MeSH Terms]) AND "SIR2L1"[Title/Abstract]) OR "protein sirt1"[Title/Abstract] OR ("Sir2"[All Fields] AND "like 1 protein"[Title/Abstract]) OR "sir2 like protein 1"[Title/Abstract] OR ("SIR2L1"[All Fields] AND "protein"[Title/Abstract]) OR "sirt1 protein"[Title/Abstract] OR "Sirtuin 1"[Title/Abstract] OR "Sirtuin 1"[MeSH Terms] | 18,161 |

WOS search history

| **#** | **Search Query** | **Database** | **Results** |
| --- | --- | --- | --- |
| 1 | ((((((((TS=(Sirt1)) OR TS=(Silent Mating Type Information Regulation 2 Homolog 1)) OR TS=(protein SIR2L1)) OR TS=(protein SIRT1)) OR TS=(Sir2 like 1 protein)) OR TS=(Sir2 like protein 1)) OR TS=(SIR2L1 protein)) OR TS=(SIRT1 protein)) OR TS=(Sirtuin 1) | Web of Science Core Collection | 22811 |
| 2 | (((((((((TS=(Coronary Diseases)) OR TS=(Disease, Coronary)) OR TS=(Diseases, Coronary)) OR TS=(Coronary Heart Disease)) OR TS=(Disease, Coronary Heart)) OR TS=(Diseases, Coronary Heart)) OR TS=(Heart Disease, Coronary)) OR TS=(Heart Diseases, Coronary)) OR TS=(coronary disease)) OR TS=(multivessel coronary artery disease) | Web of Science Core Collection | 392741 |
| 3 | #1 AND #2 | Web of Science Core Collection | 267 |
