# Supplementary material for: Association between SIRT1 gene polymorphisms and susceptibility to coronary artery disease: a systematic review and meta-analysis
Source: Front Cardiovasc Med. 2026 Jul 3;13:1850297. doi: 10.3389/fcvm.2026.1850297 (PMC13376308; doi:10.3389/fcvm.2026.1850297)

**Supplementary Material 3. Meta-analysis results for rs7069102 (Exp = G)**

**Allelic model (G vs C)**

**Overall meta-analysis for rs7069102 under the allelic model (G vs C).**


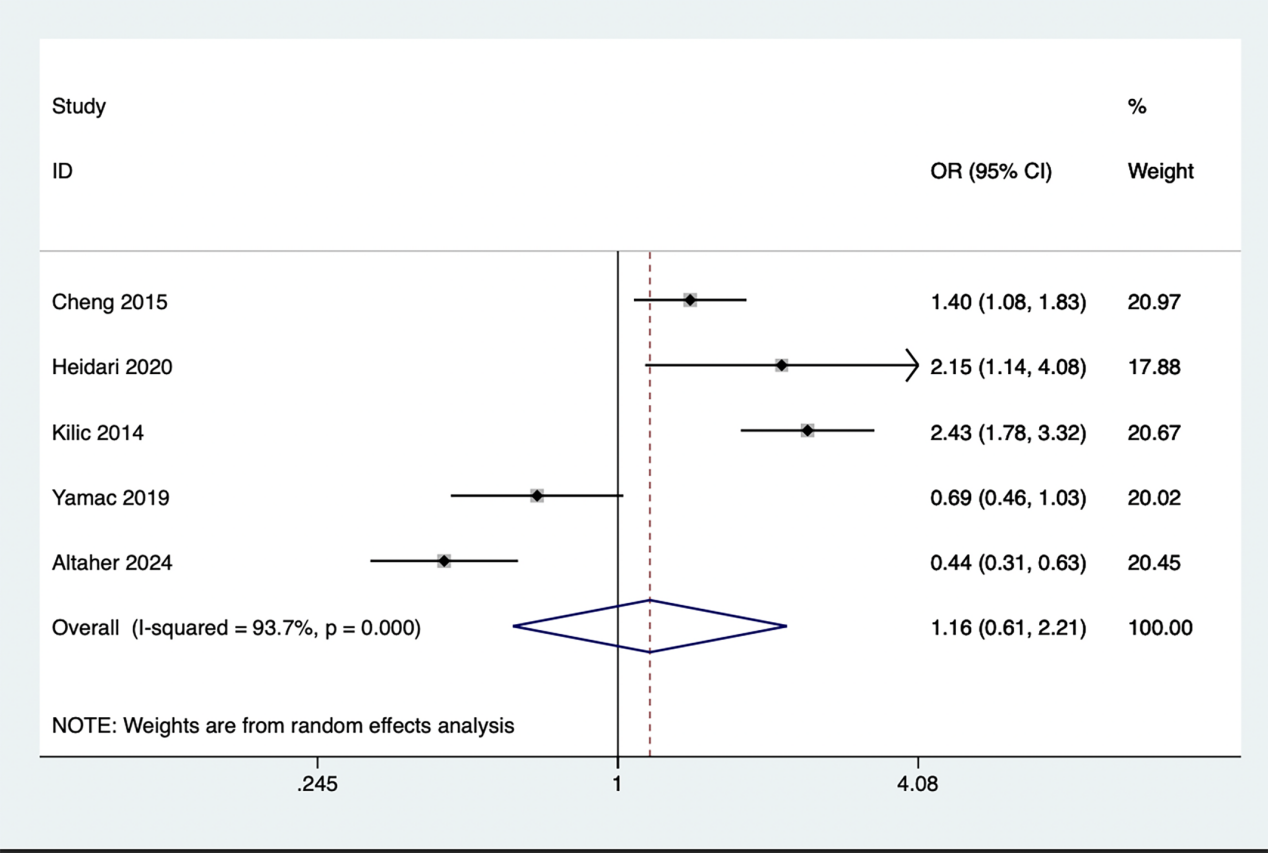


**Sensitivity analysis for rs7069102 under the allelic model (G vs C).**


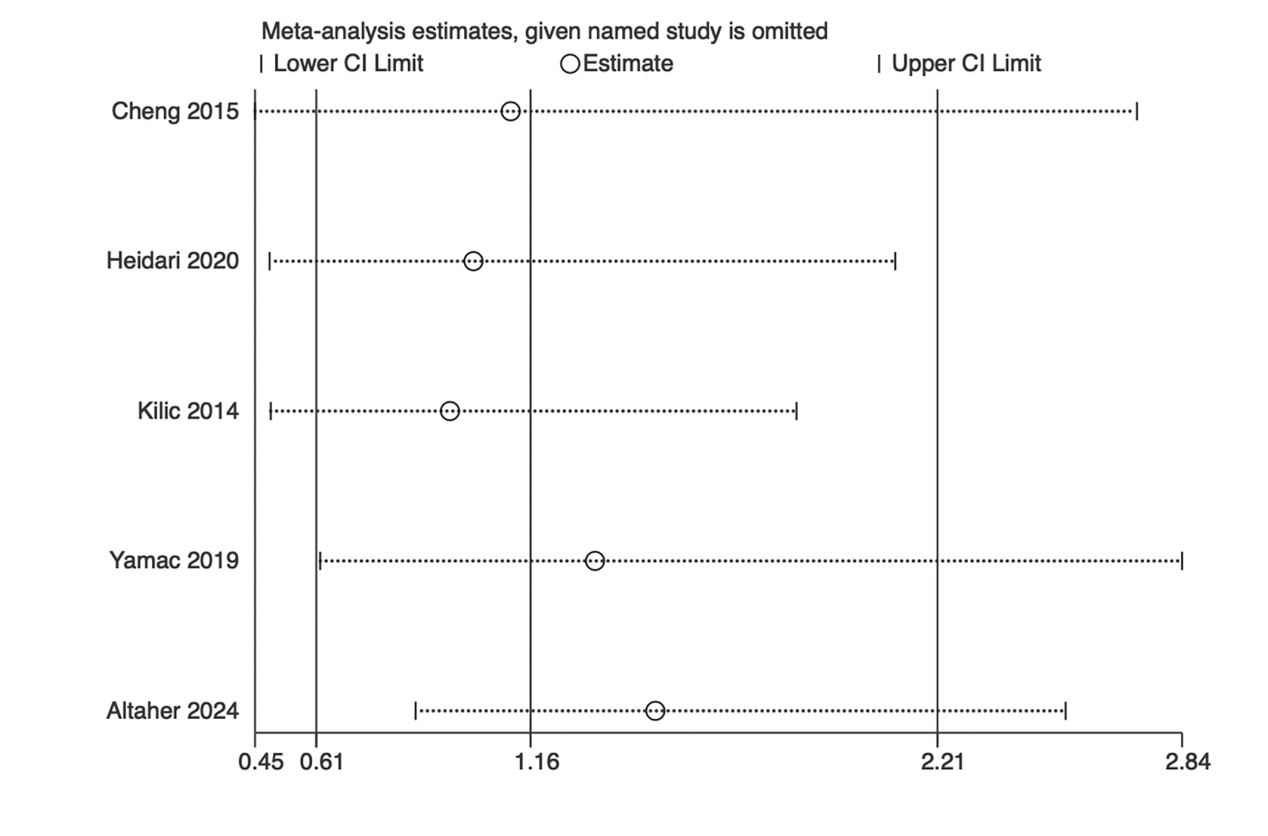


**Population subgroup analysis for rs7069102 under the allelic model (G vs C).**


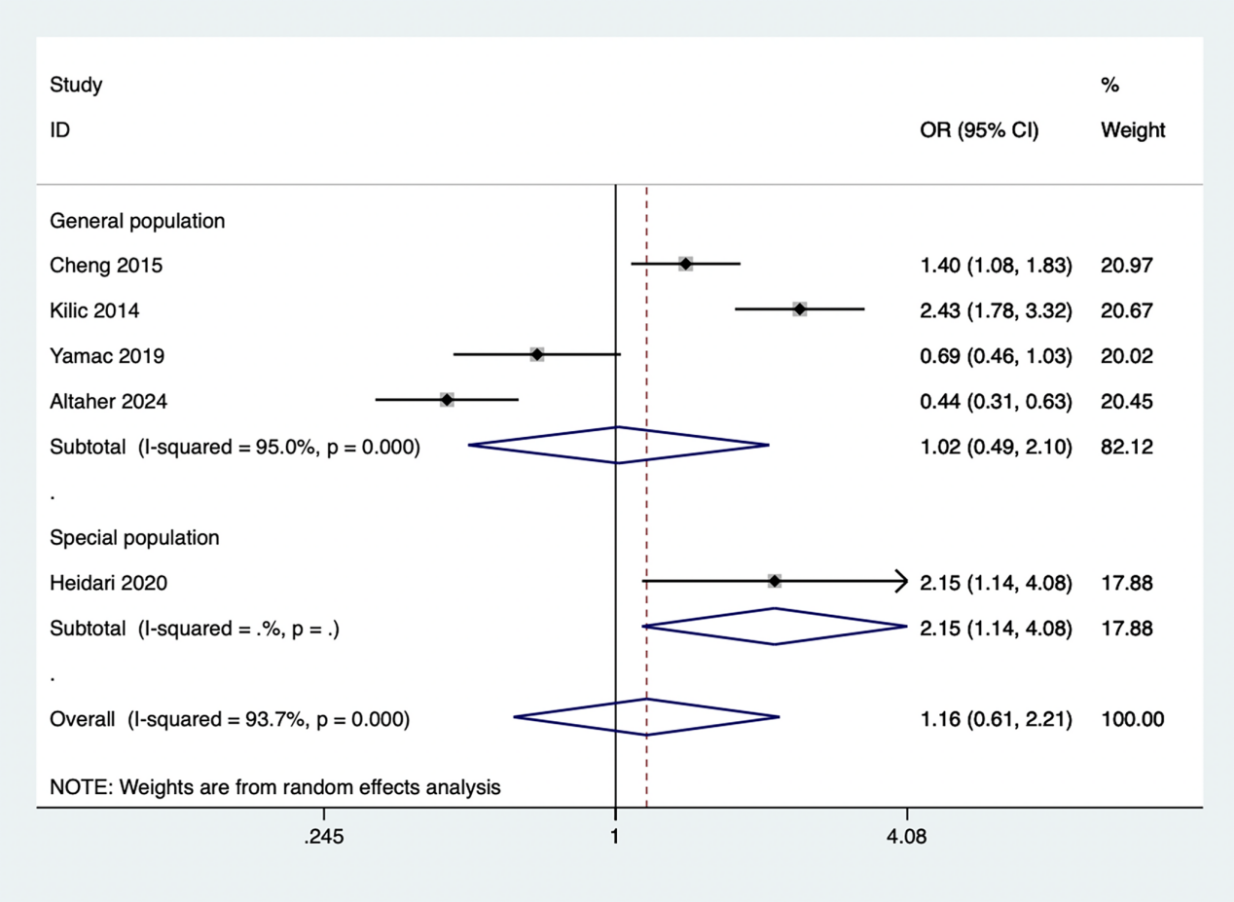


**Disease subgroup analysis: MI for rs7069102 under the allelic model (G vs C).**


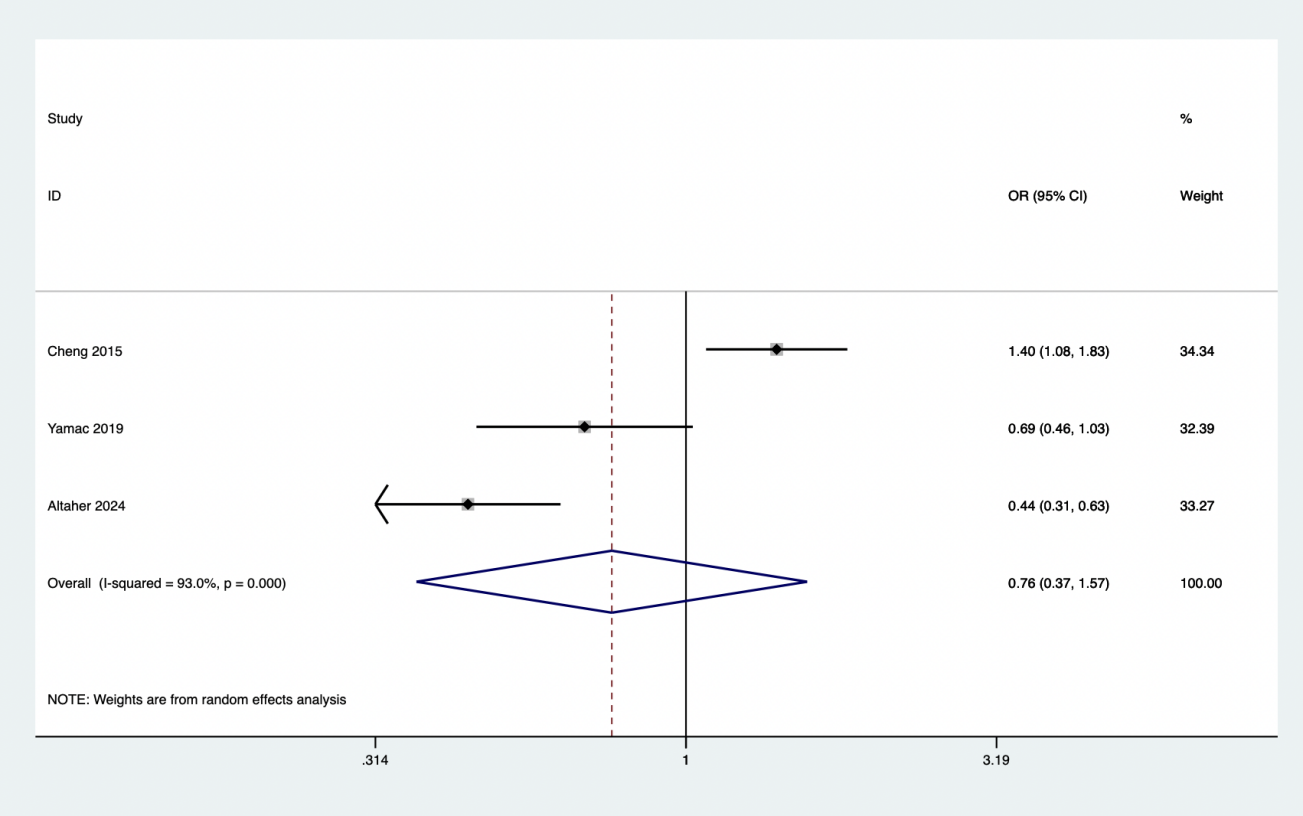


**Disease subgroup analysis: CAD for rs7069102 under the allelic model (G vs C).**


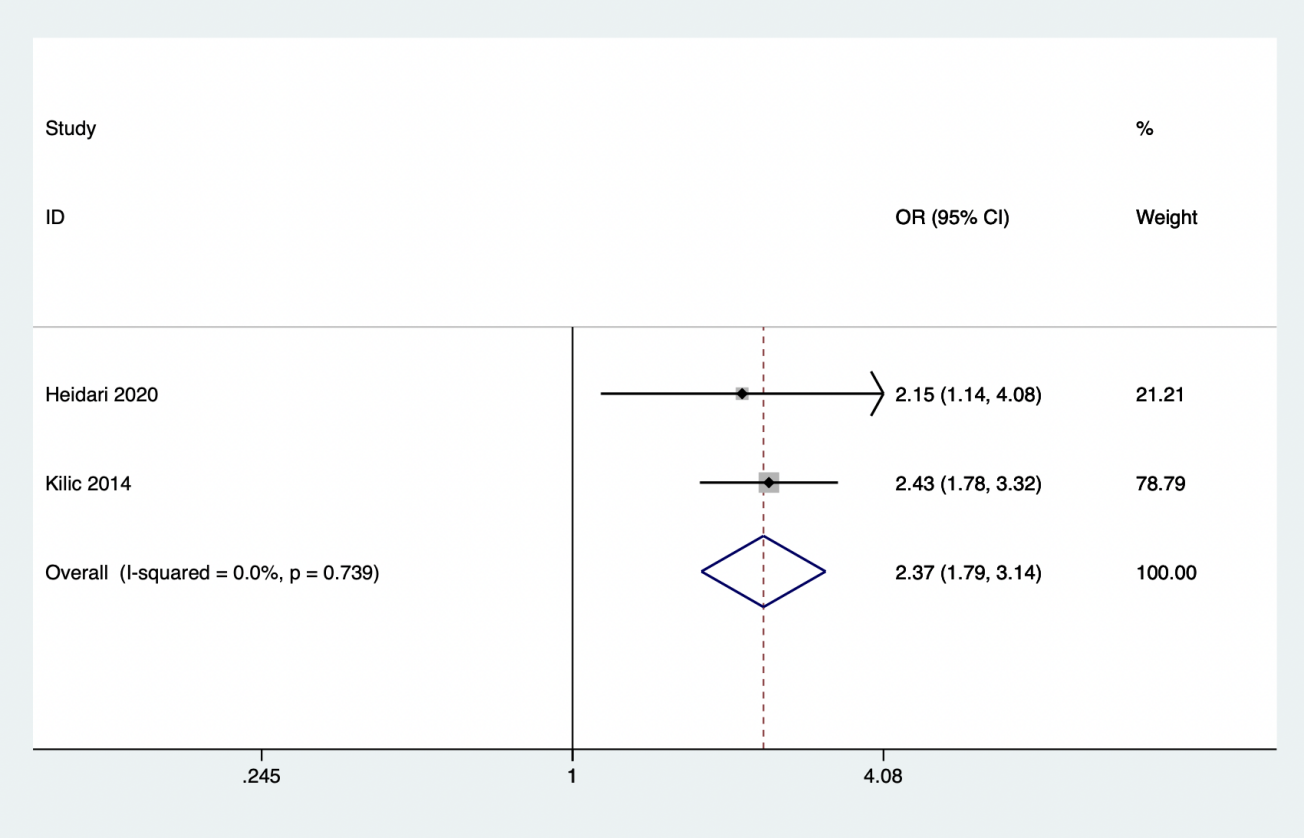


**Ethnicity subgroup analysis for rs7069102 under the allelic model (G vs C).**


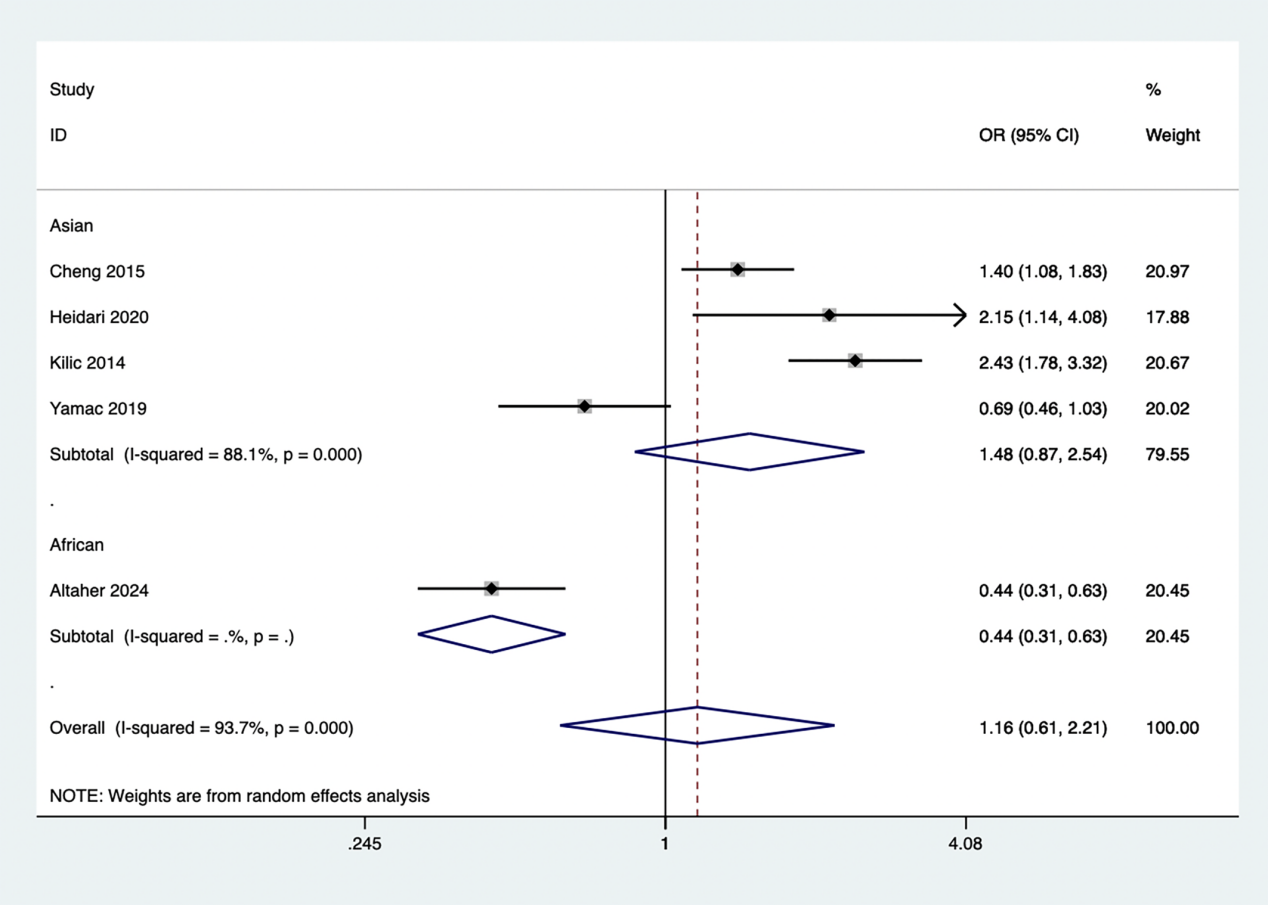


**Study design subgroup analysis for rs7069102 under the allelic model (G vs C).**


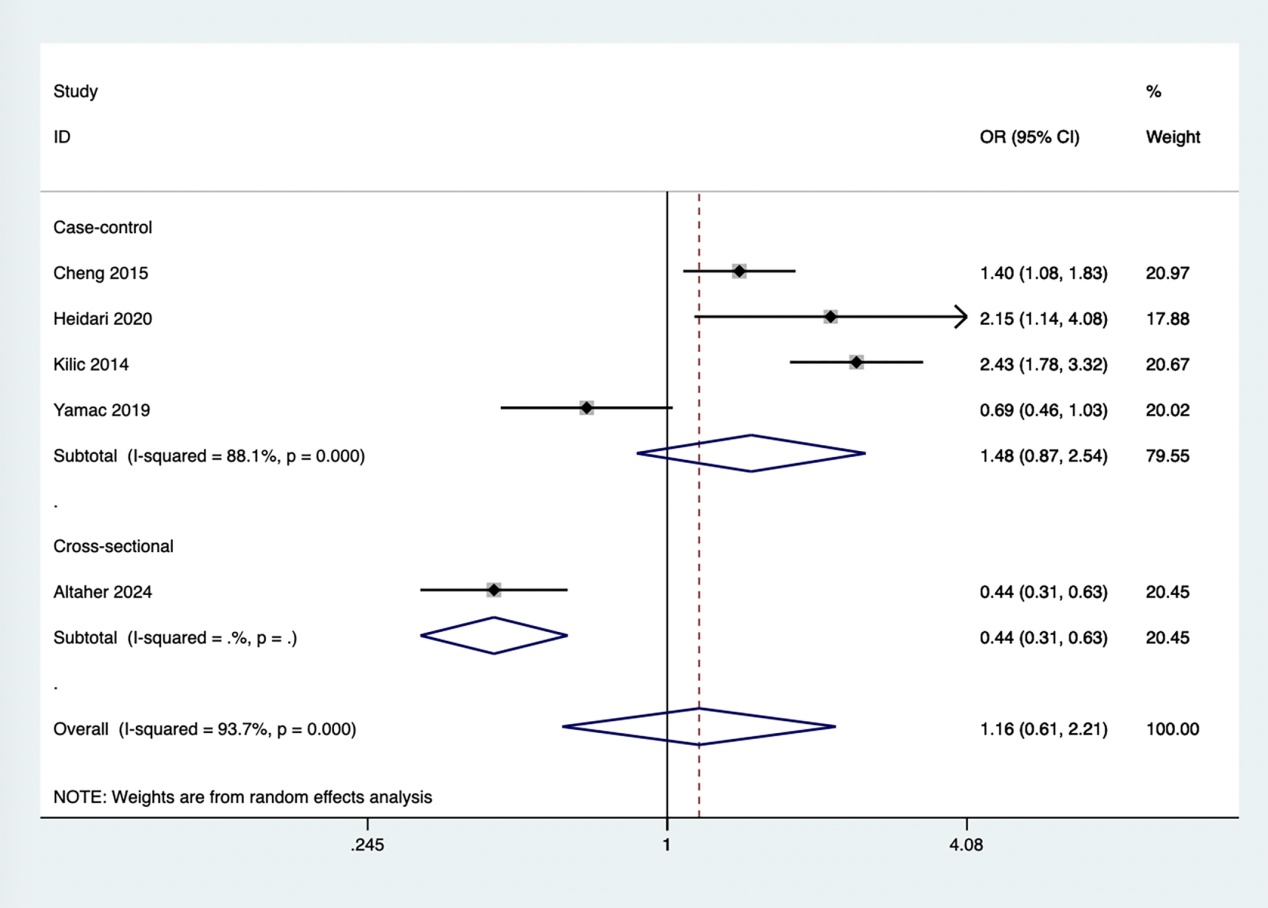


**Dominant model (GG+CG vs CC)**

**Overall meta-analysis for rs7069102 under the dominant model (GG+CG vs CC).**


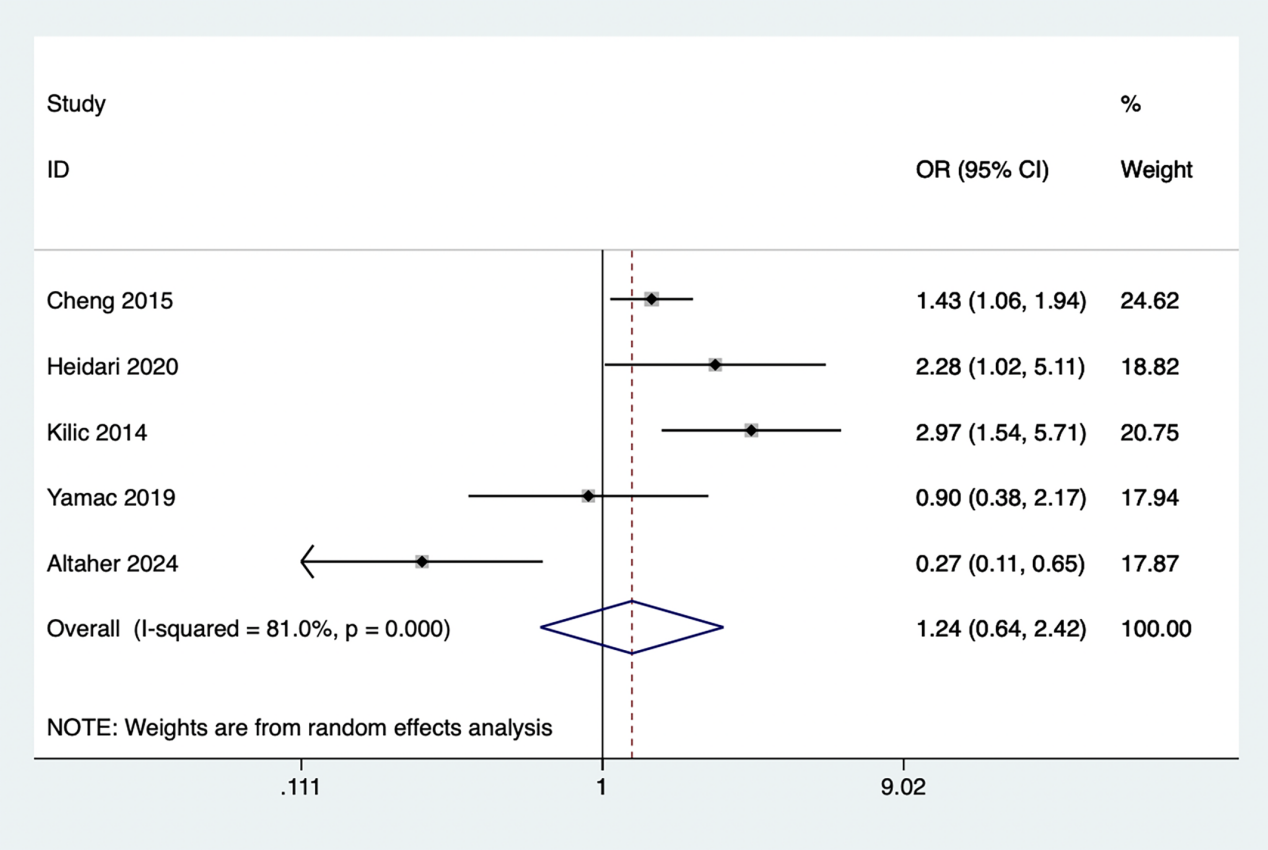


**Sensitivity analysis for rs7069102 under the dominant model (GG+CG vs CC).**


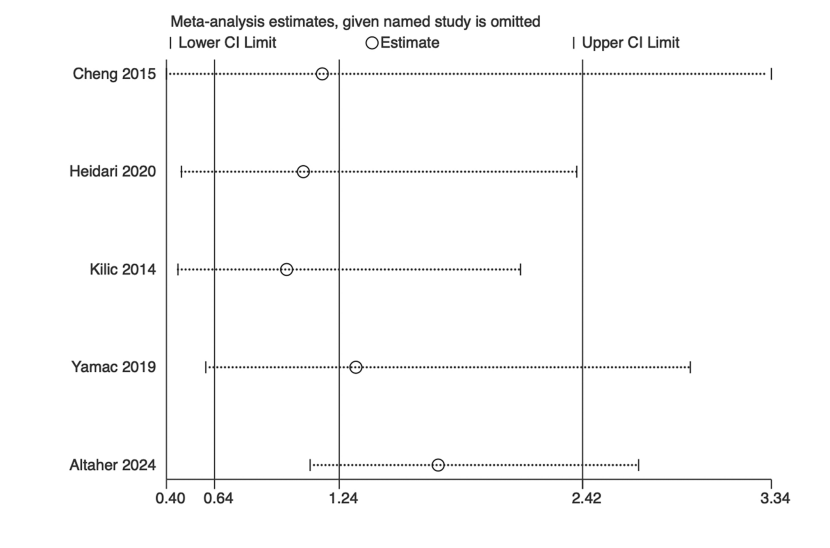


**Population subgroup analysis for rs7069102 under the dominant model (GG+CG vs CC).**


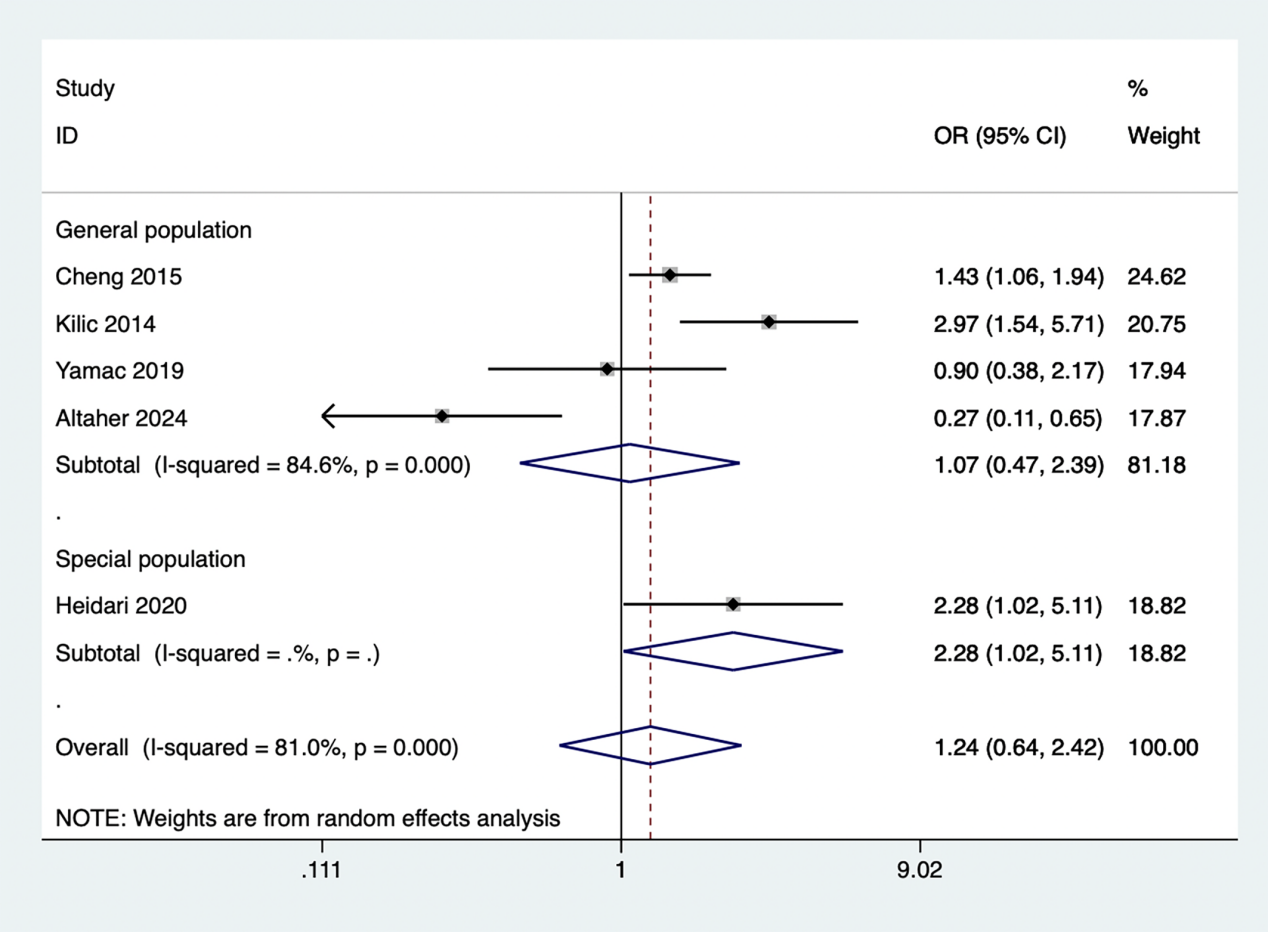


**Disease subgroup analysis: MI for rs7069102 under the dominant model (GG+CG vs CC).**


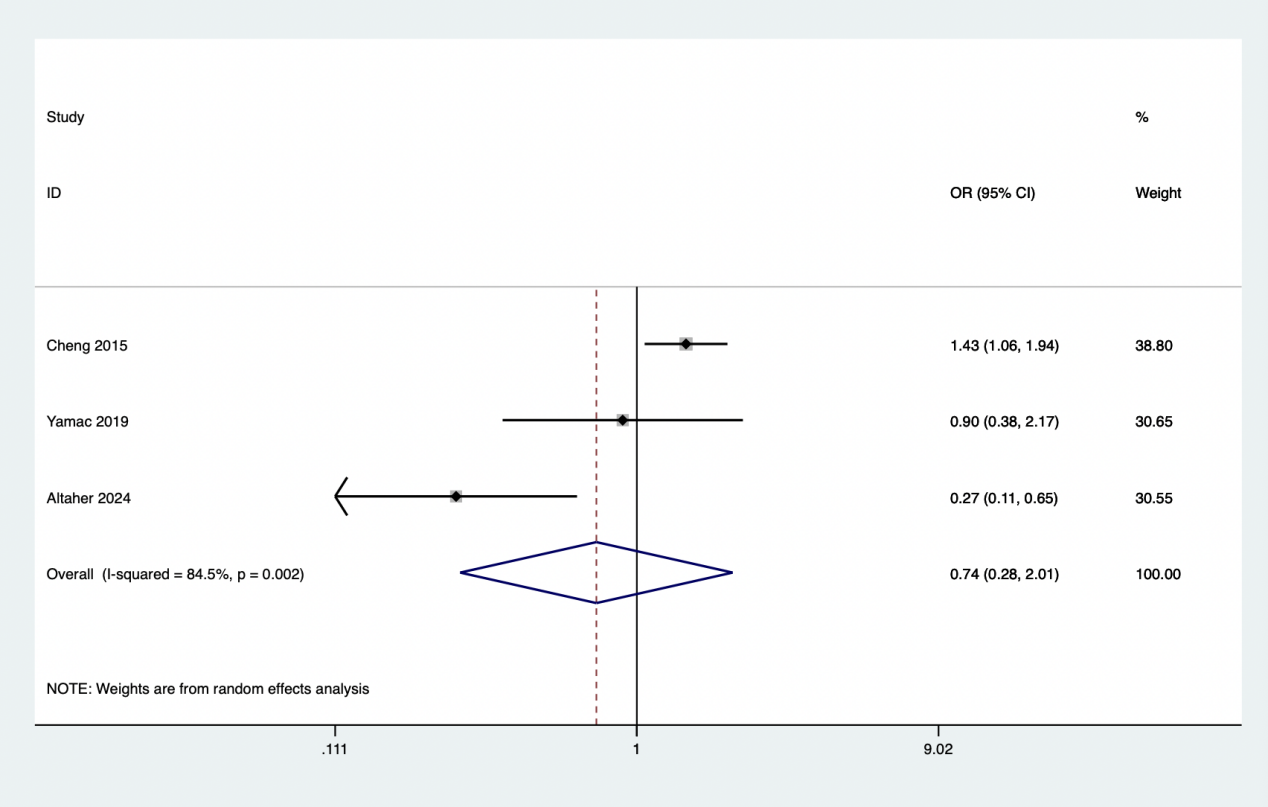


**Disease subgroup analysis: CAD for rs7069102 under the dominant model (GG+CG vs CC).**


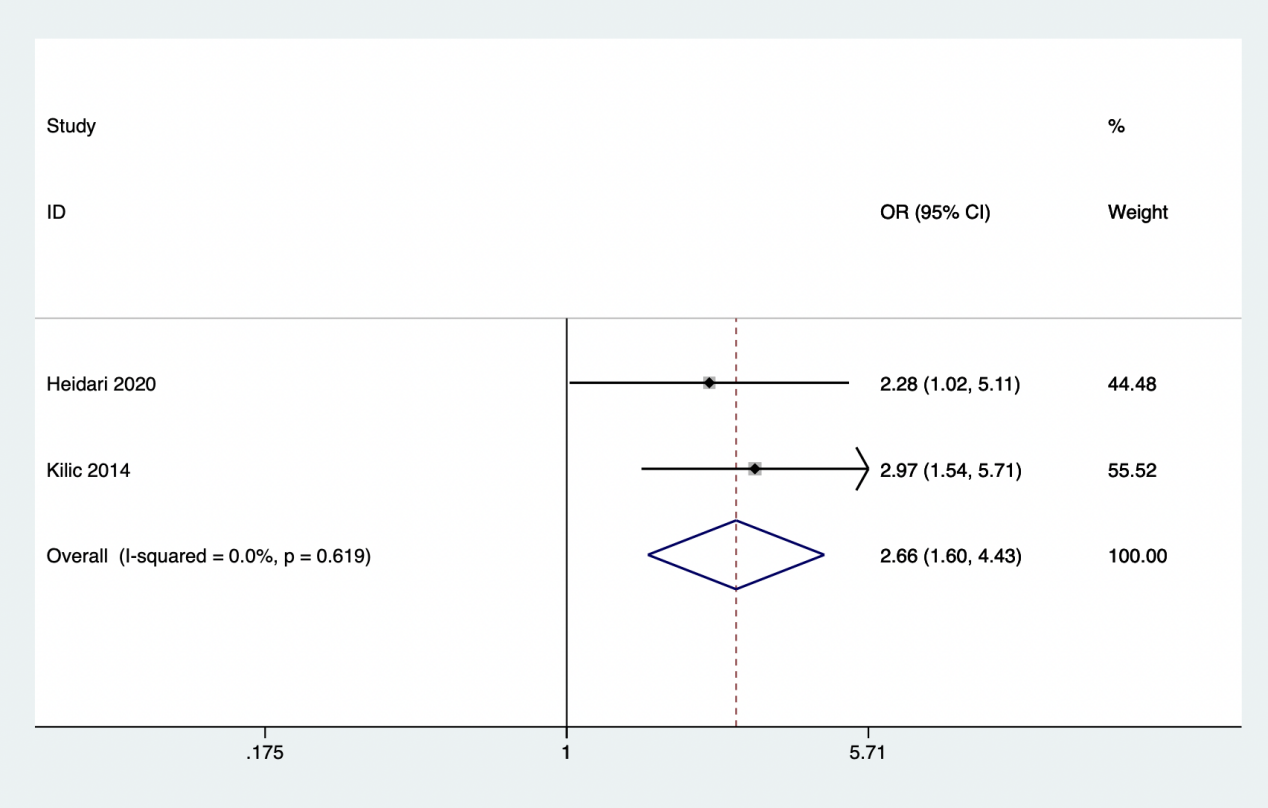


**Ethnicity subgroup analysis for rs7069102 under the dominant model (GG+CG vs CC).**


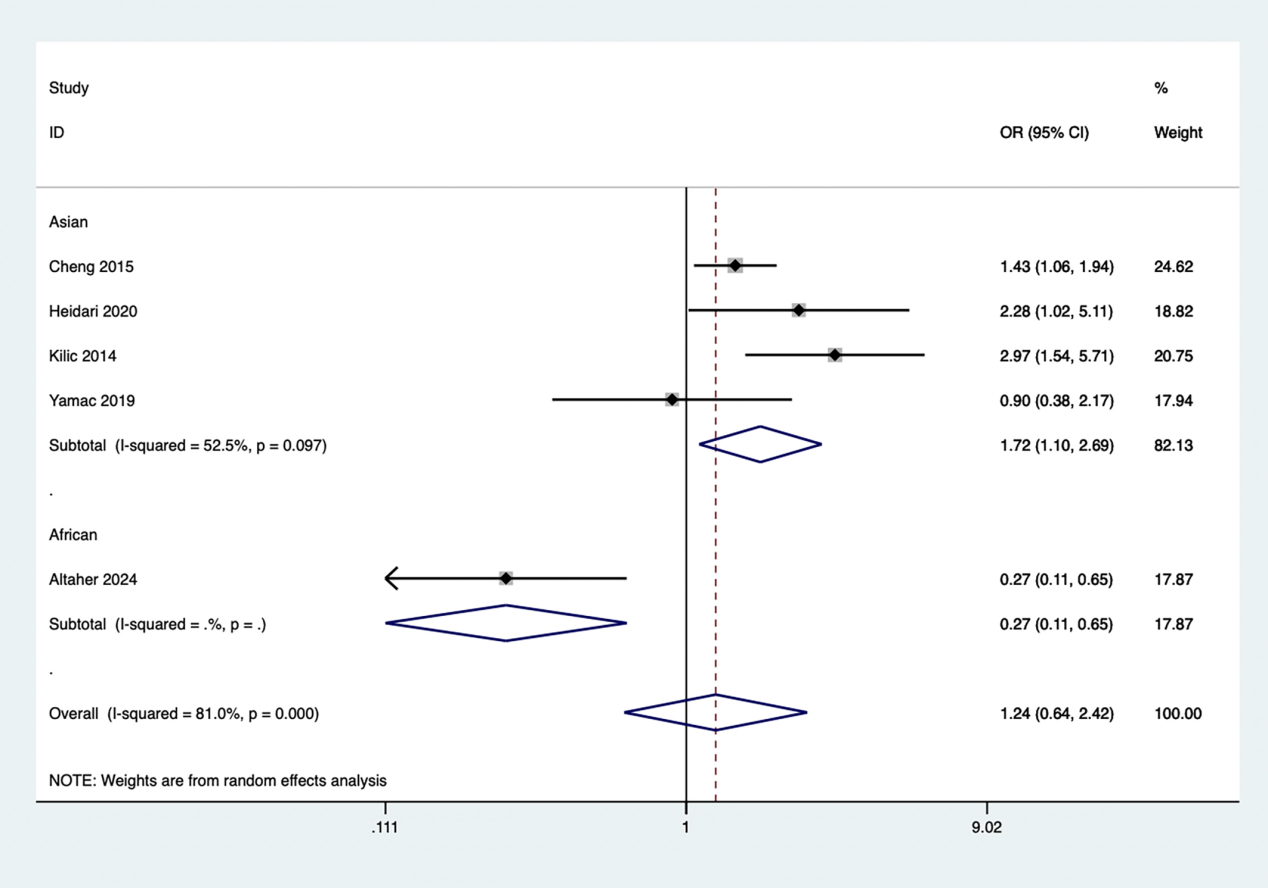


**Study design subgroup analysis for rs7069102 under the dominant model (GG+CG vs CC).**


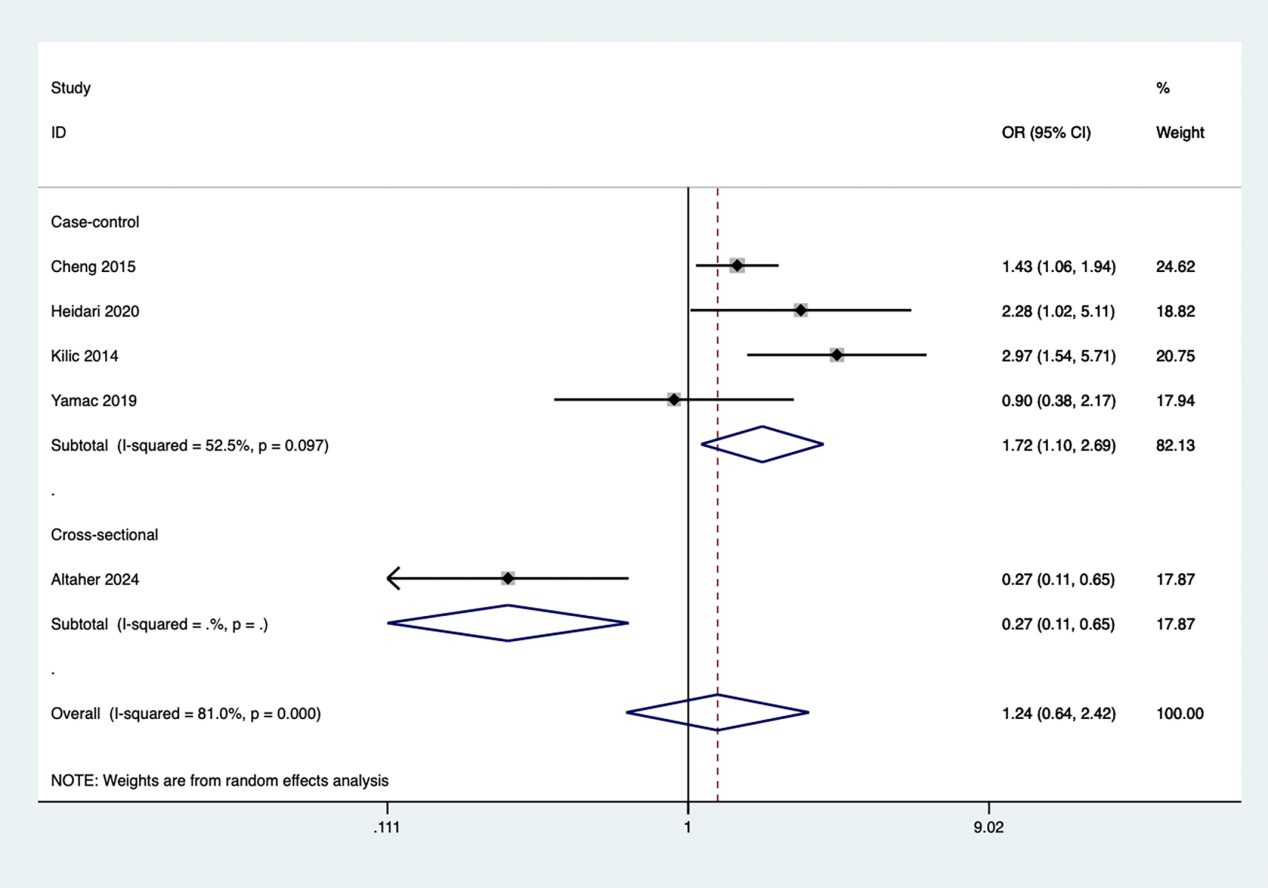


**Recessive model (GG vs CG+CC)**

**Overall meta-analysis for rs7069102 under the recessive model (GG vs CG+CC).**


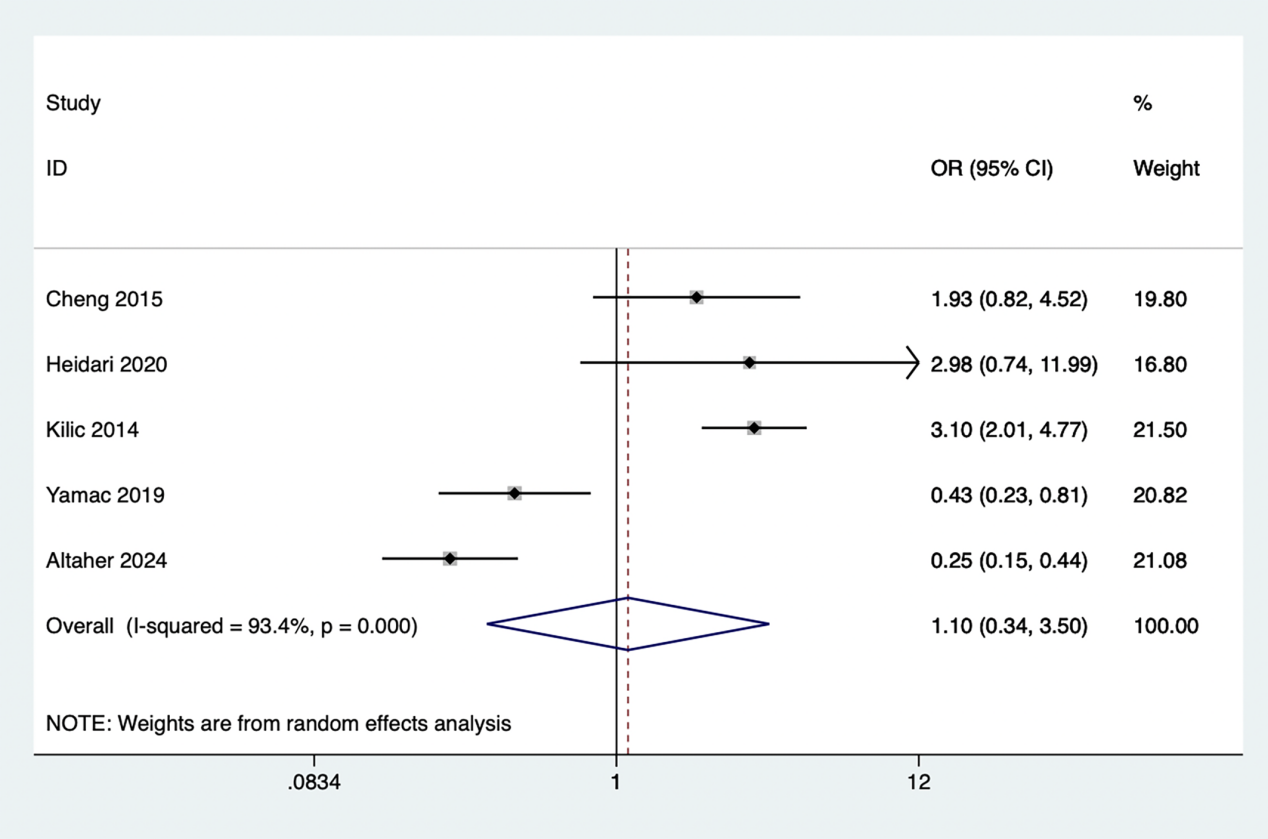


**Sensitivity analysis for rs7069102 under the recessive model (GG vs CG+CC).**


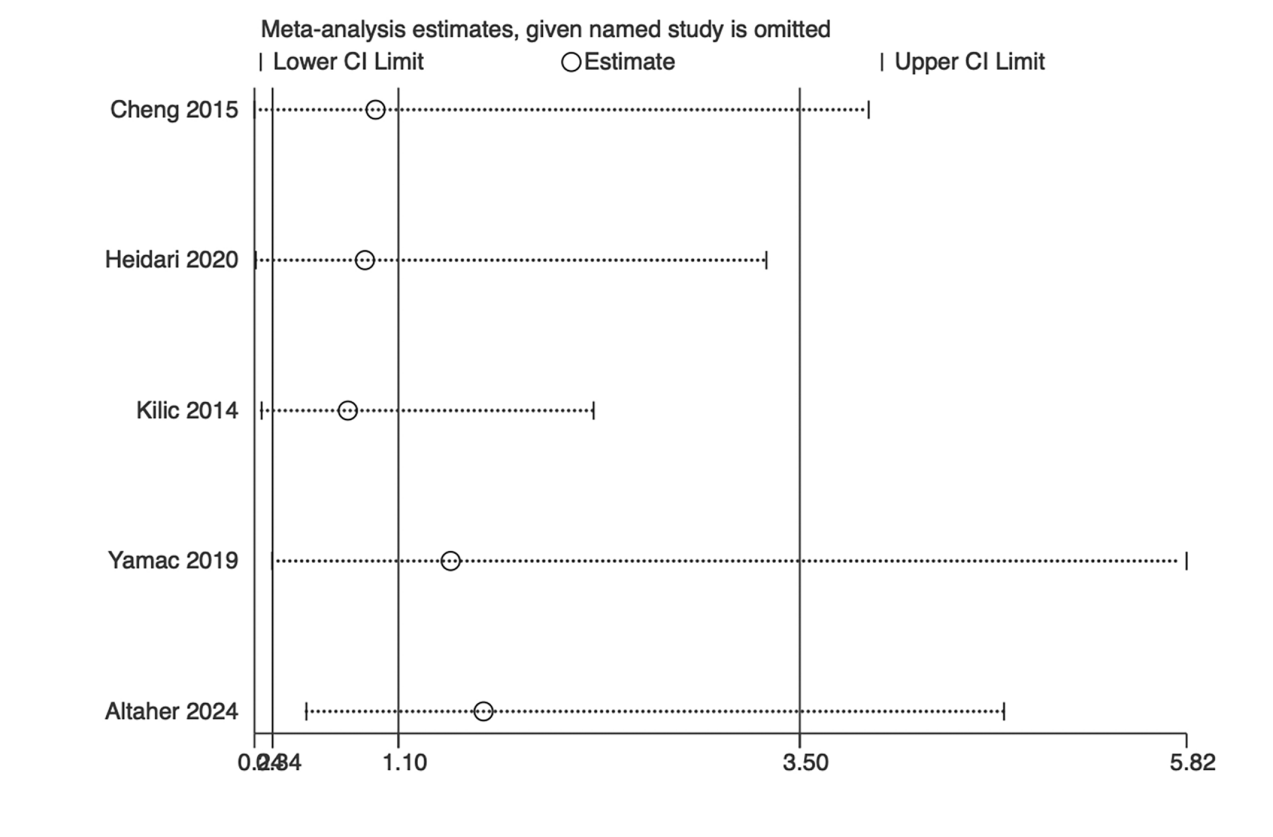


**Population subgroup analysis for rs7069102 under the recessive model (GG vs CG+CC).**


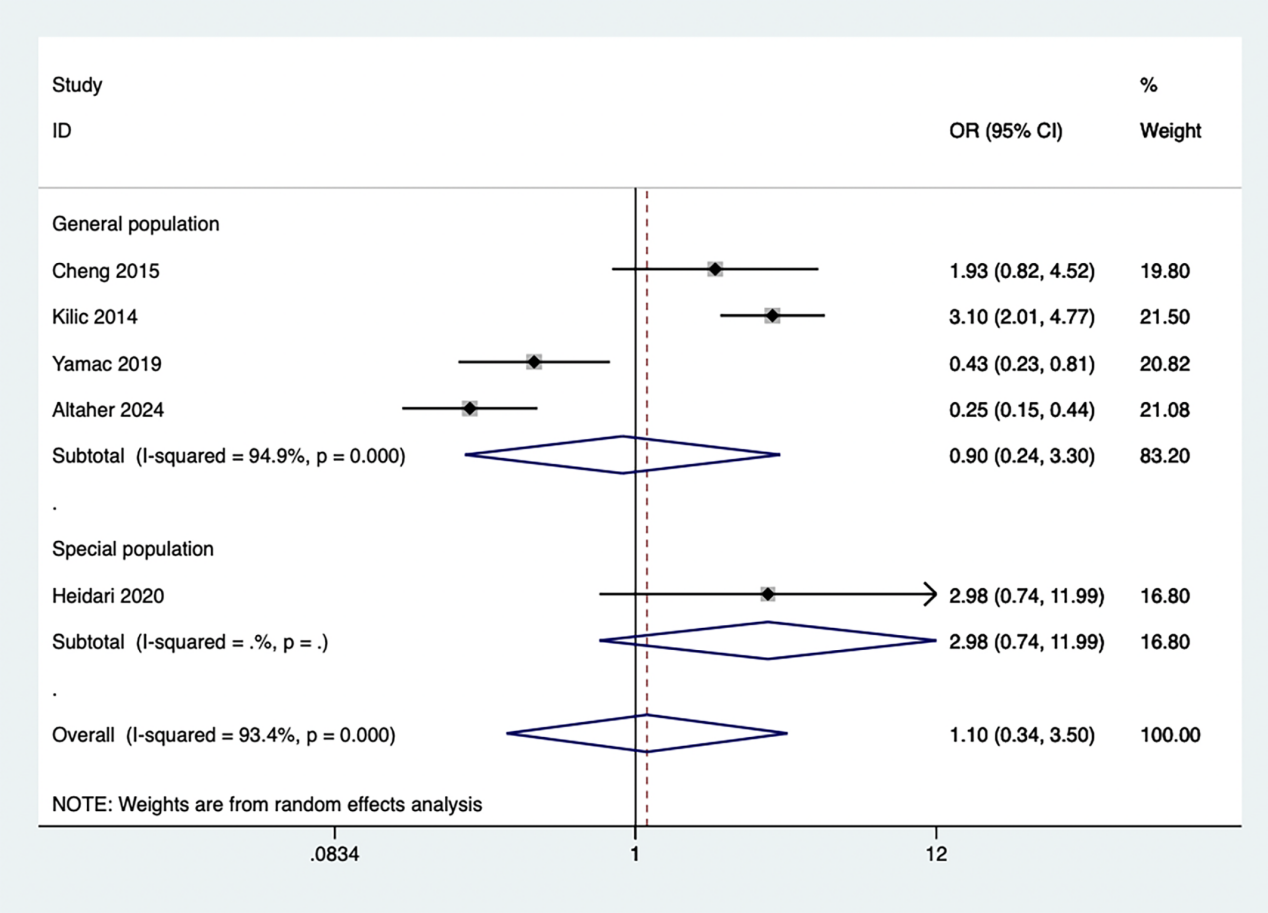


**Disease subgroup analysis: MI for rs7069102 under the recessive model (GG vs CG+CC).**


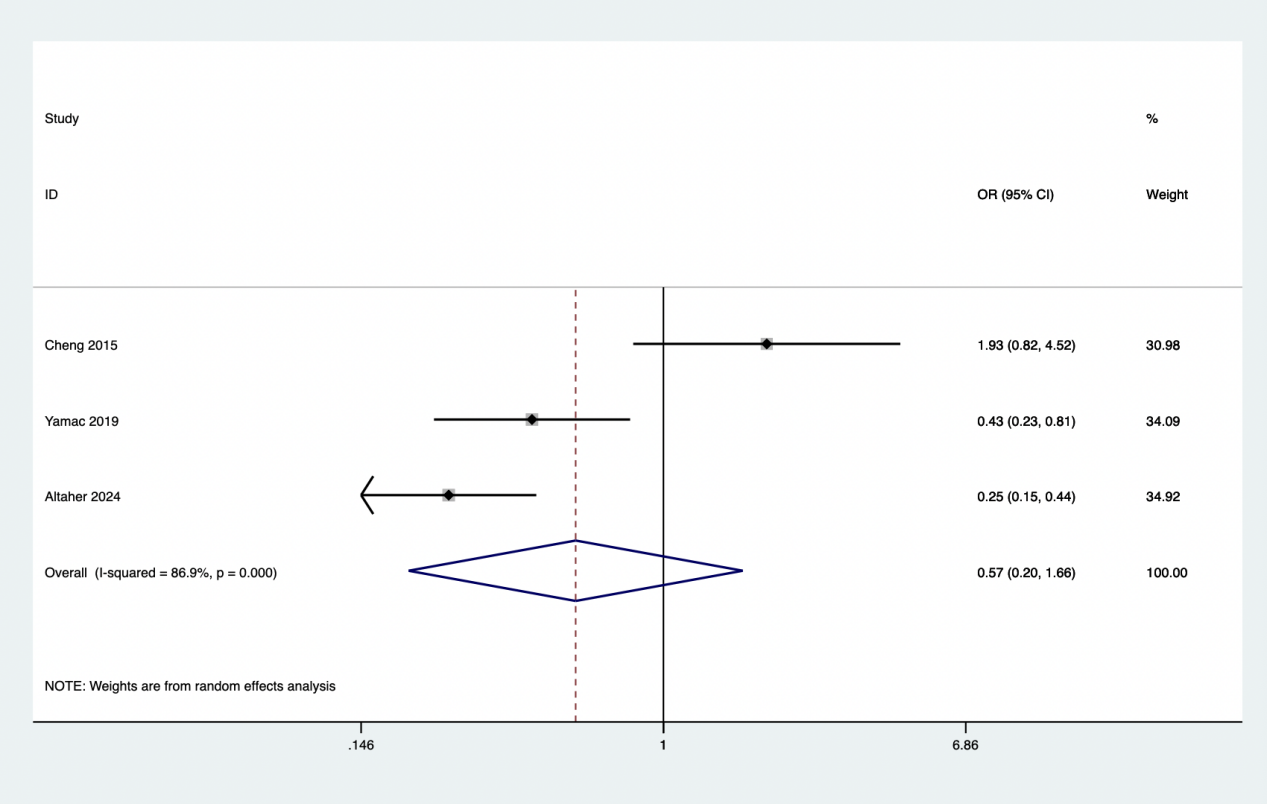


**Disease subgroup analysis: CAD for rs7069102 under the recessive model (GG vs CG+CC).**


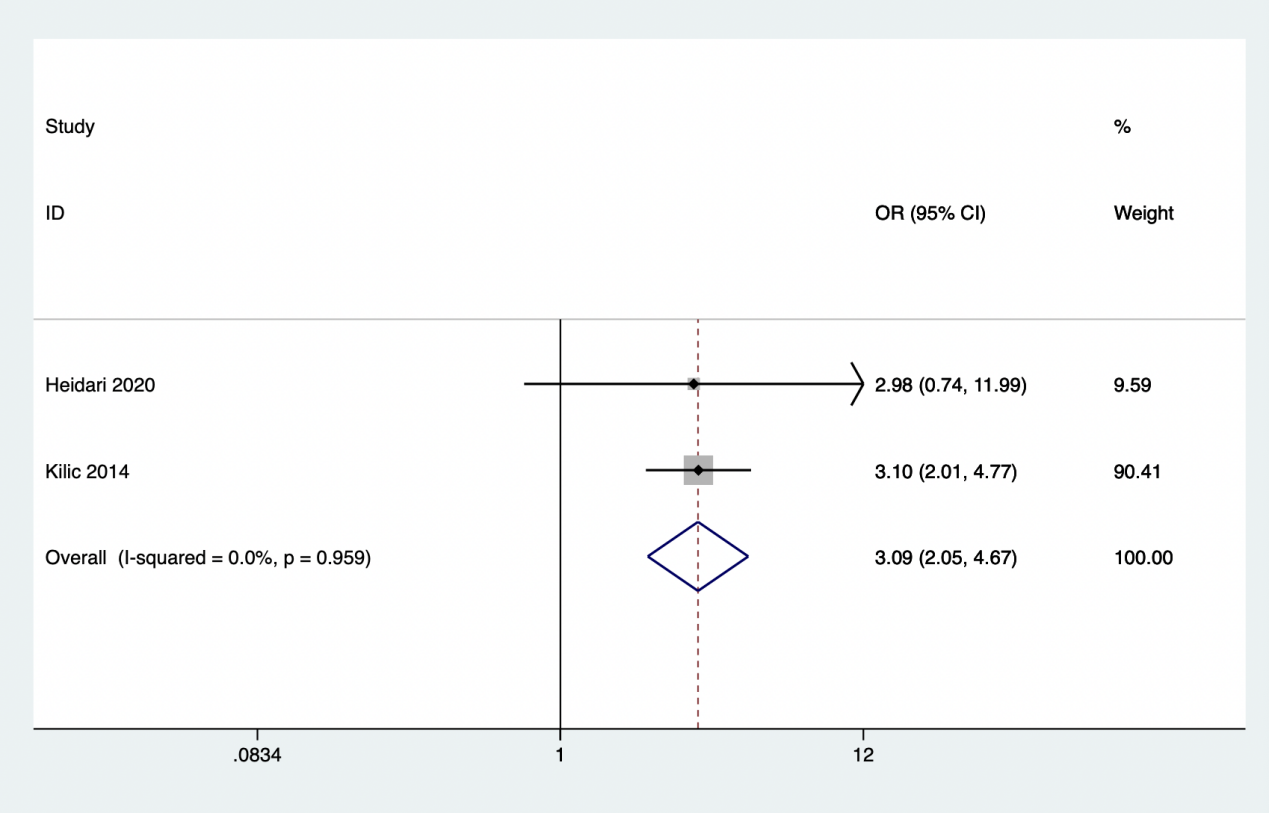


**Ethnicity subgroup analysis for rs7069102 under the recessive model (GG vs CG+CC).**


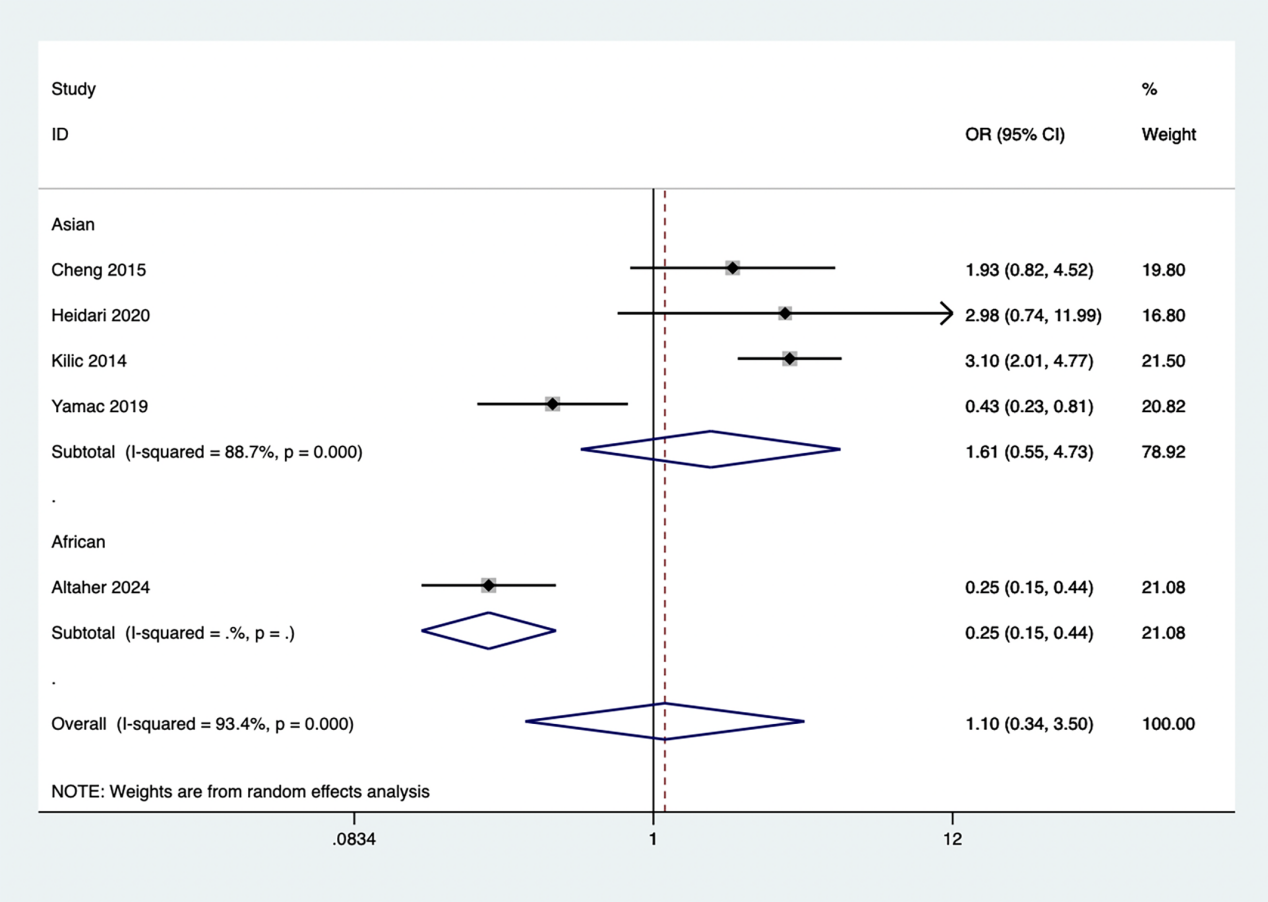


**Study design subgroup analysis for rs7069102 under the recessive model (GG vs CG+CC).**


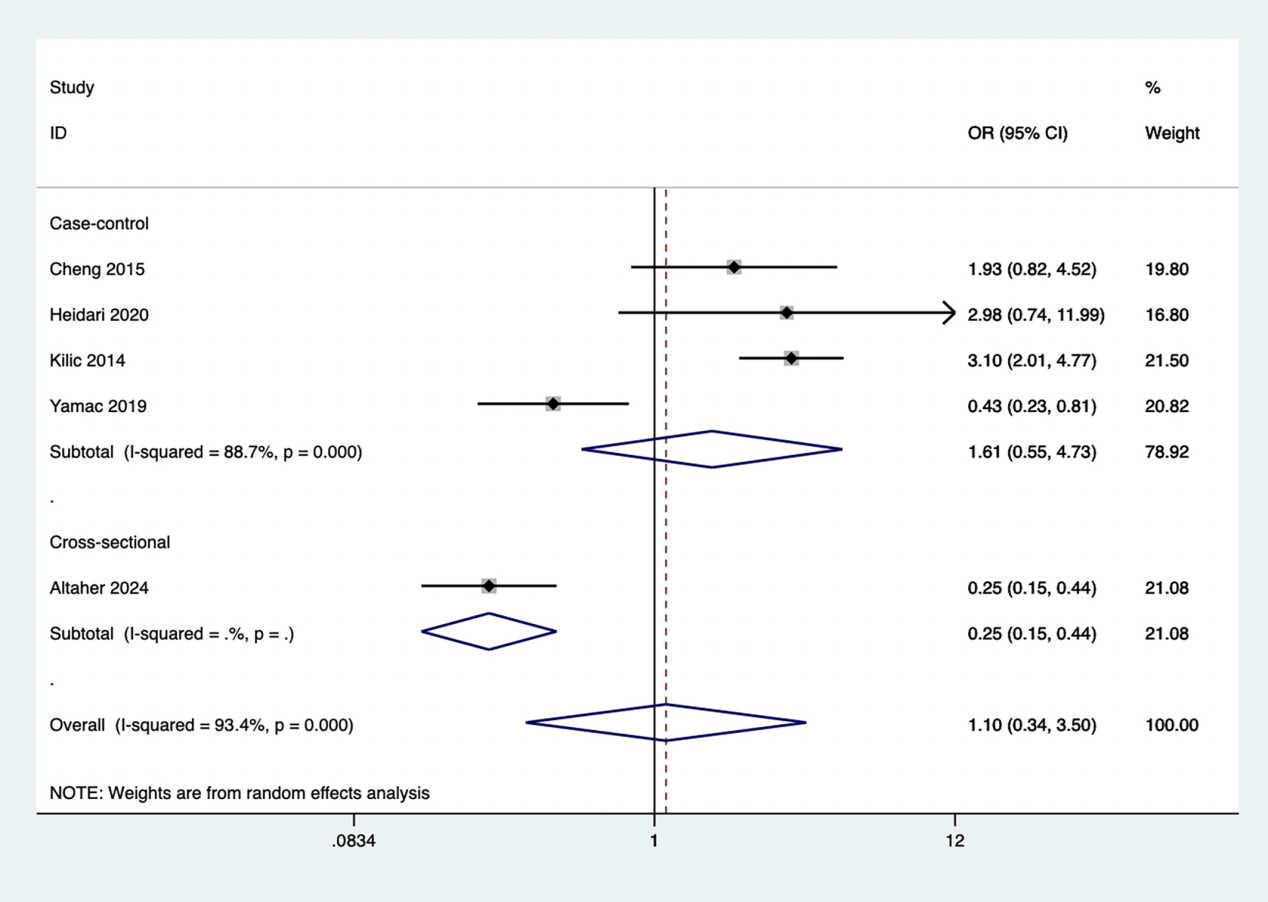


**Homozygote model (GG vs CC)**

**Overall meta-analysis for rs7069102 under the homozygote model (GG vs CC).**


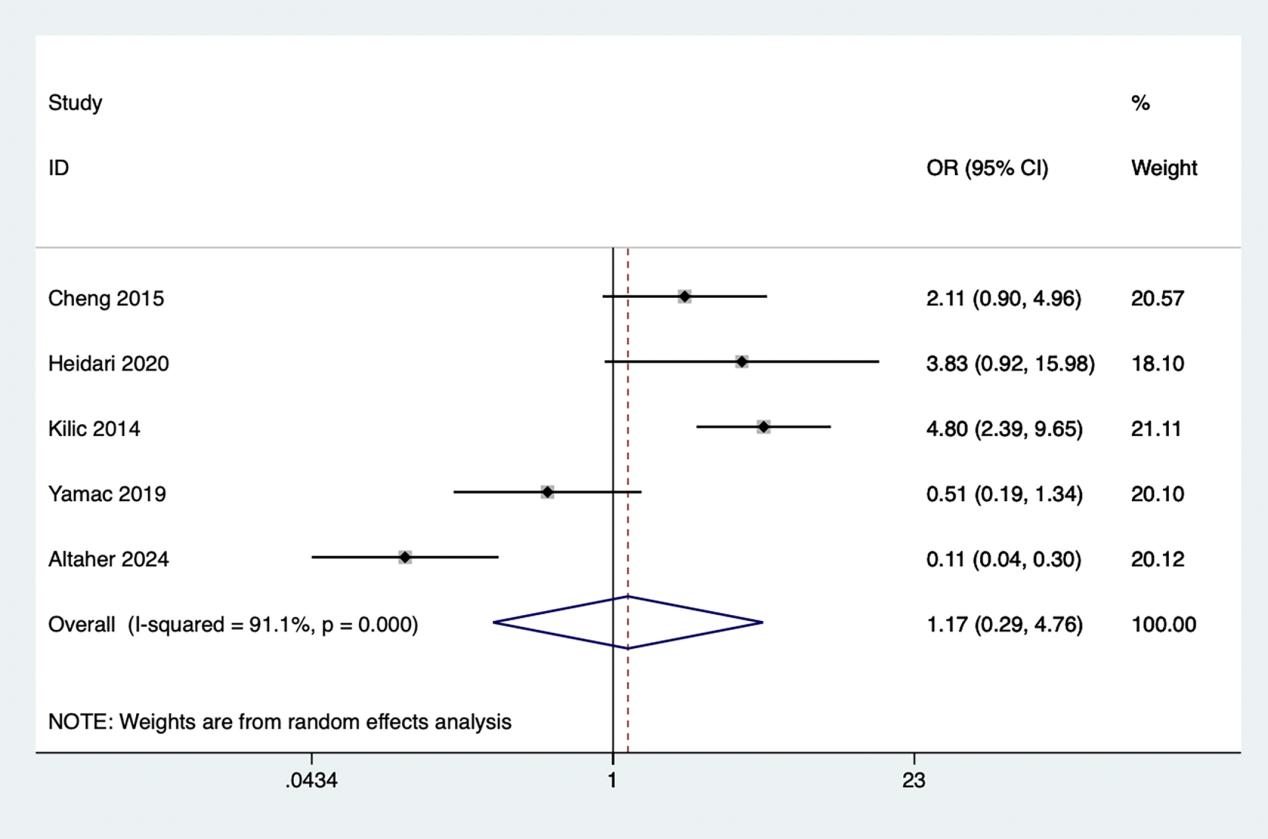


**Sensitivity analysis for rs7069102 under the homozygote model (GG vs CC).**


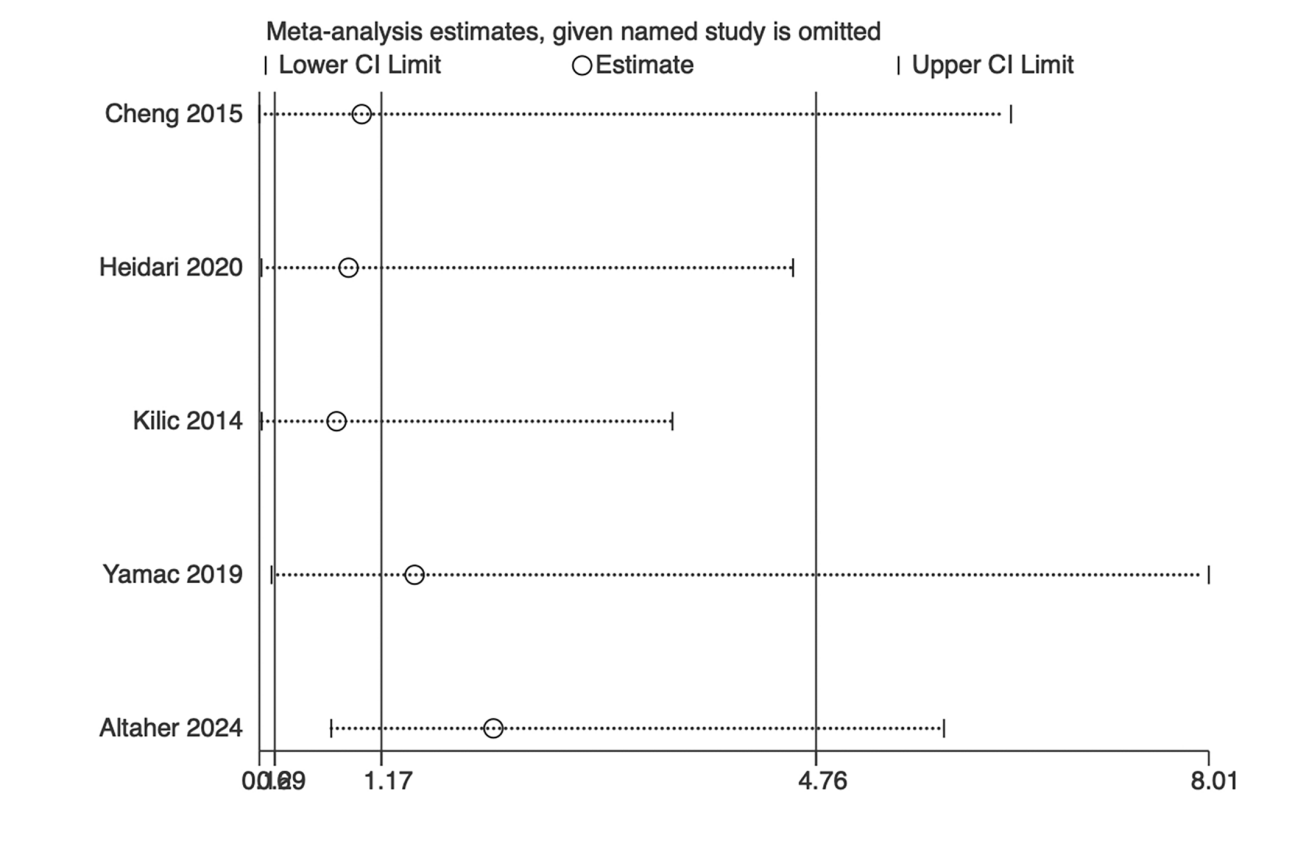


**Population subgroup analysis for rs7069102 under the homozygote model (GG vs CC).**


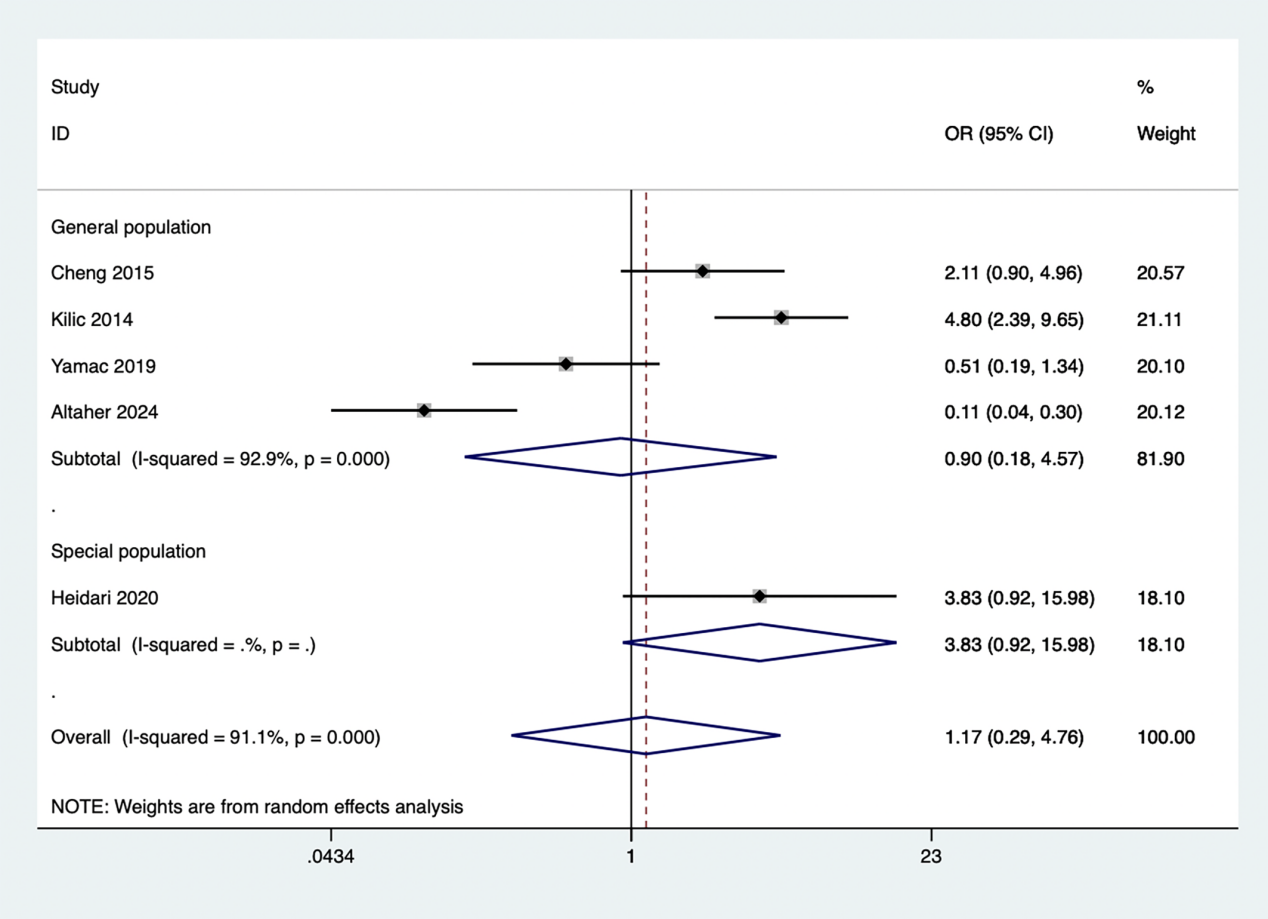


**Disease subgroup analysis: MI for rs7069102 under the homozygote model (GG vs CC).**


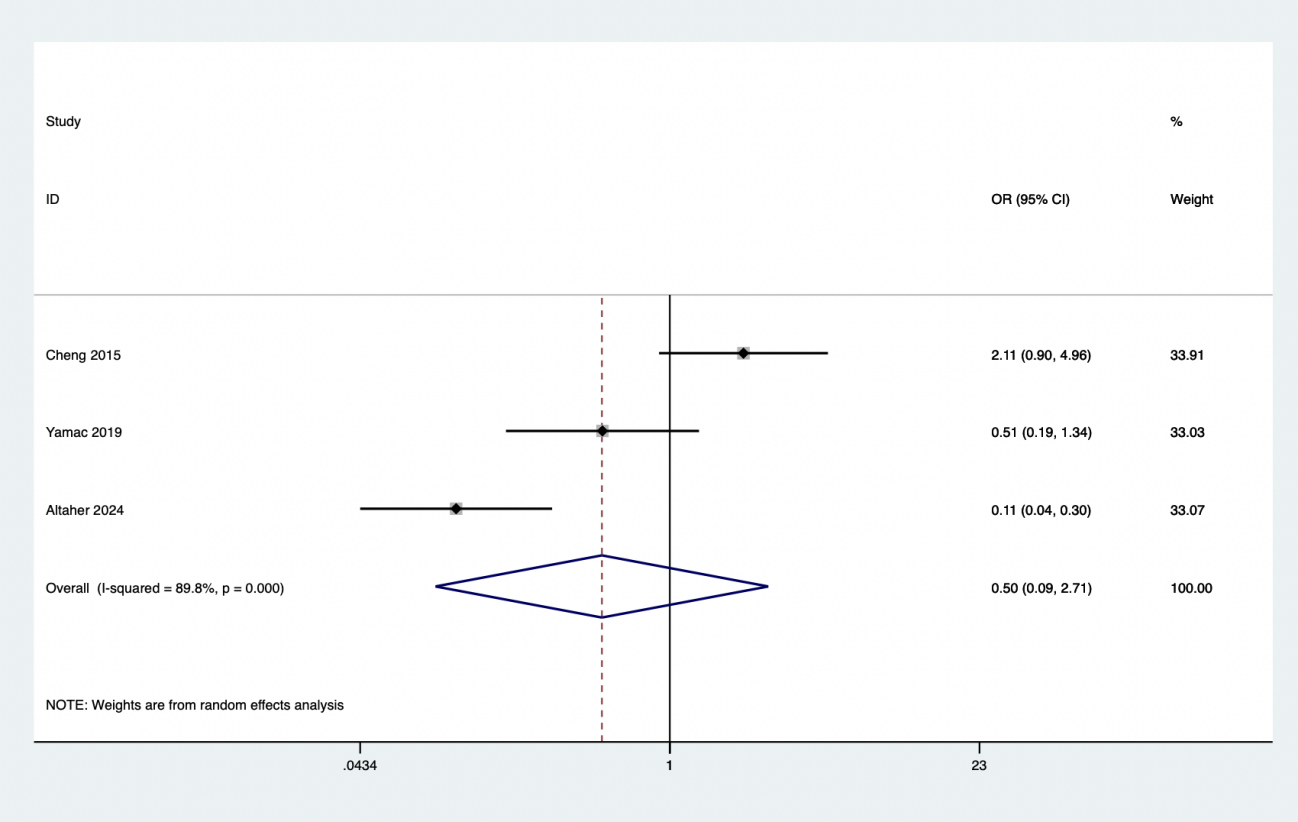


**Disease subgroup analysis: CAD for rs7069102 under the homozygote model (GG vs CC).**


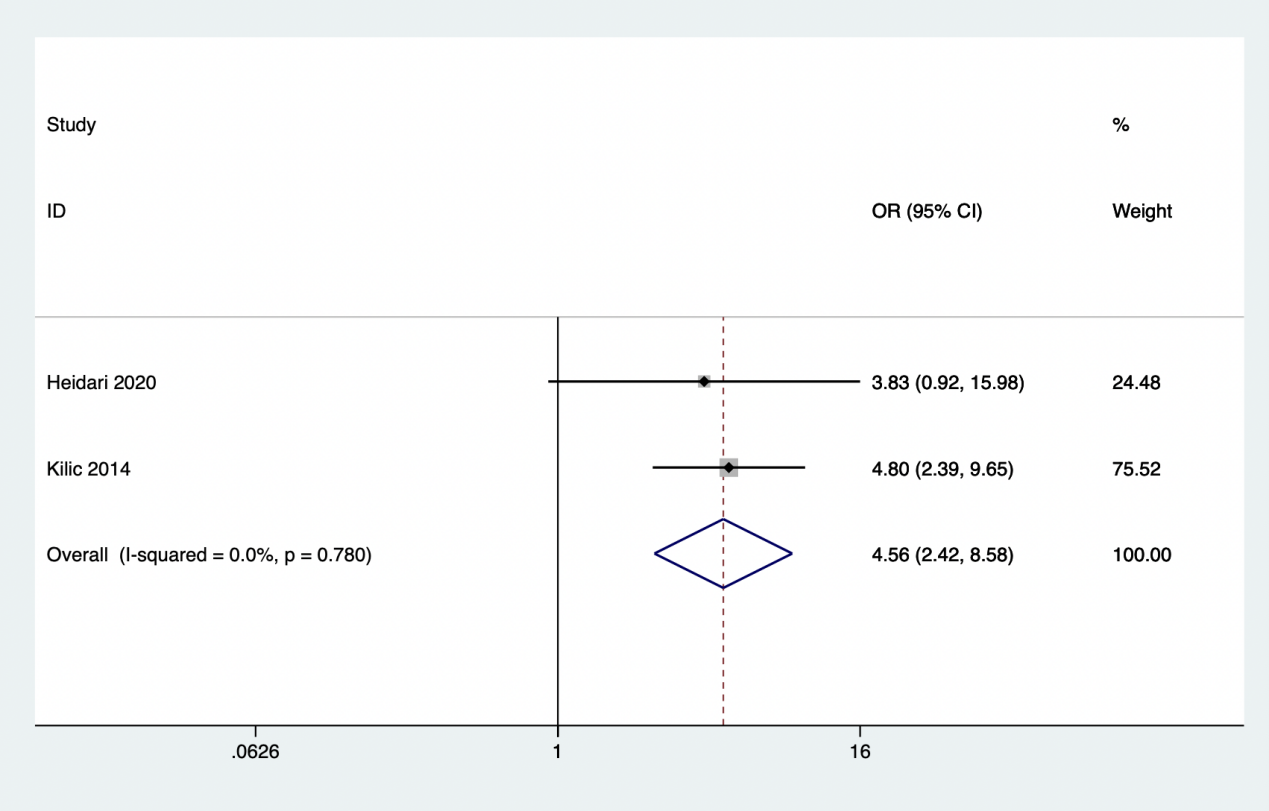


**Ethnicity subgroup analysis for rs7069102 under the homozygote model (GG vs CC).**


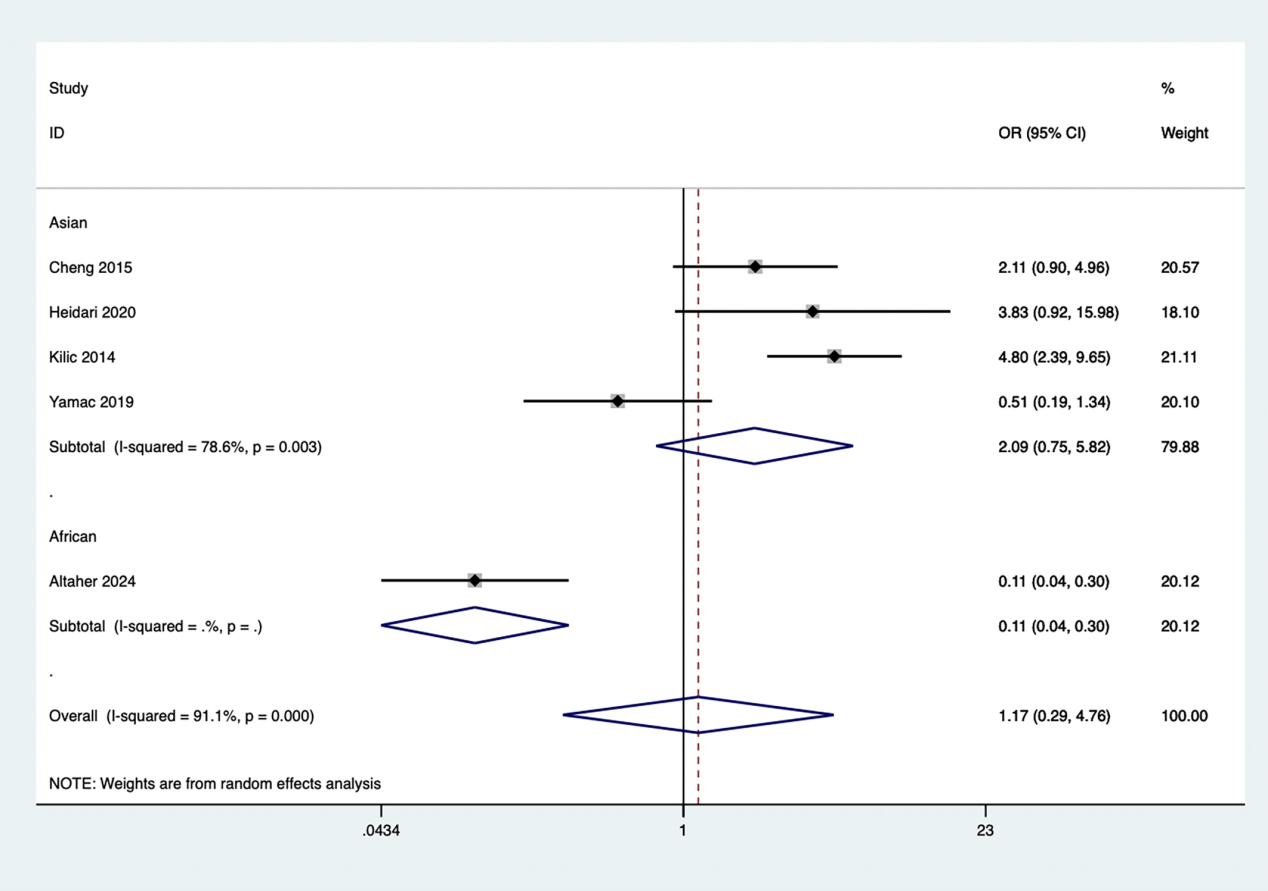


**Study design subgroup analysis for rs7069102 under the homozygote model (GG vs CC).**


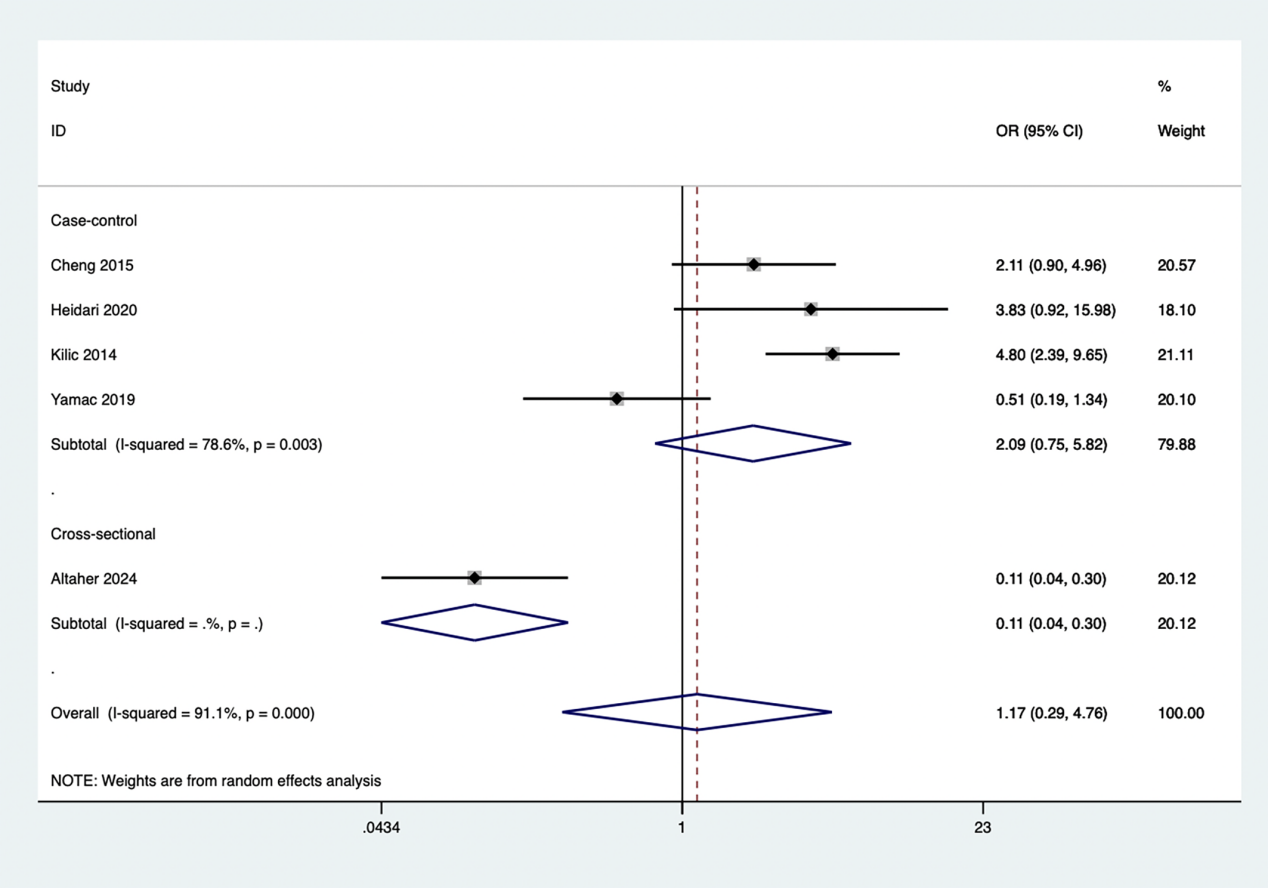


**Heterozygote model (CG vs CC)**

**Overall meta-analysis for rs7069102 under the heterozygote model (CG vs CC).**


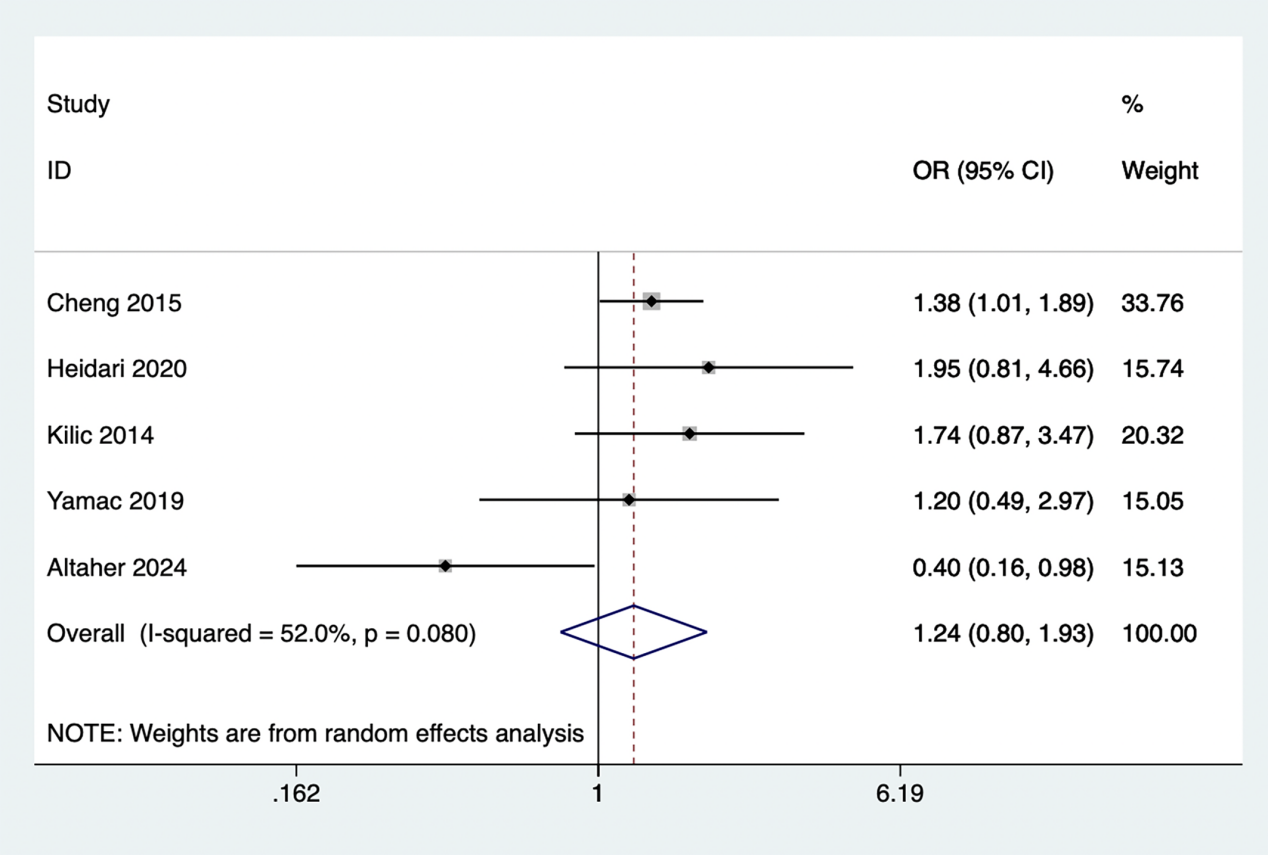


**Sensitivity analysis for rs7069102 under the heterozygote model (CG vs CC).**


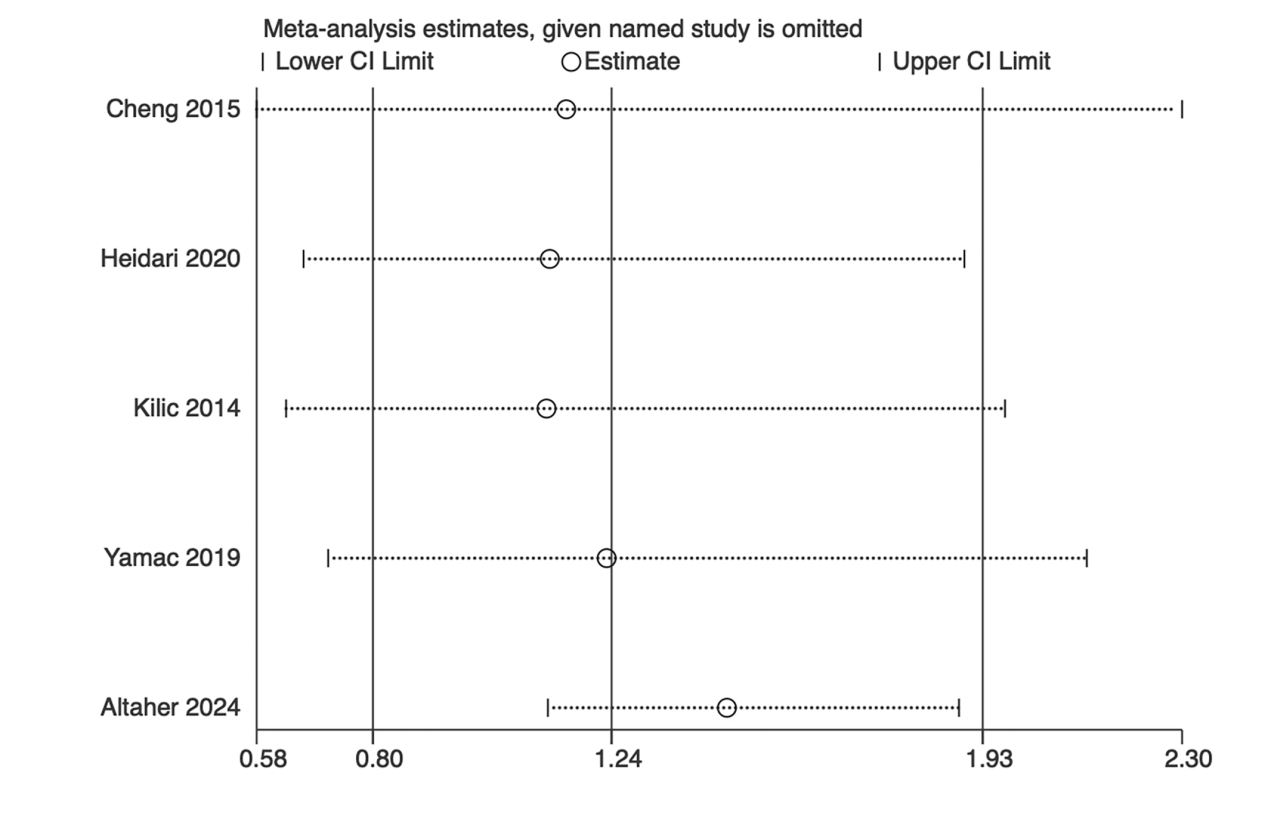


**Population subgroup analysis for rs7069102 under the heterozygote model (CG vs CC).**


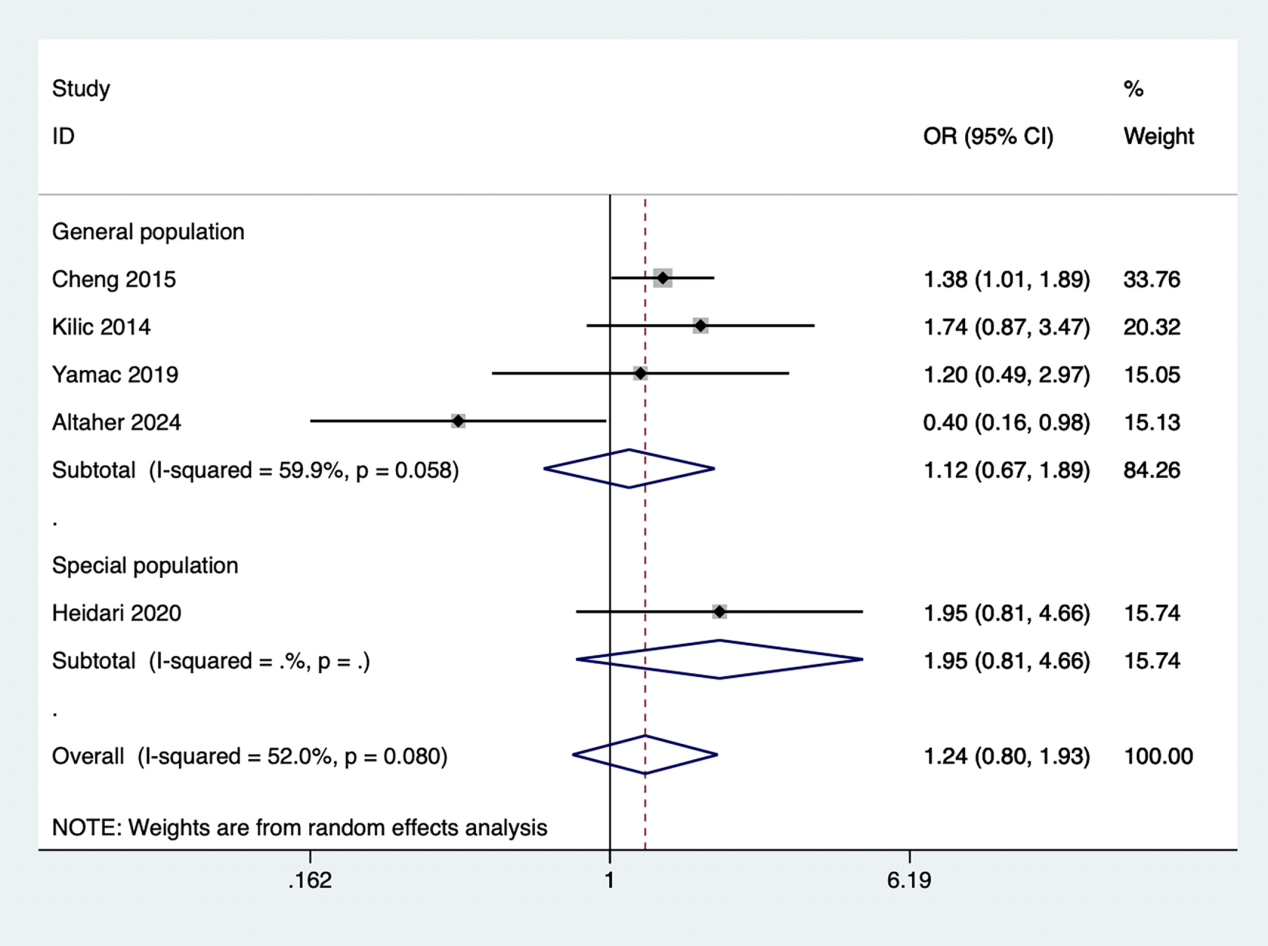


**Disease subgroup analysis: MI for rs7069102 under the heterozygote model (CG vs CC).**


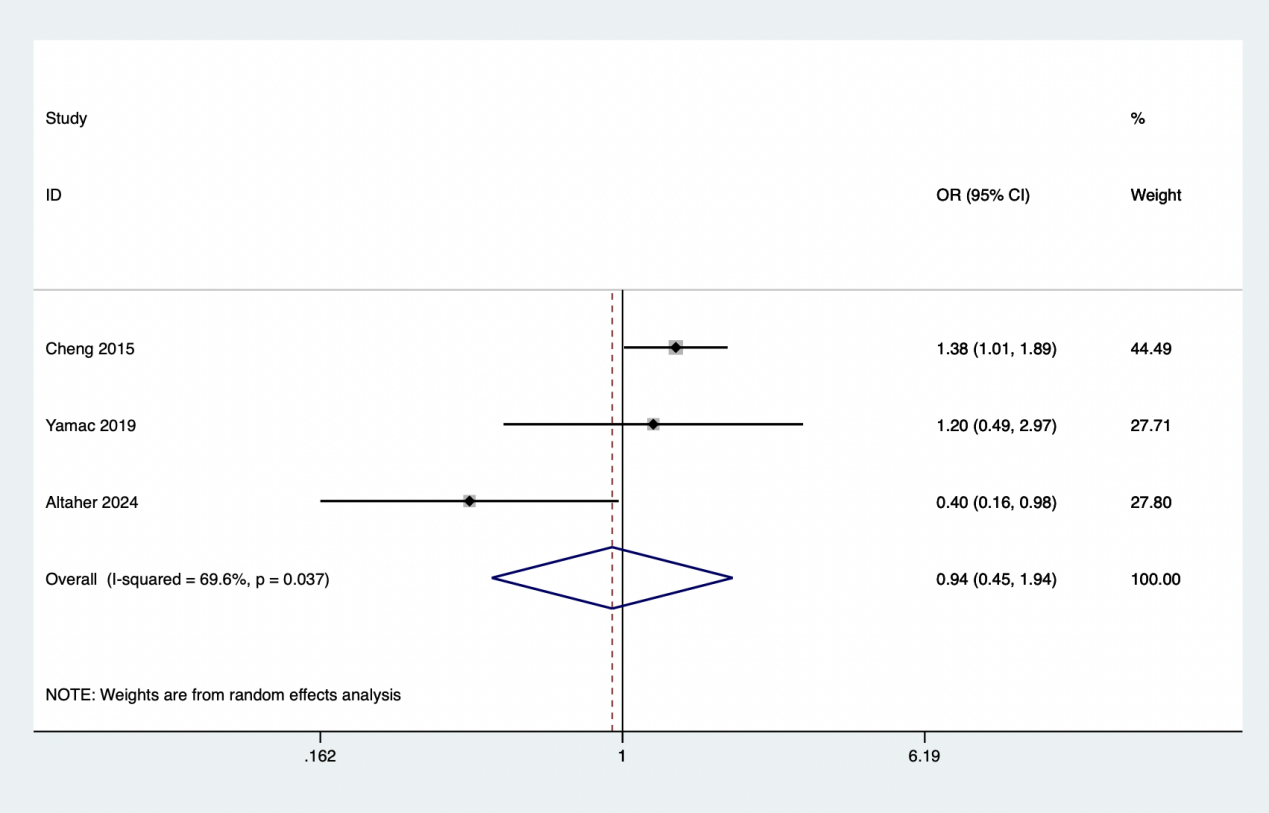


**Disease subgroup analysis: CAD for rs7069102 under the heterozygote model (CG vs CC).**


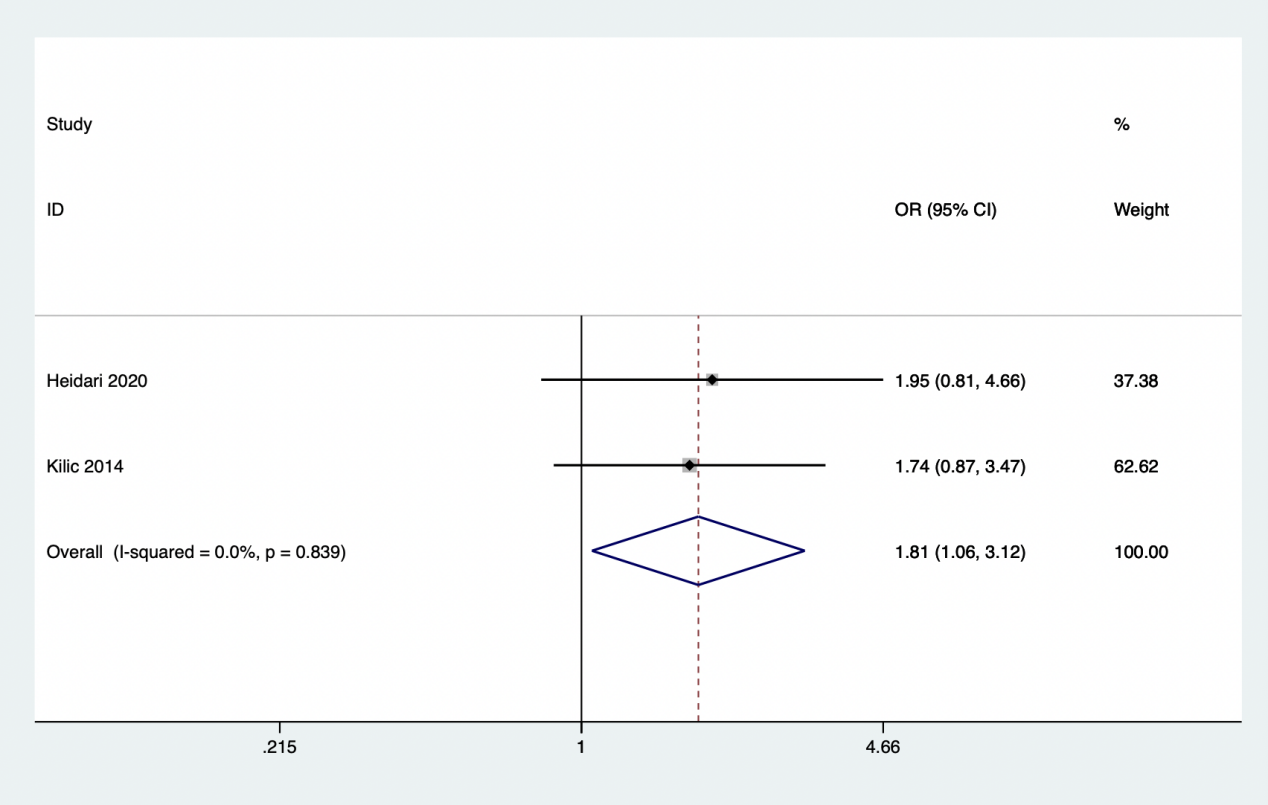


**Ethnicity subgroup analysis: Asian population for rs7069102 under the heterozygote model (CG vs CC).**


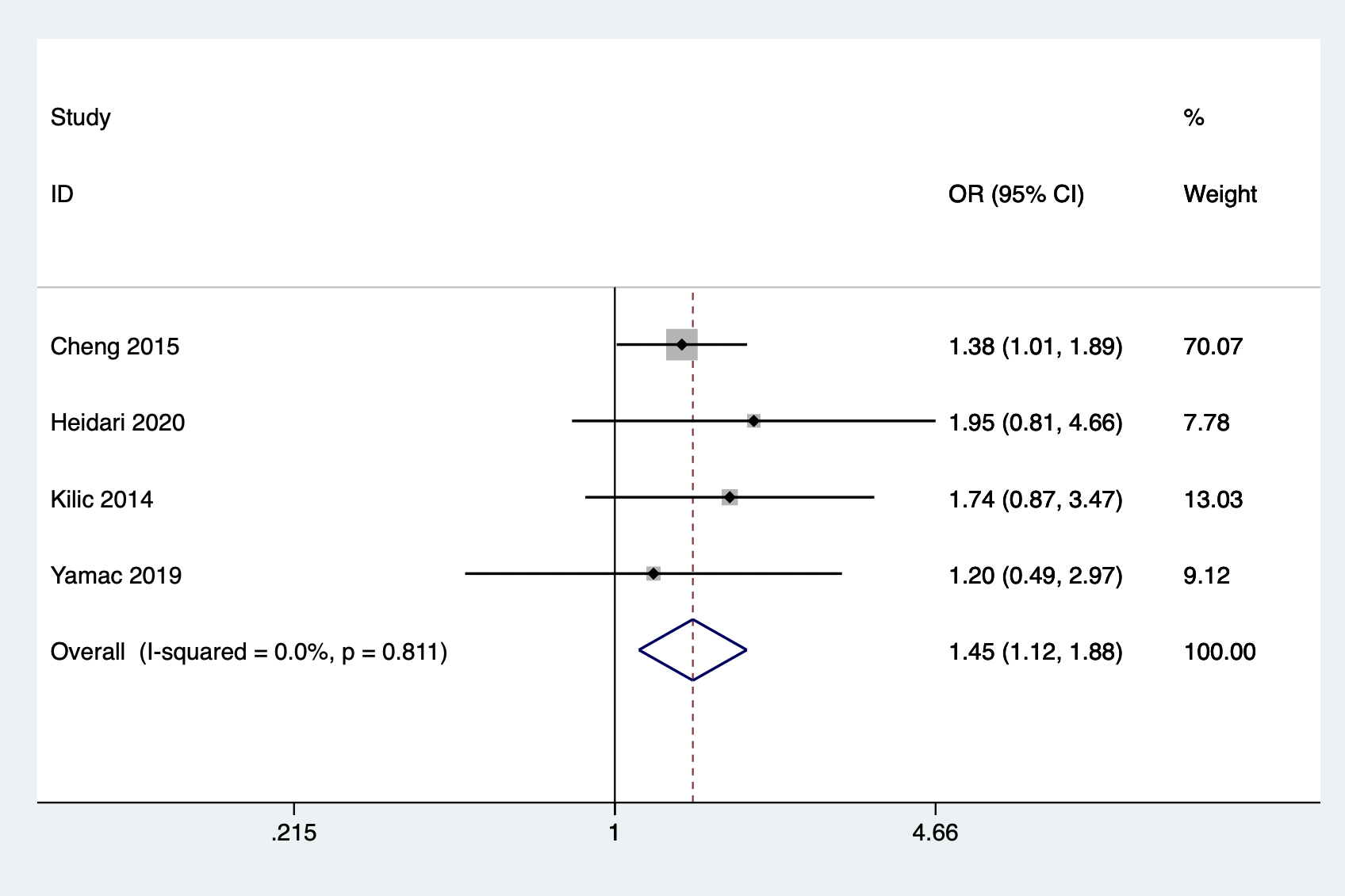


**Ethnicity subgroup analysis: African population for rs7069102 under the heterozygote model (CG vs CC).**


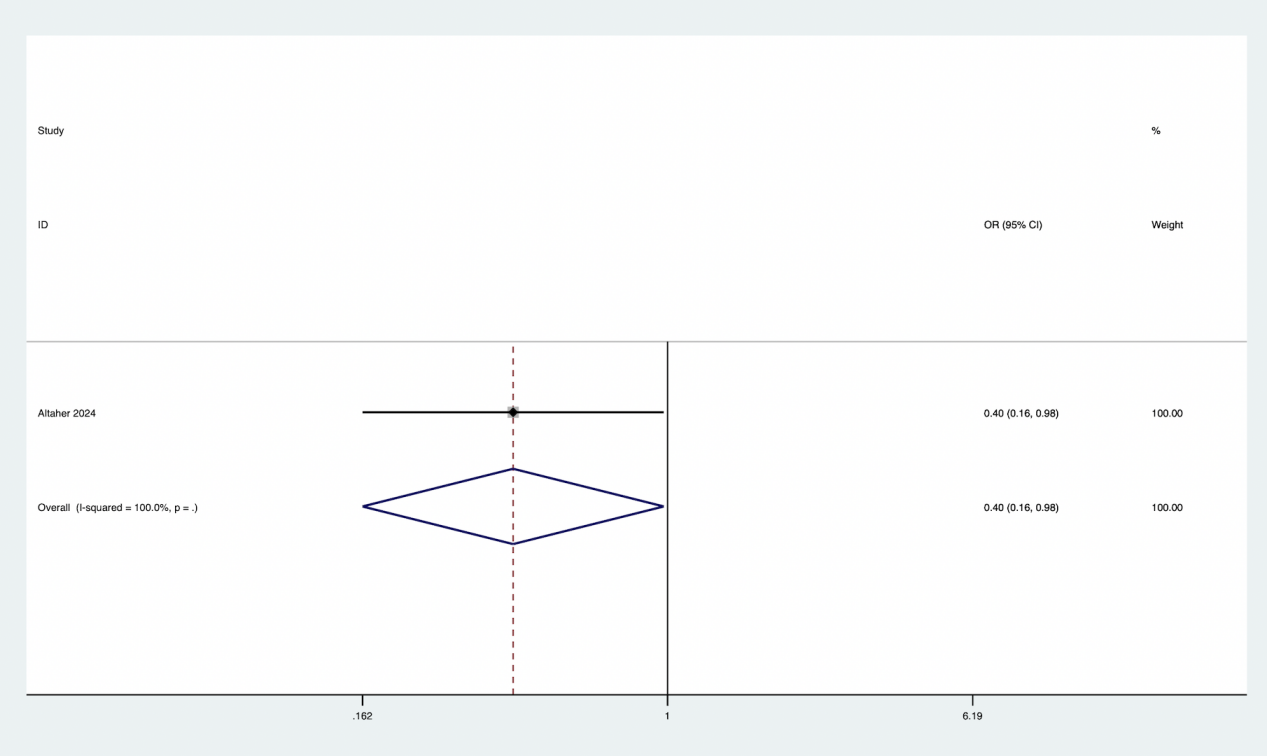


**Study design subgroup analysis: case-control studies for rs7069102 under the heterozygote model (CG vs CC).**


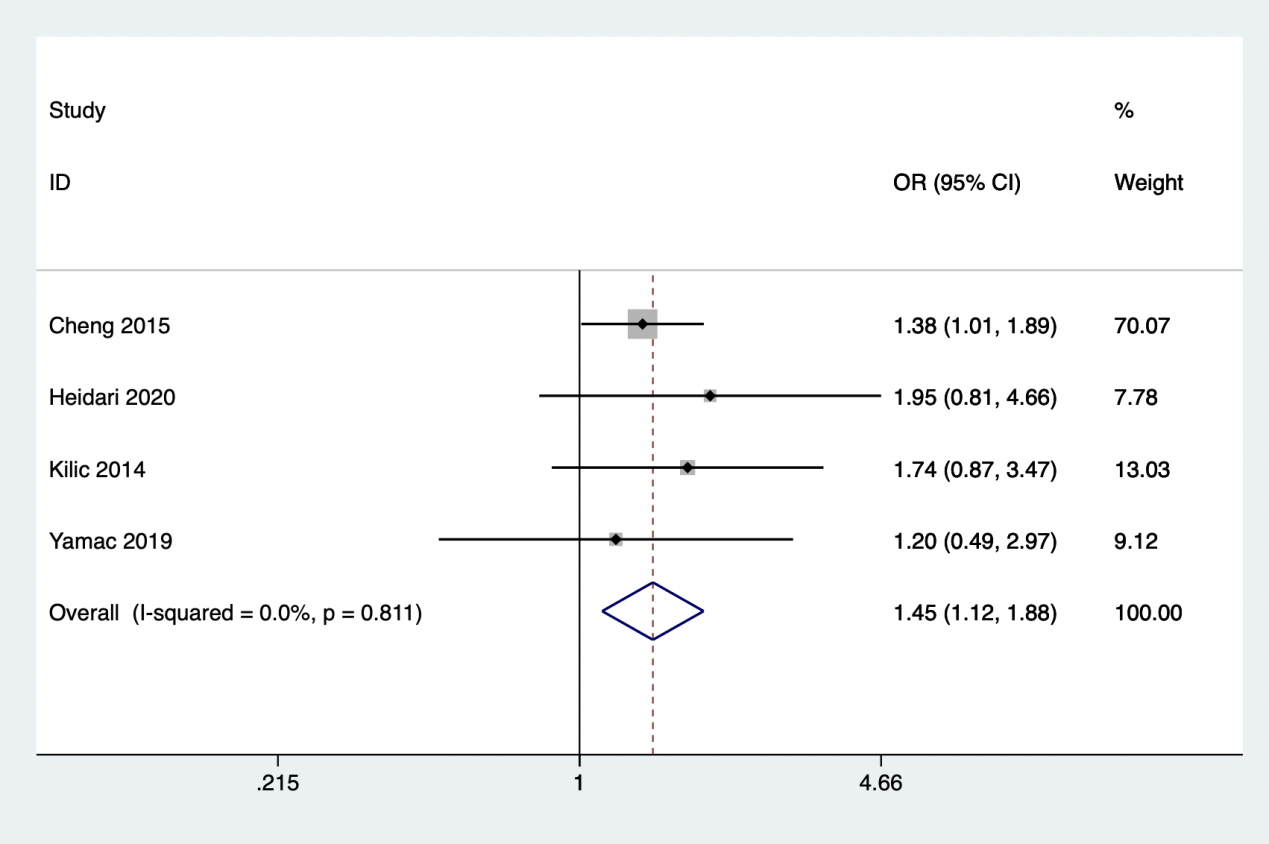


**Study design subgroup analysis: cross-sectional study for rs7069102 under the heterozygote model (CG vs CC).**


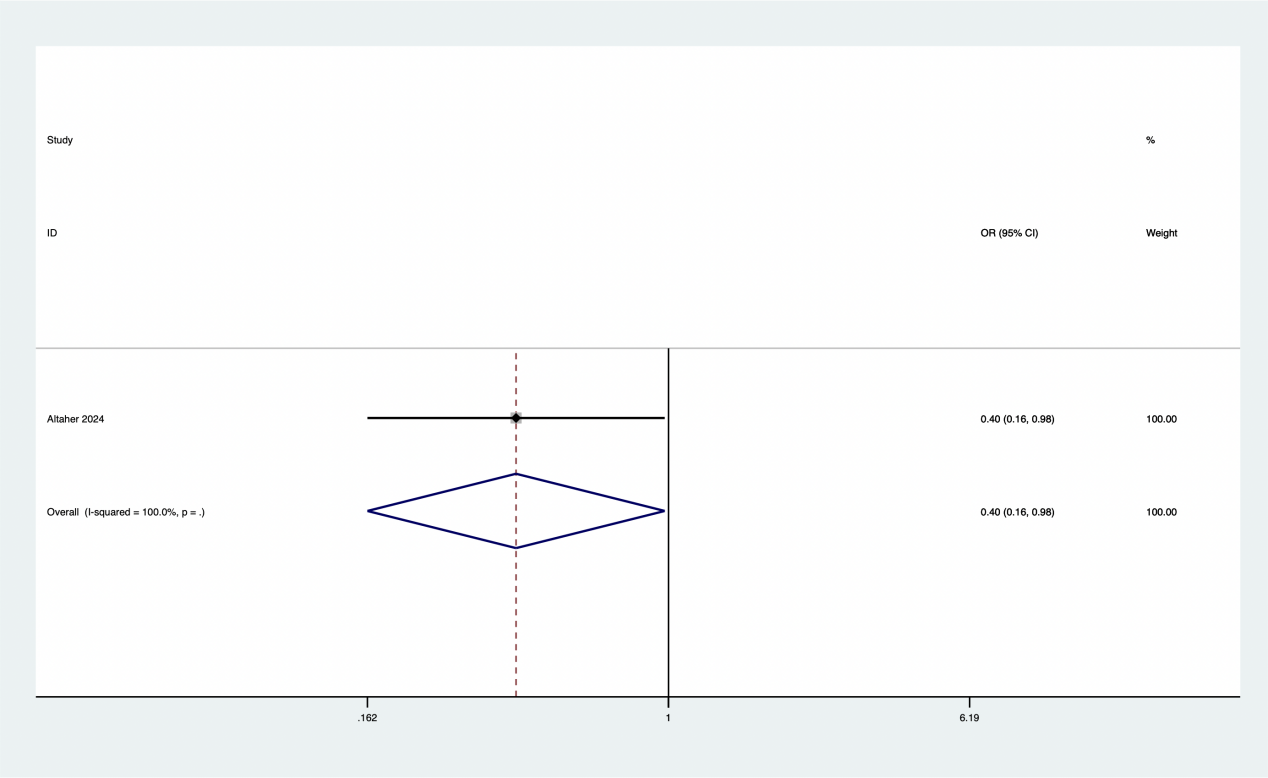

Supplement: Supplementary file 3 [file Table3.docx]
