# Supplementary material for: Association between SIRT1 gene polymorphisms and susceptibility to coronary artery disease: a systematic review and meta-analysis
Source: Front Cardiovasc Med. 2026 Jul 3;13:1850297. doi: 10.3389/fcvm.2026.1850297 (PMC13376308; doi:10.3389/fcvm.2026.1850297)

**Supplementary Material 4. Meta-analysis results for rs7895833 (Exp = G)**

**Allelic model (G vs A)**

**Overall meta-analysis for rs7895833 under the allelic model (G vs A).**


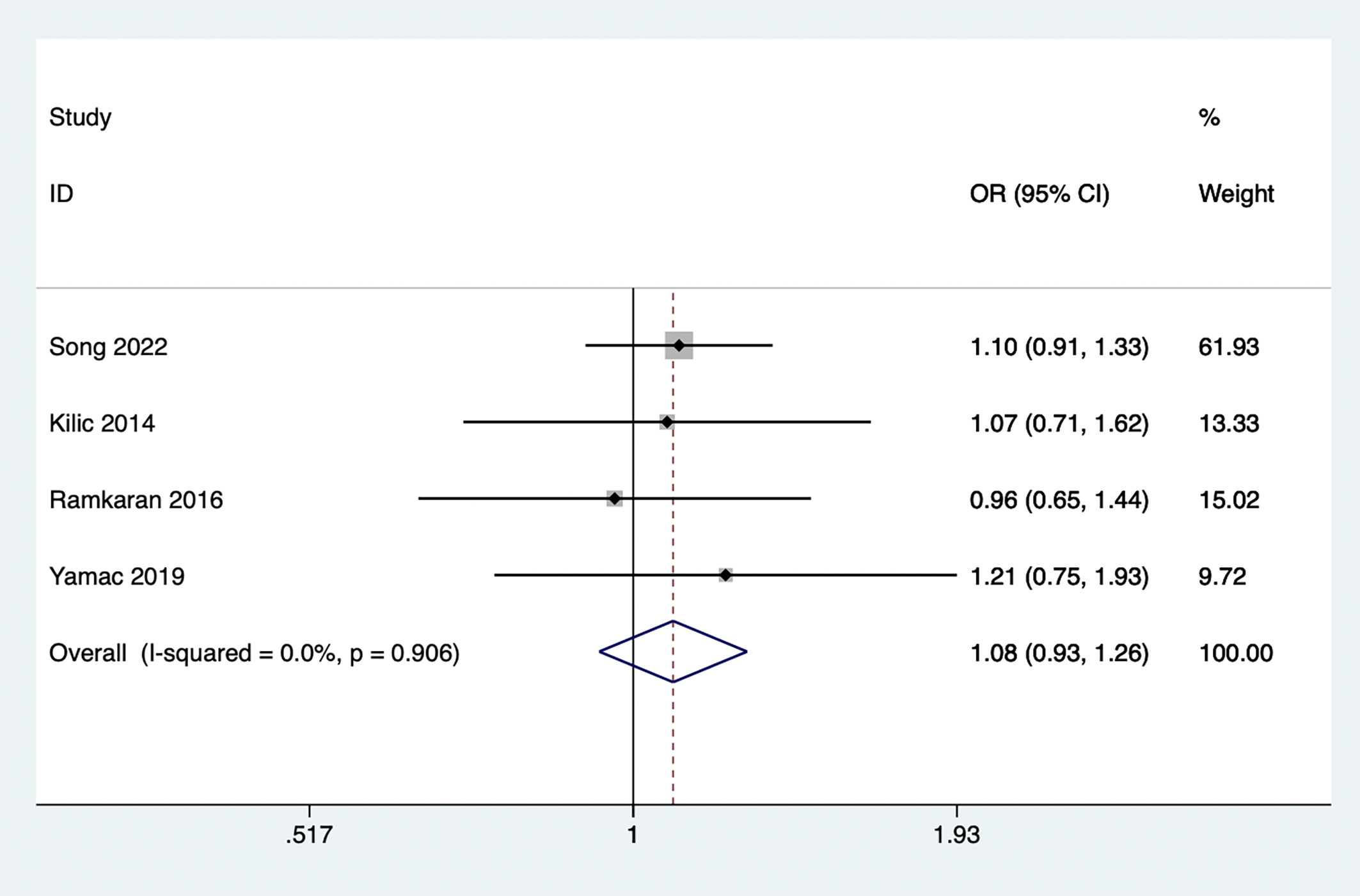


**Disease subgroup analysis for rs7895833 under the allelic model (G vs A).**


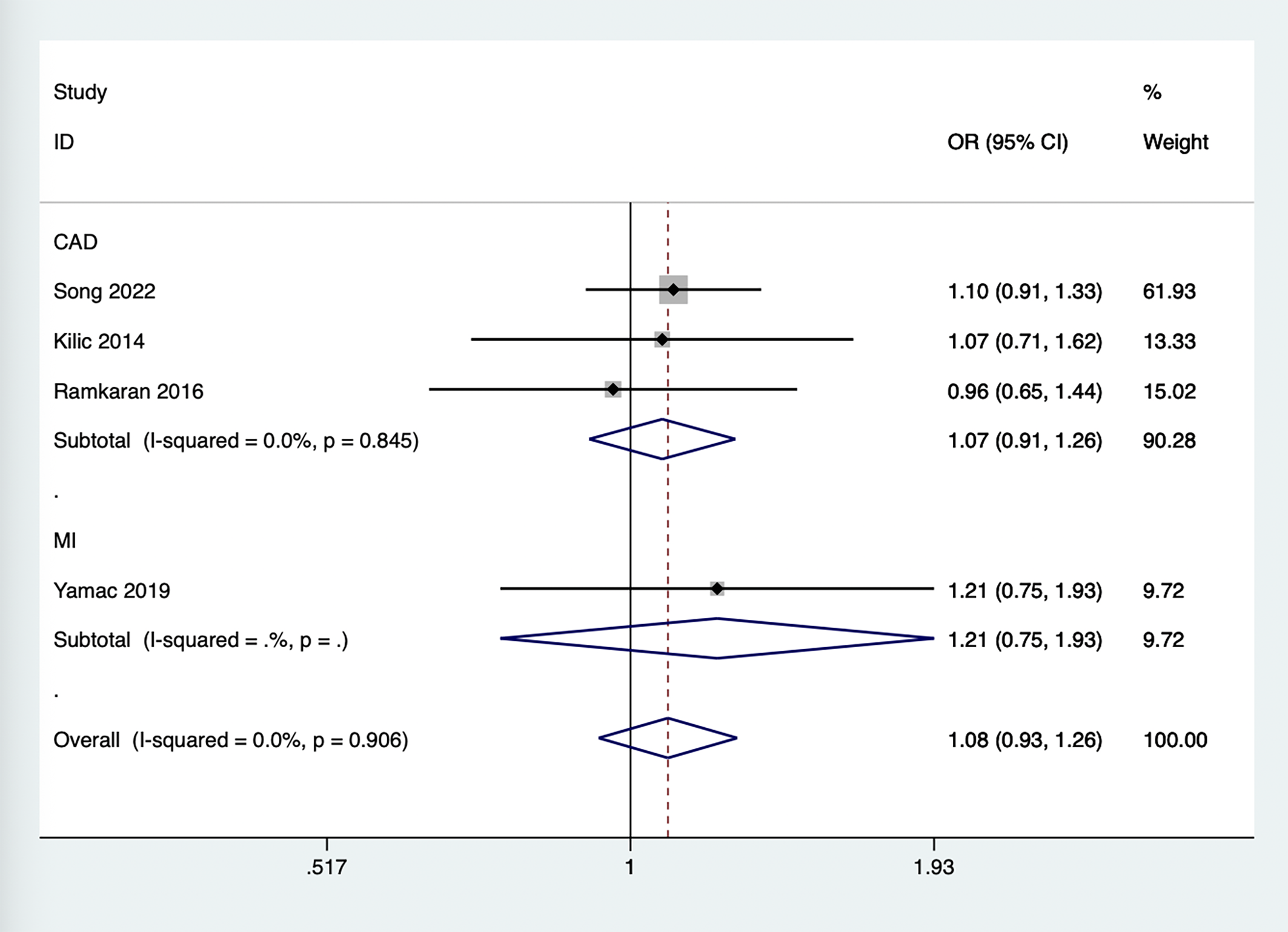


**Ethnicity subgroup analysis for rs7895833 under the allelic model (G vs A).**


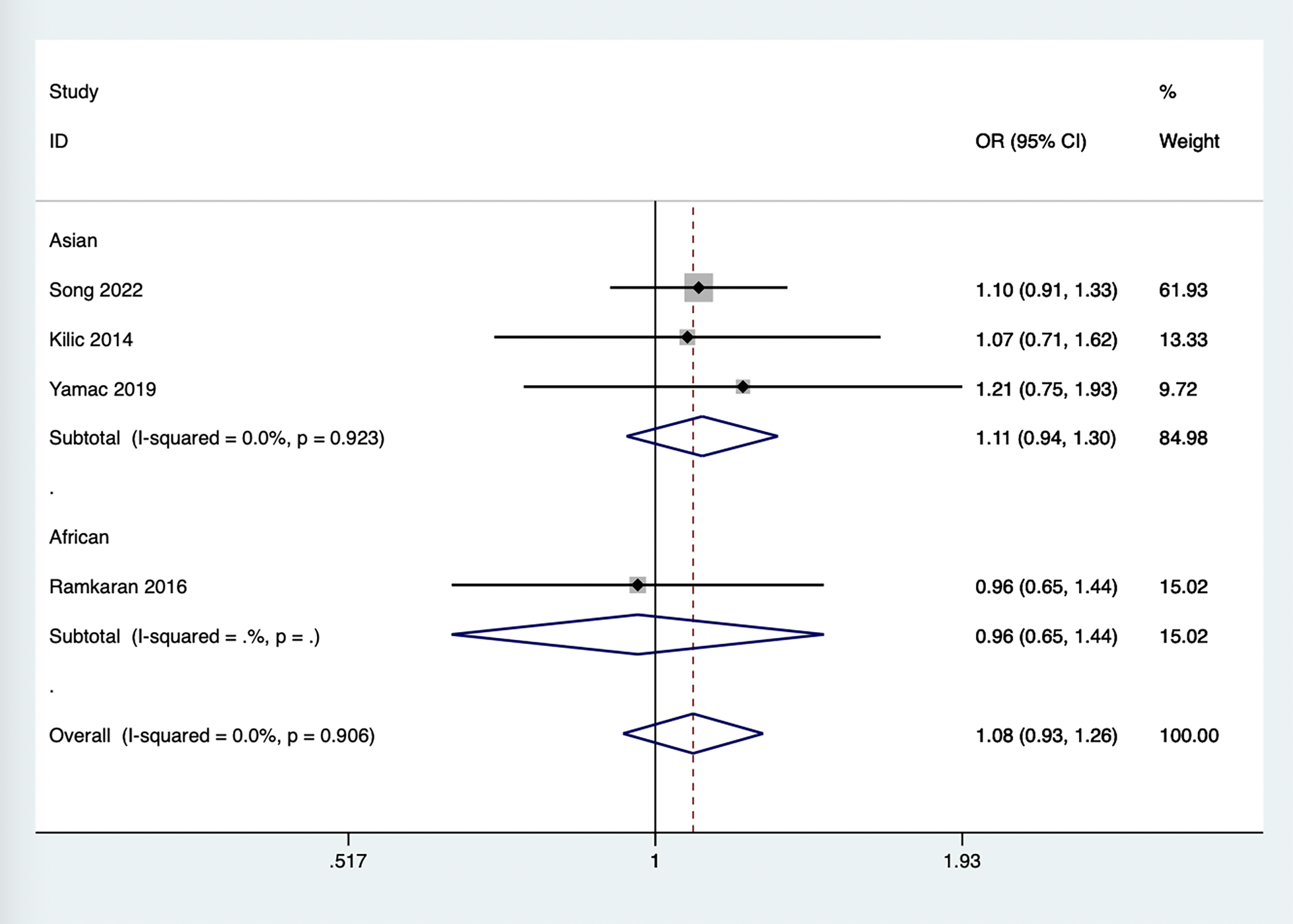


**Dominant model (GG+AG vs AA)**

**Overall meta-analysis for rs7895833 under the dominant model (GG+AG vs AA).**


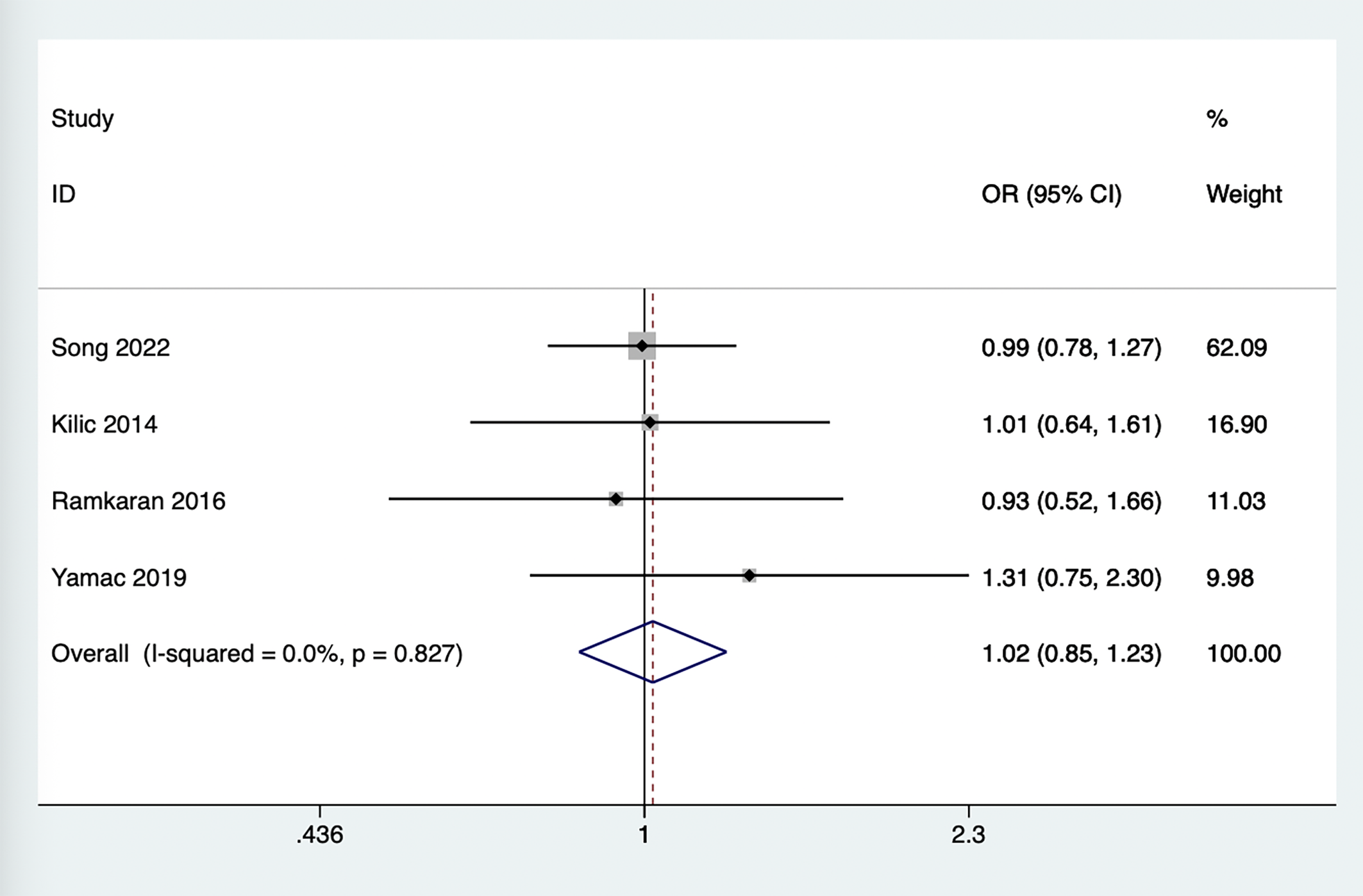


**Disease subgroup analysis for rs7895833 under the dominant model (GG+AG vs AA).**


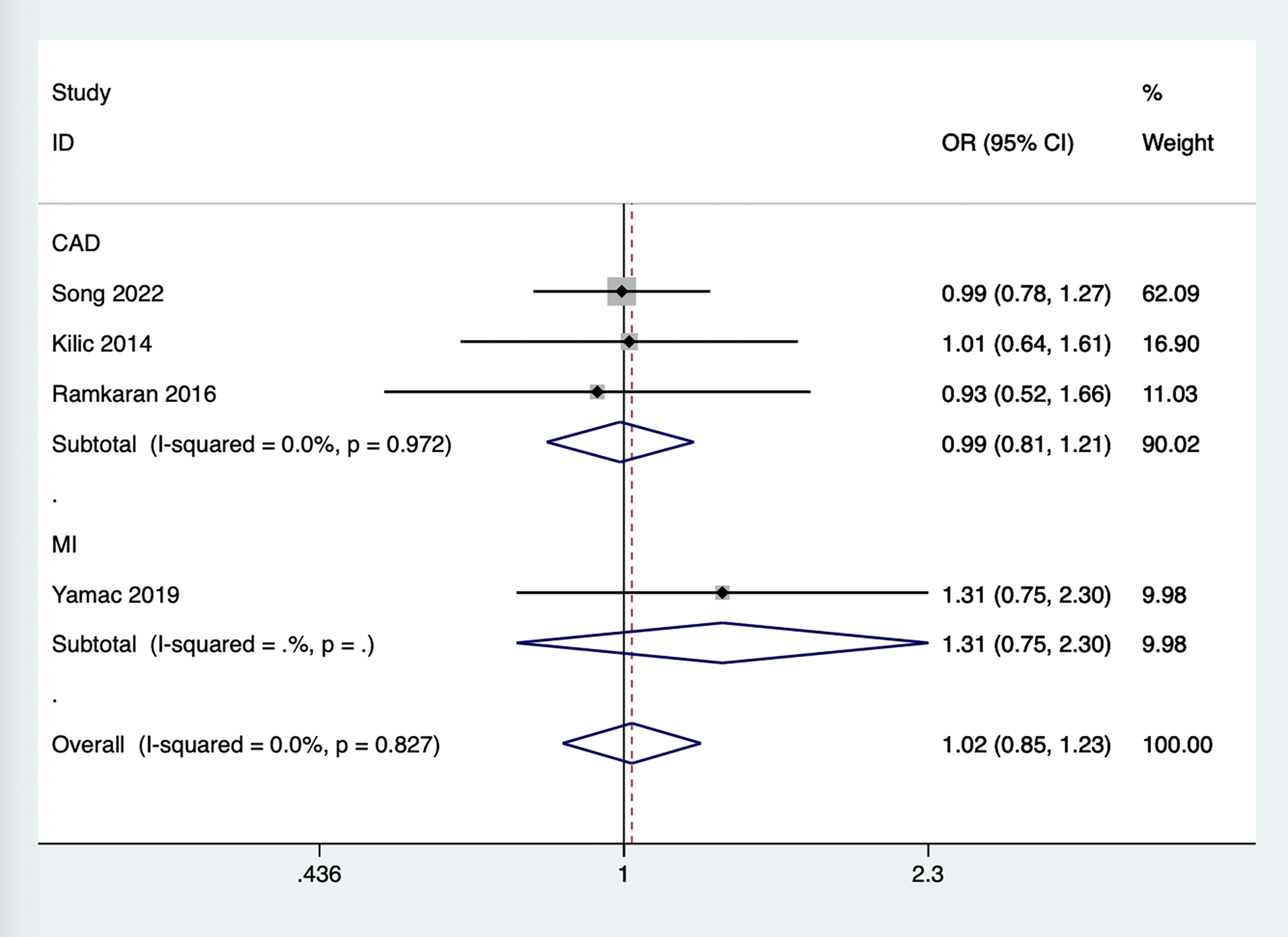


**Ethnicity subgroup analysis for rs7895833 under the dominant model (GG+AG vs AA).**


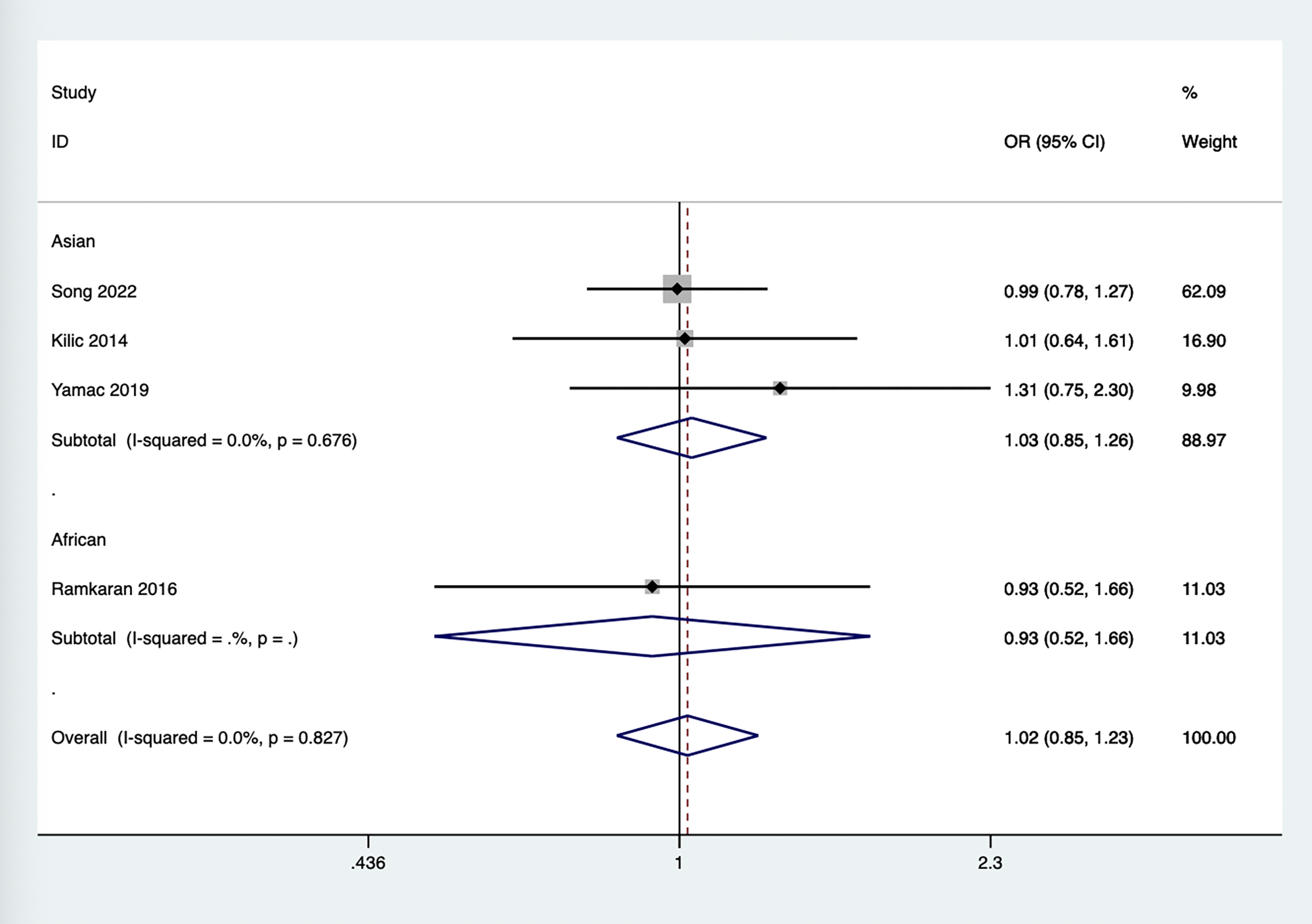


**Recessive model (GG vs AG+AA)**

**Overall meta-analysis for rs7895833 under the recessive model (GG vs AG+AA).**


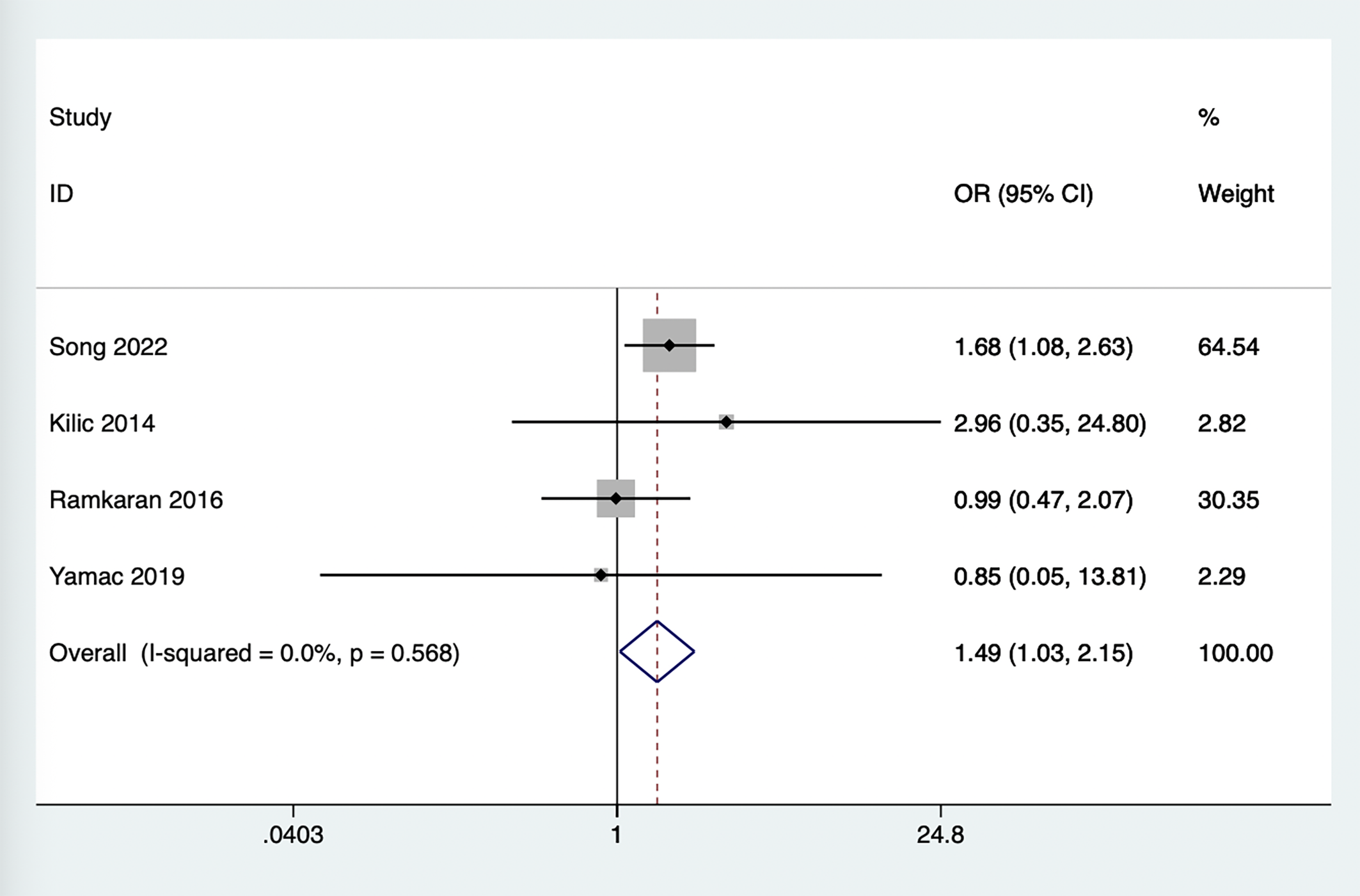


**Disease subgroup analysis for rs7895833 under the recessive model (GG vs AG+AA).**


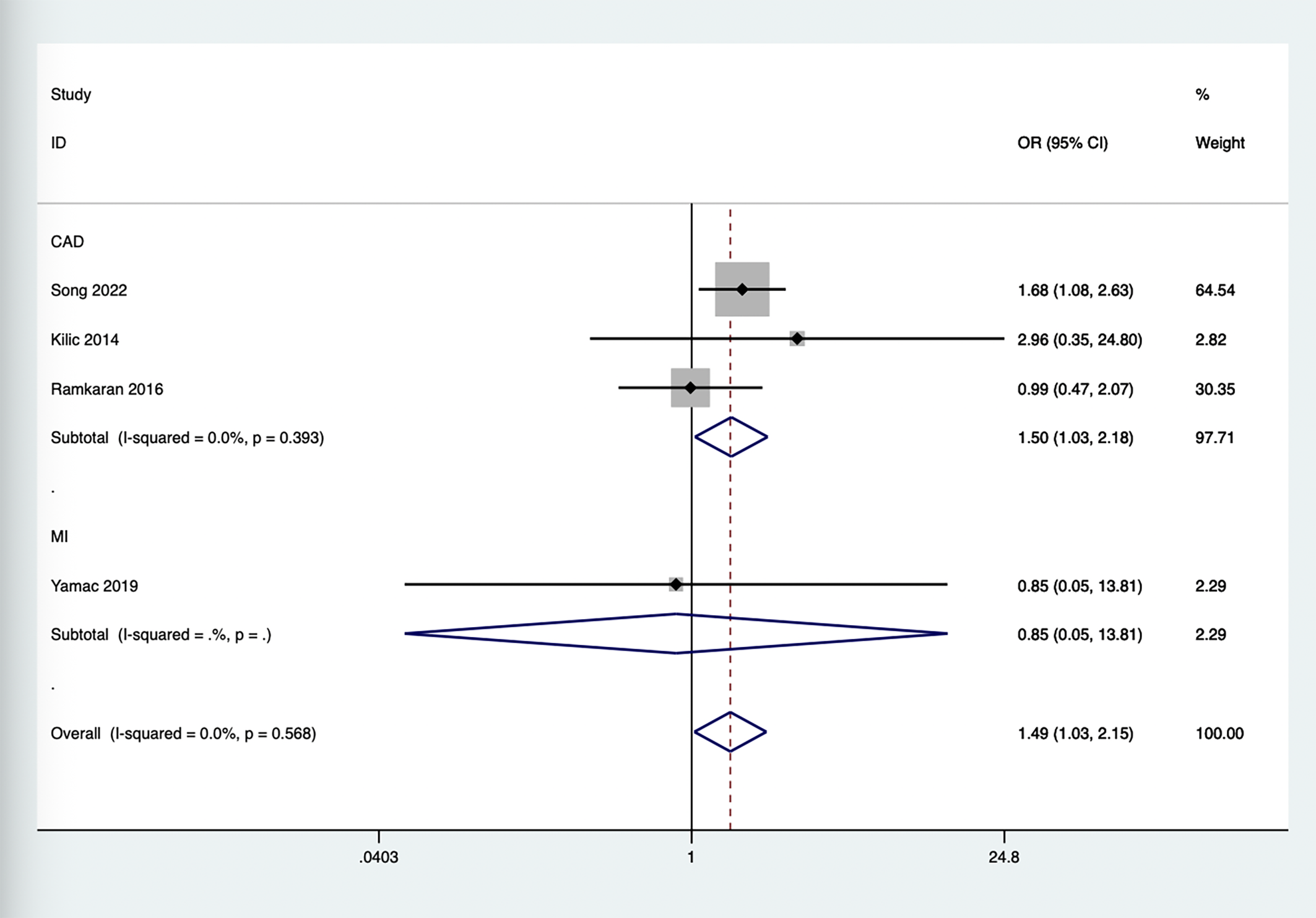


**Ethnicity subgroup analysis for rs7895833 under the recessive model (GG vs AG+AA).**


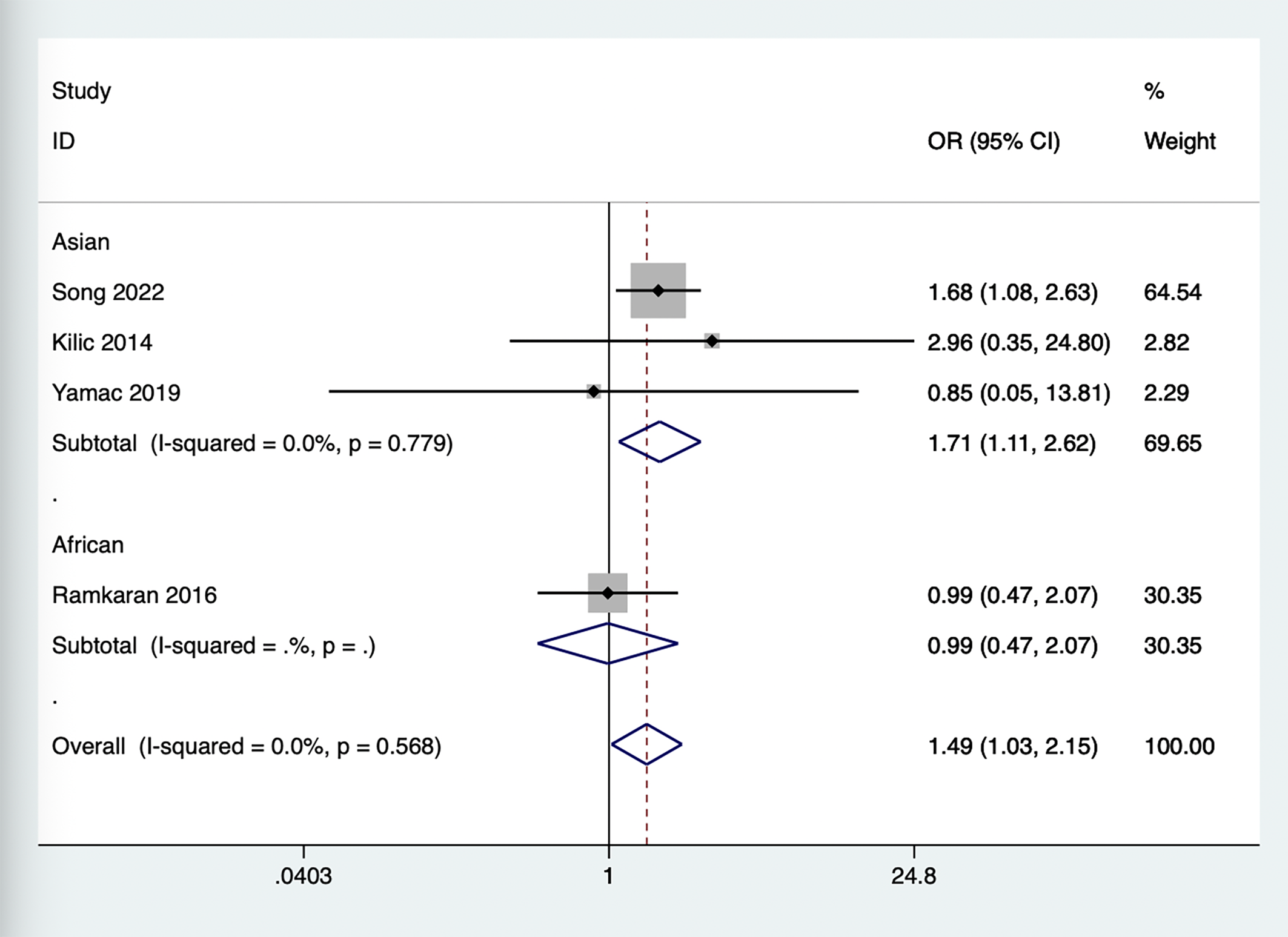


**Homozygote model (GG vs AA)**

**Overall meta-analysis for rs7895833 under the homozygote model (GG vs AA).**


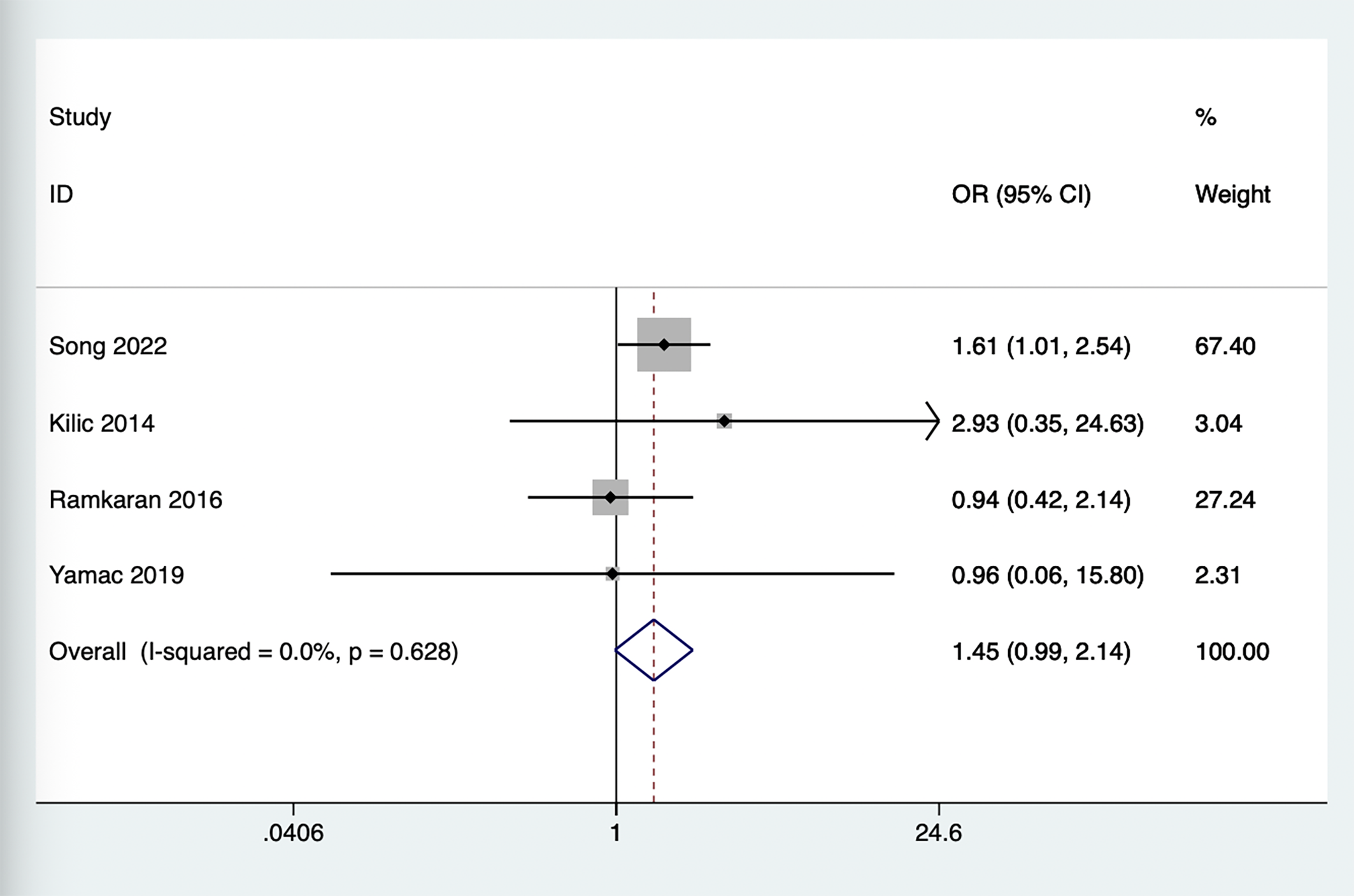


**Disease subgroup analysis for rs7895833 under the homozygote model (GG vs AA).**


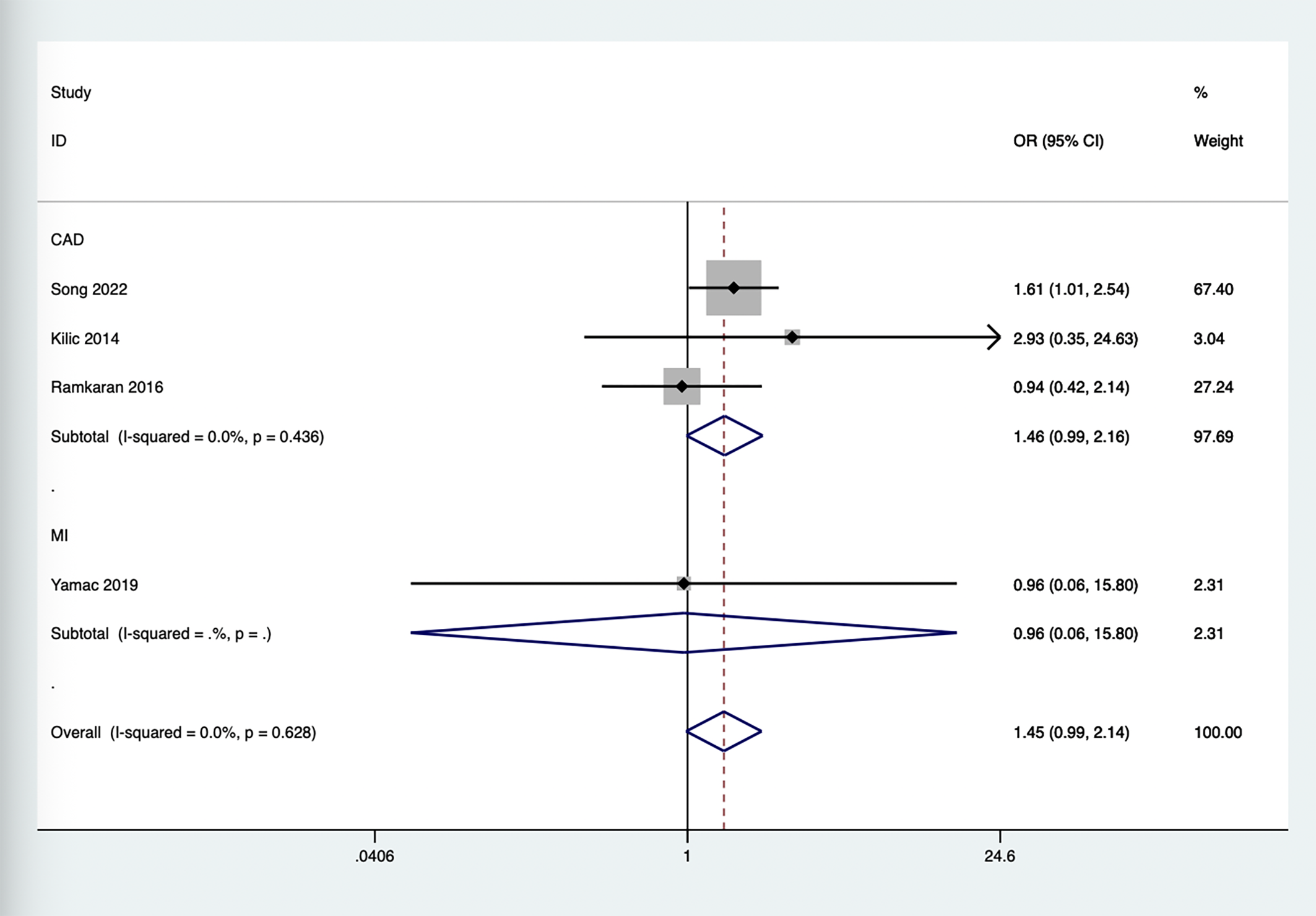


**Ethnicity subgroup analysis for rs7895833 under the homozygote model (GG vs AA).**


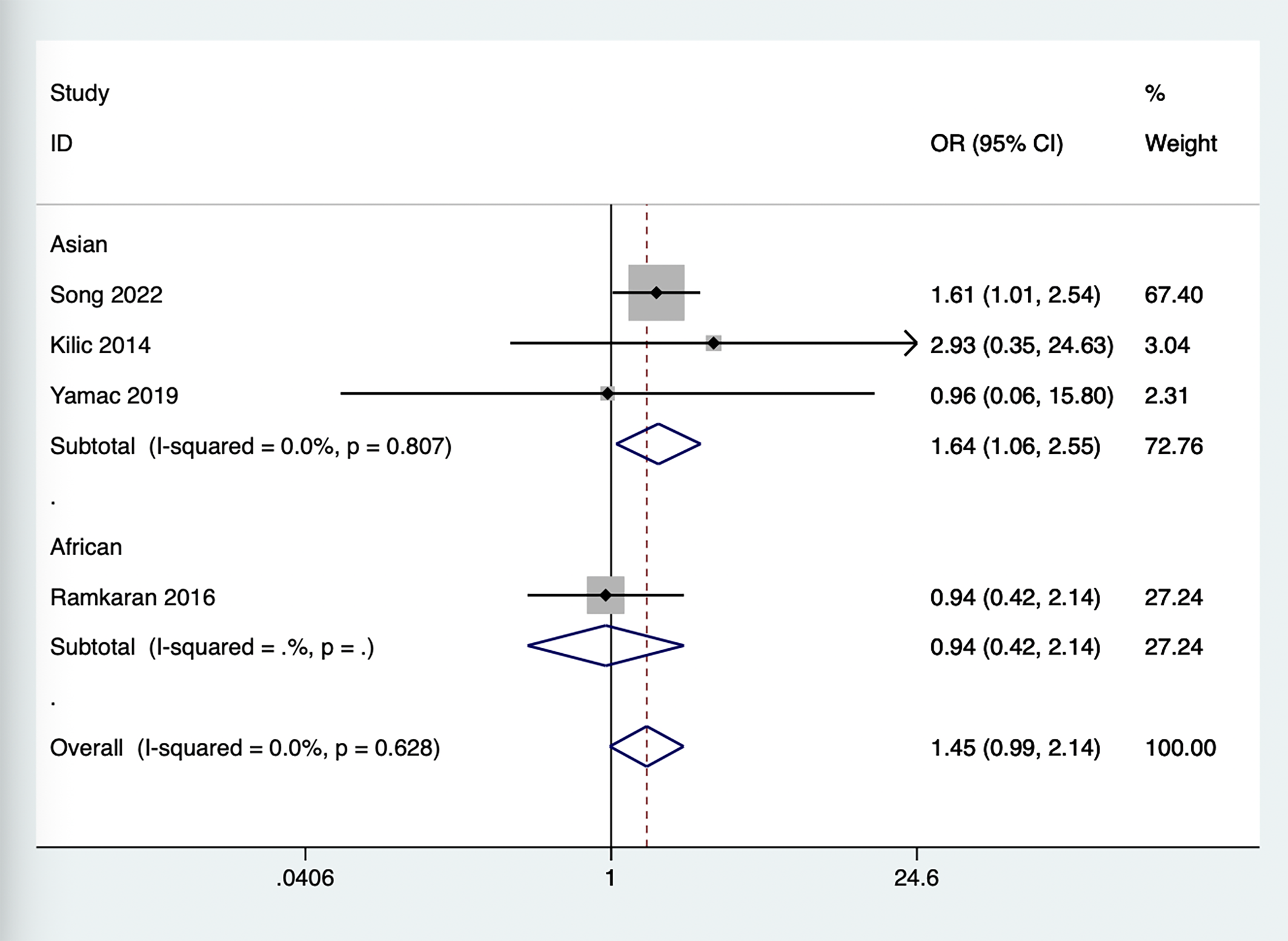


**Heterozygote model (AG vs AA)**

**Overall meta-analysis for rs7895833 under the heterozygote model (AG vs AA).**


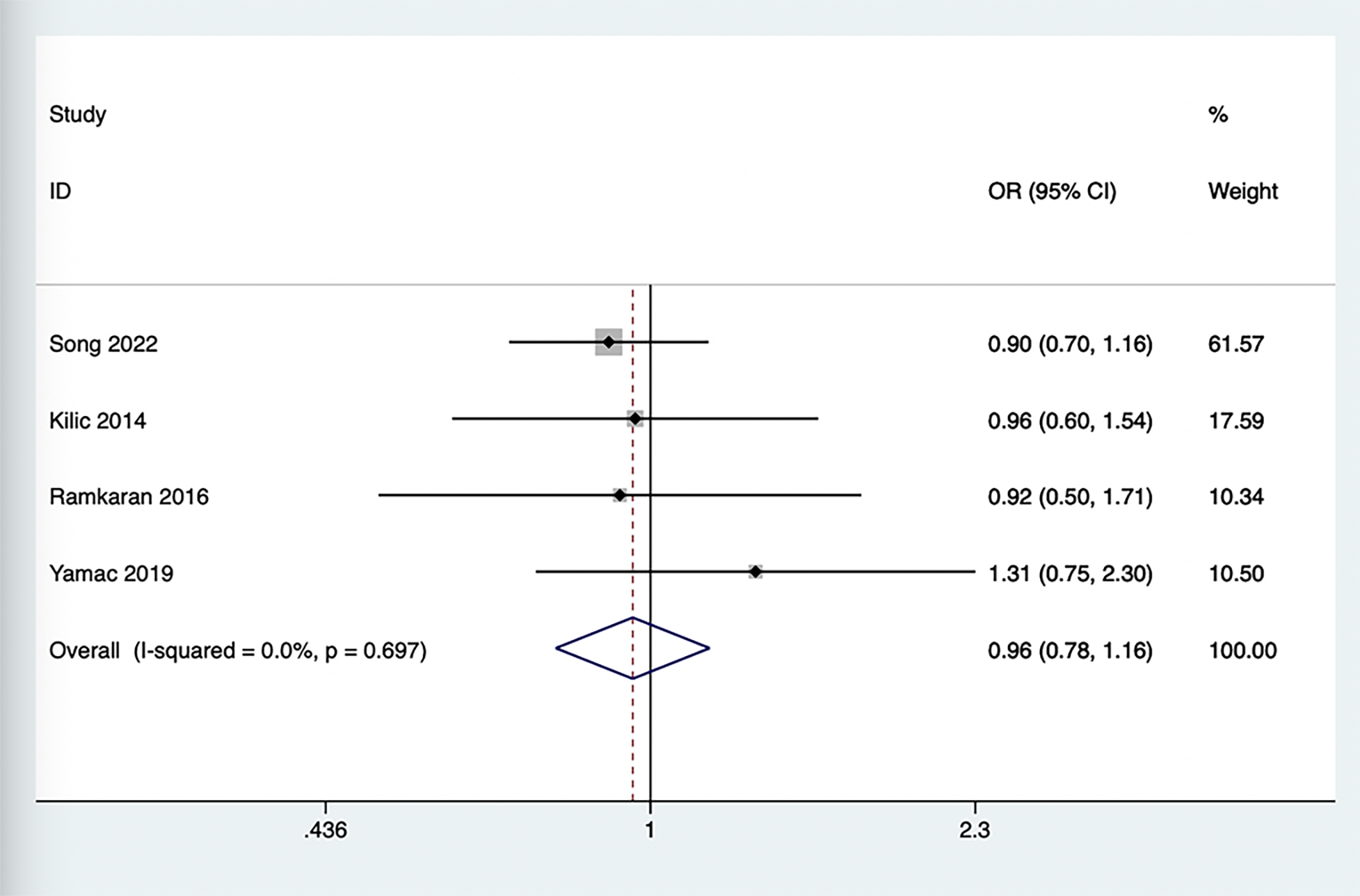


**Disease subgroup analysis for rs7895833 under the heterozygote model (AG vs AA).**


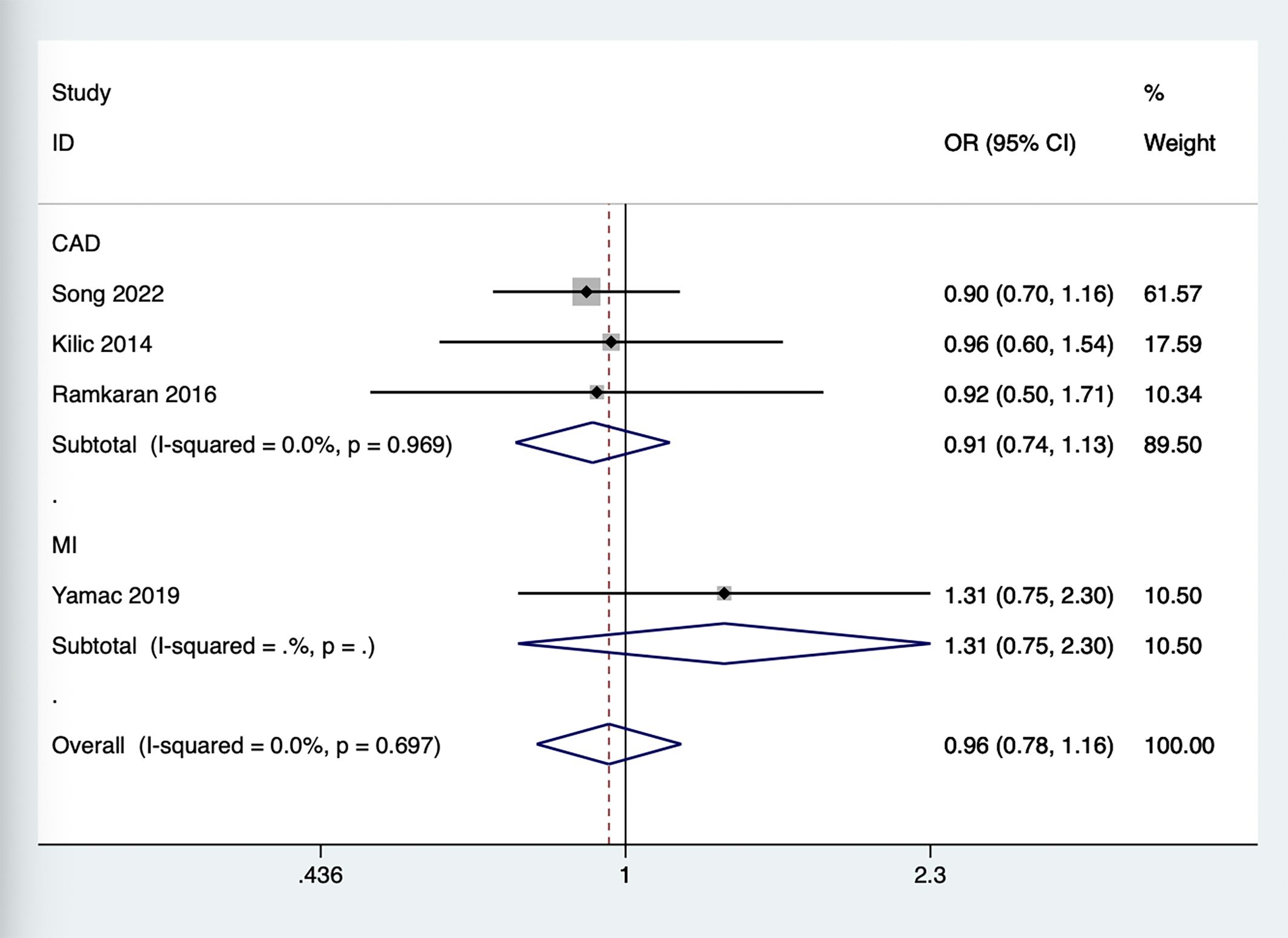


**Ethnicity subgroup analysis for rs7895833 under the heterozygote model (AG vs AA).**


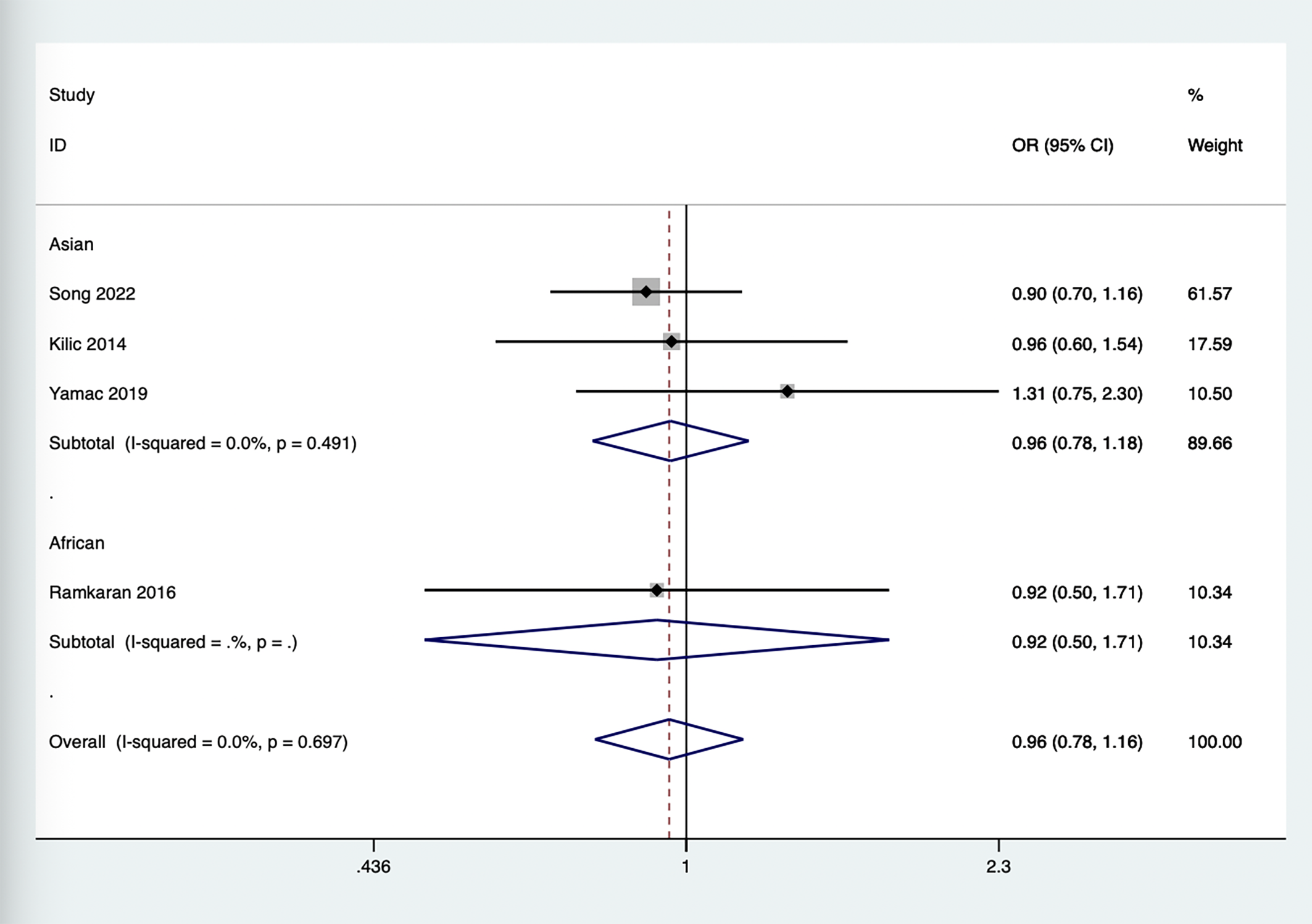

Supplement: Supplementary file 4 [file Table4.docx]
