# Supplementary material for: Association between SIRT1 gene polymorphisms and susceptibility to coronary artery disease: a systematic review and meta-analysis
Source: Front Cardiovasc Med. 2026 Jul 3;13:1850297. doi: 10.3389/fcvm.2026.1850297 (PMC13376308; doi:10.3389/fcvm.2026.1850297)

**Supplementary Material 5. Meta-analysis results for rs4746720 (Exp = T)**

**Allelic model (T vs C)**

**Overall meta-analysis for rs4746720 under the allelic model (T vs C).**


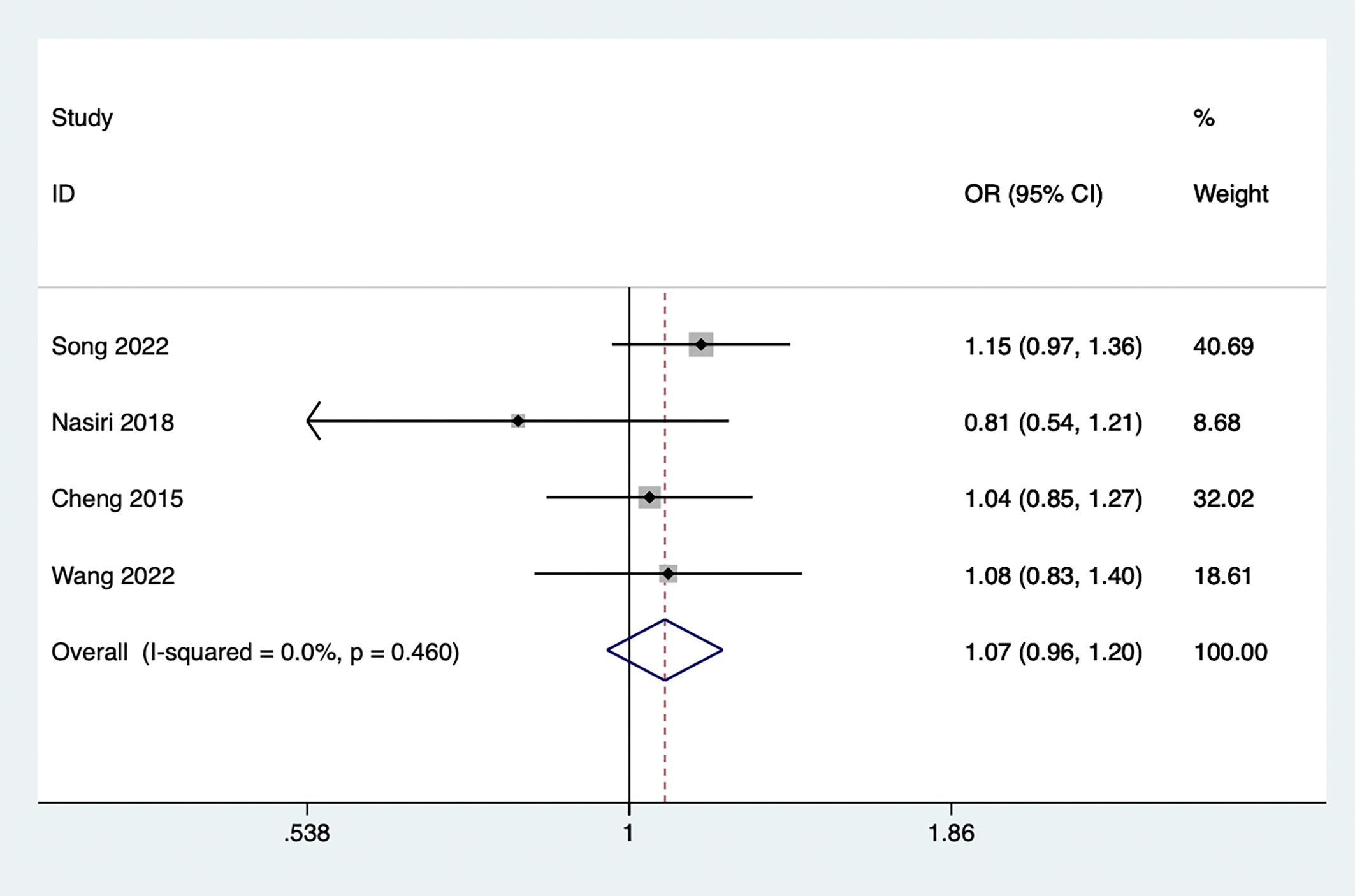


**Disease subgroup analysis for rs4746720 under the allelic model (T vs C).**


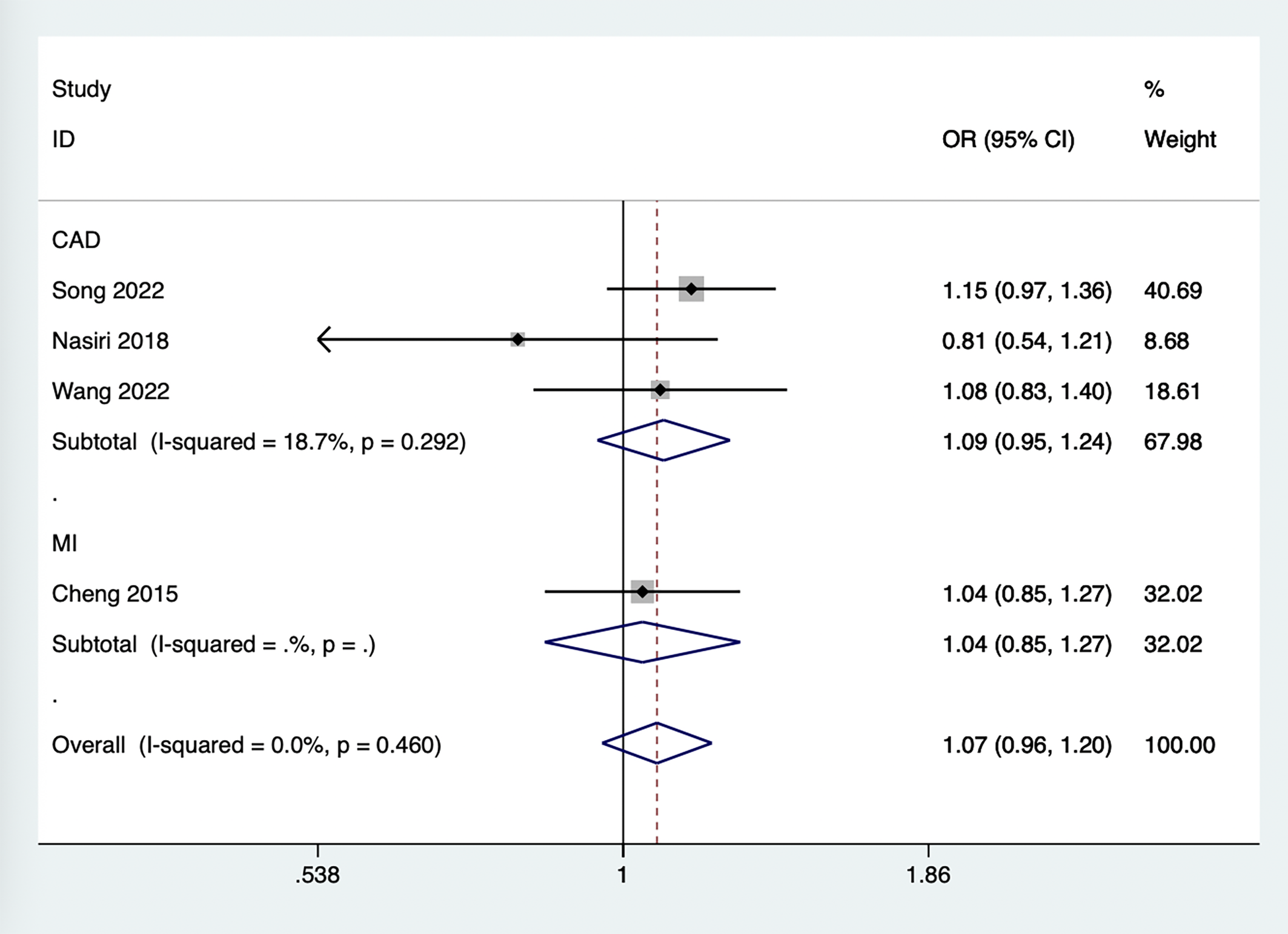


**Population subgroup analysis for rs4746720 under the allelic model (T vs C).**


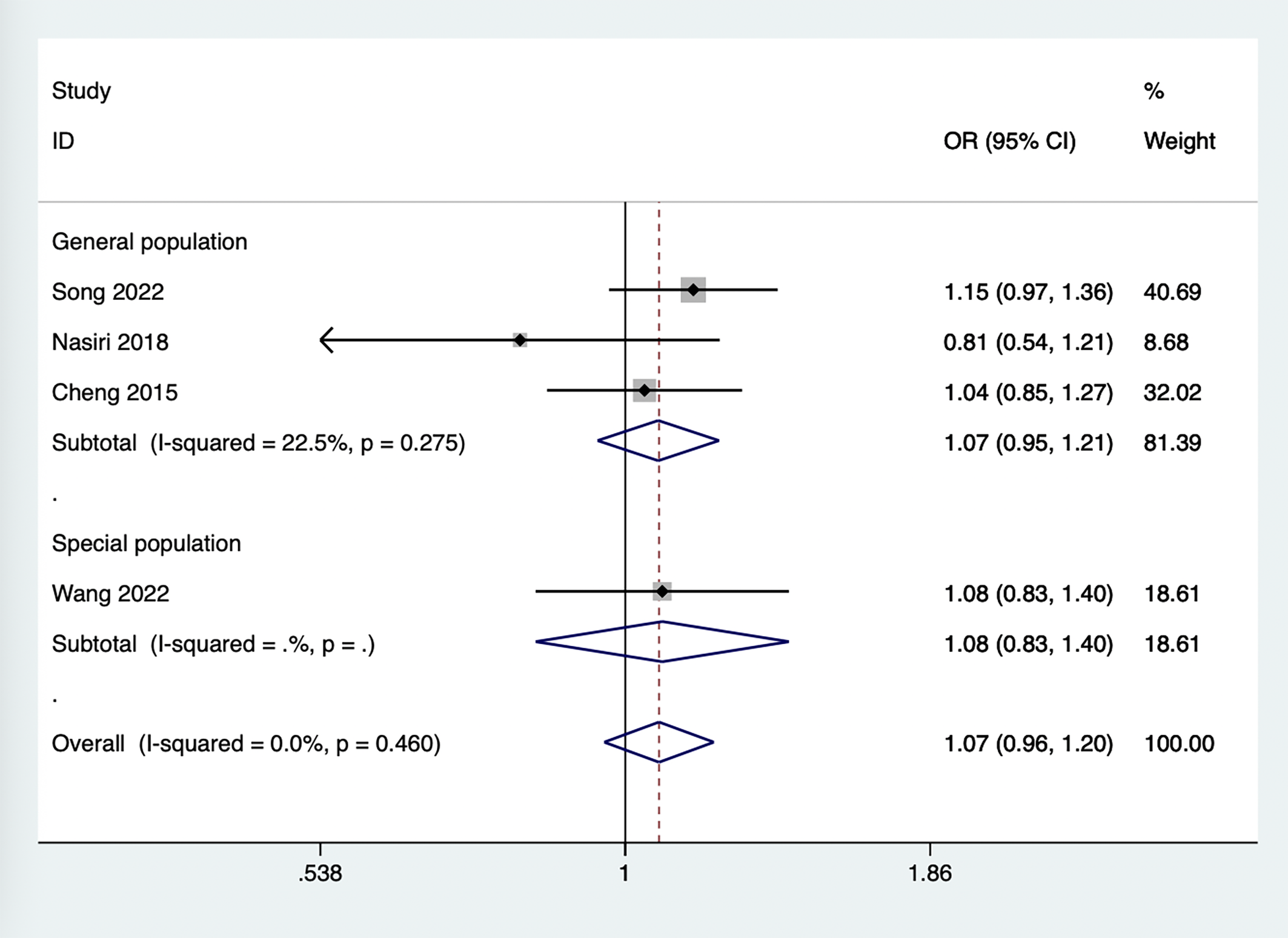


**Dominant model (TT+CT vs CC)**

**Overall meta-analysis for rs4746720 under the dominant model (TT+CT vs CC).**


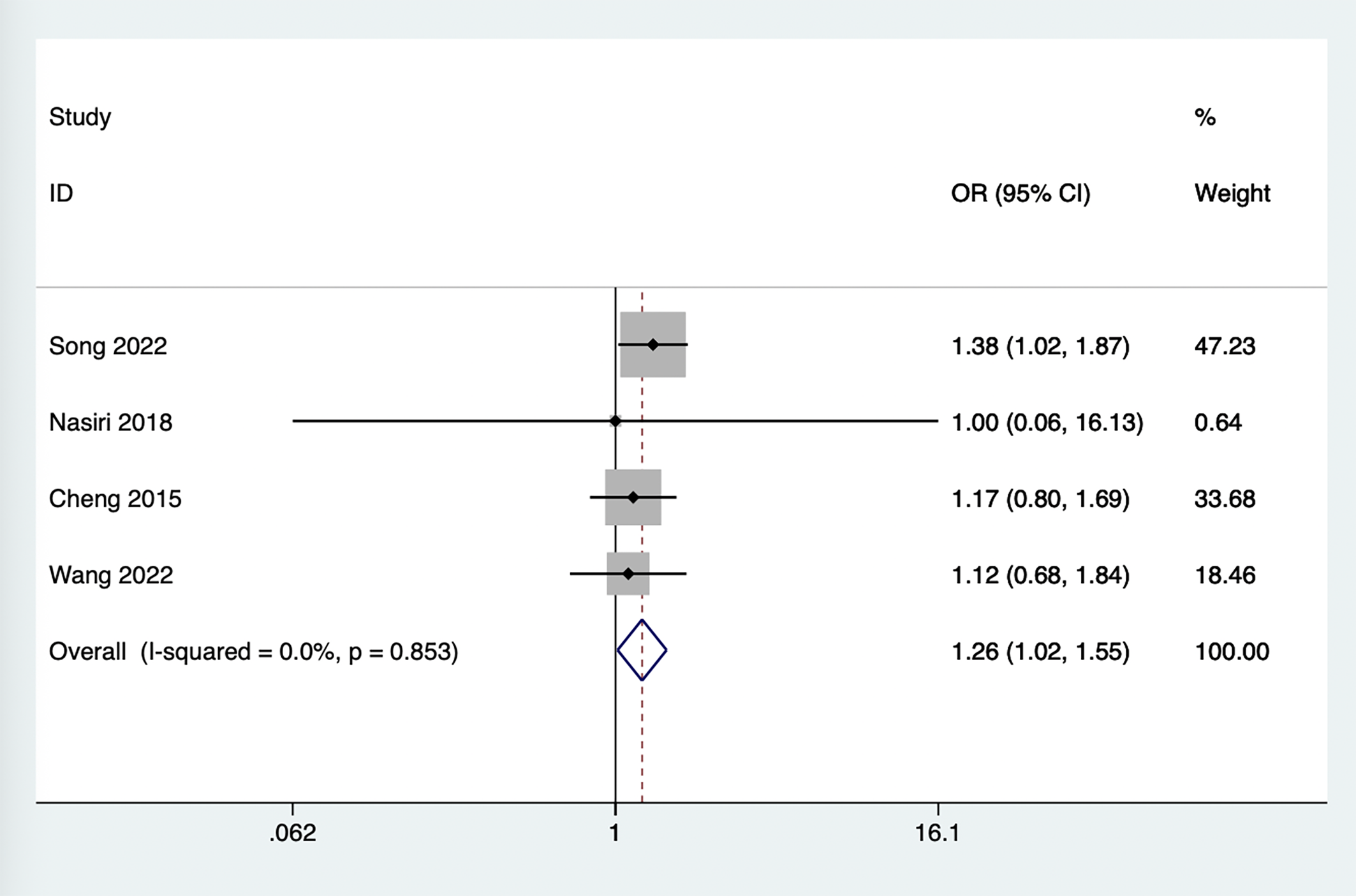


**Disease subgroup analysis for rs4746720 under the dominant model (TT+CT vs CC).**


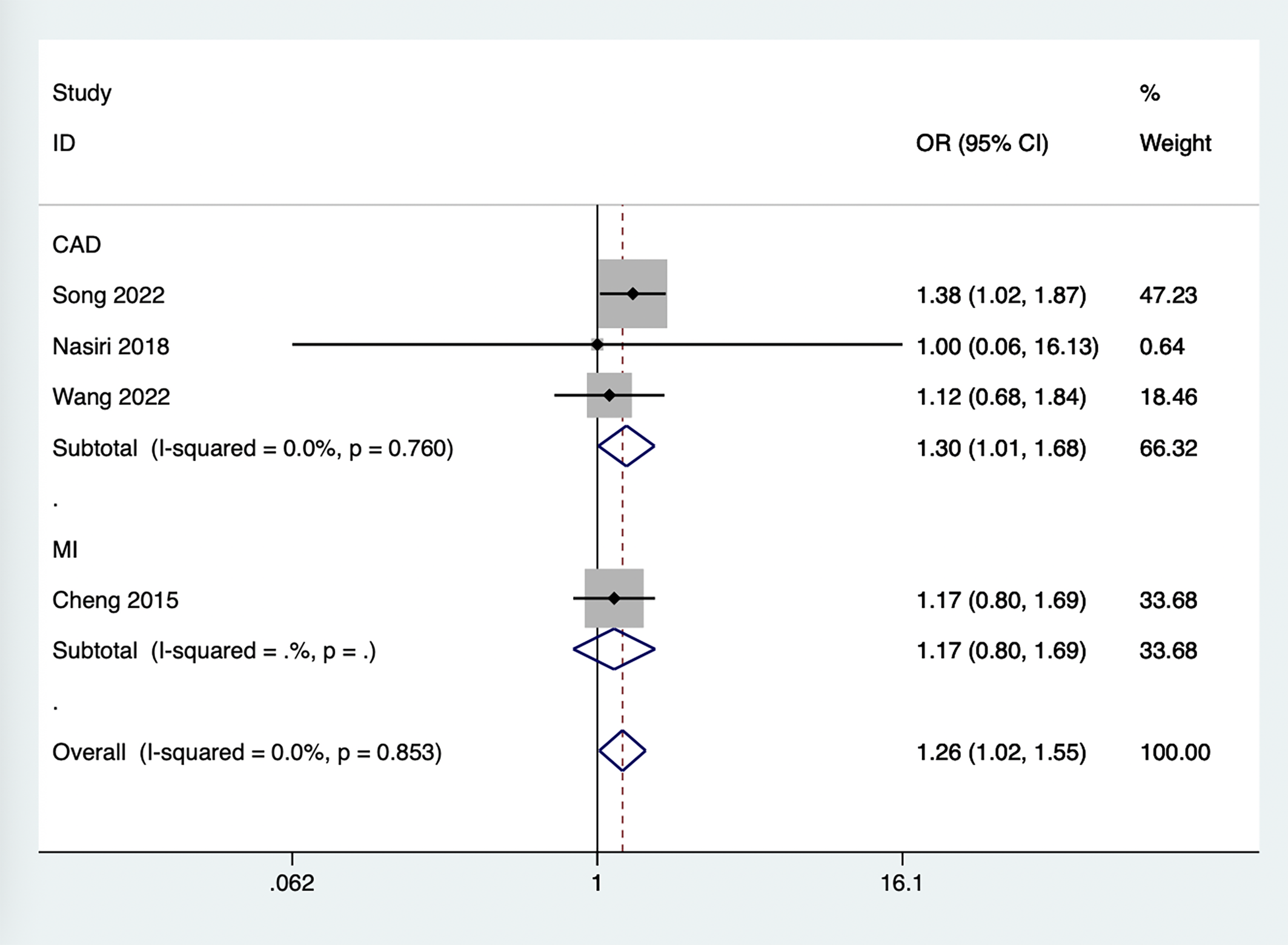


**Population subgroup analysis for rs4746720 under the dominant model (TT+CT vs CC).**


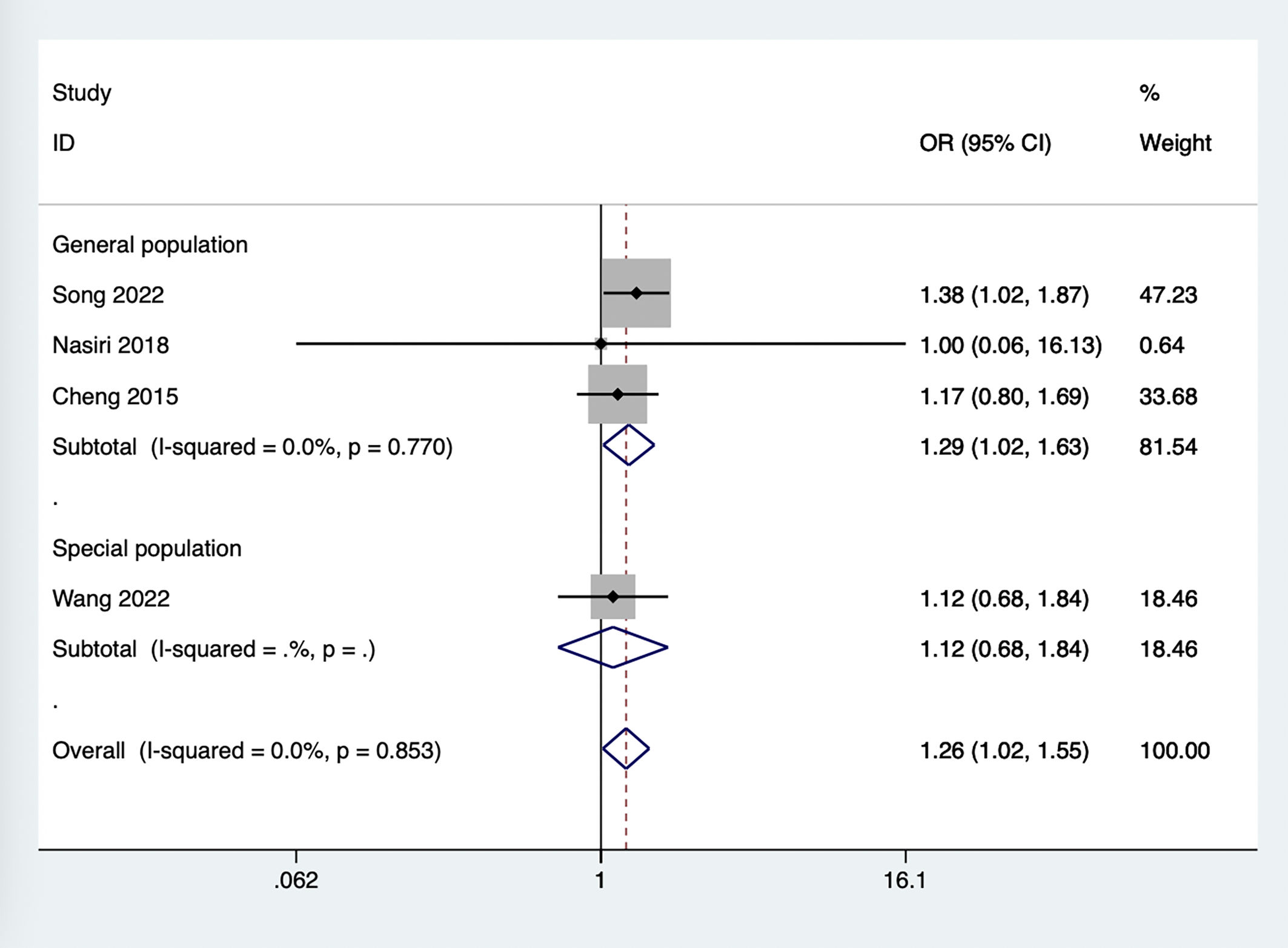


**Recessive model (TT vs CT+CC)**

**Overall meta-analysis for rs4746720 under the recessive model (TT vs CT+CC).**


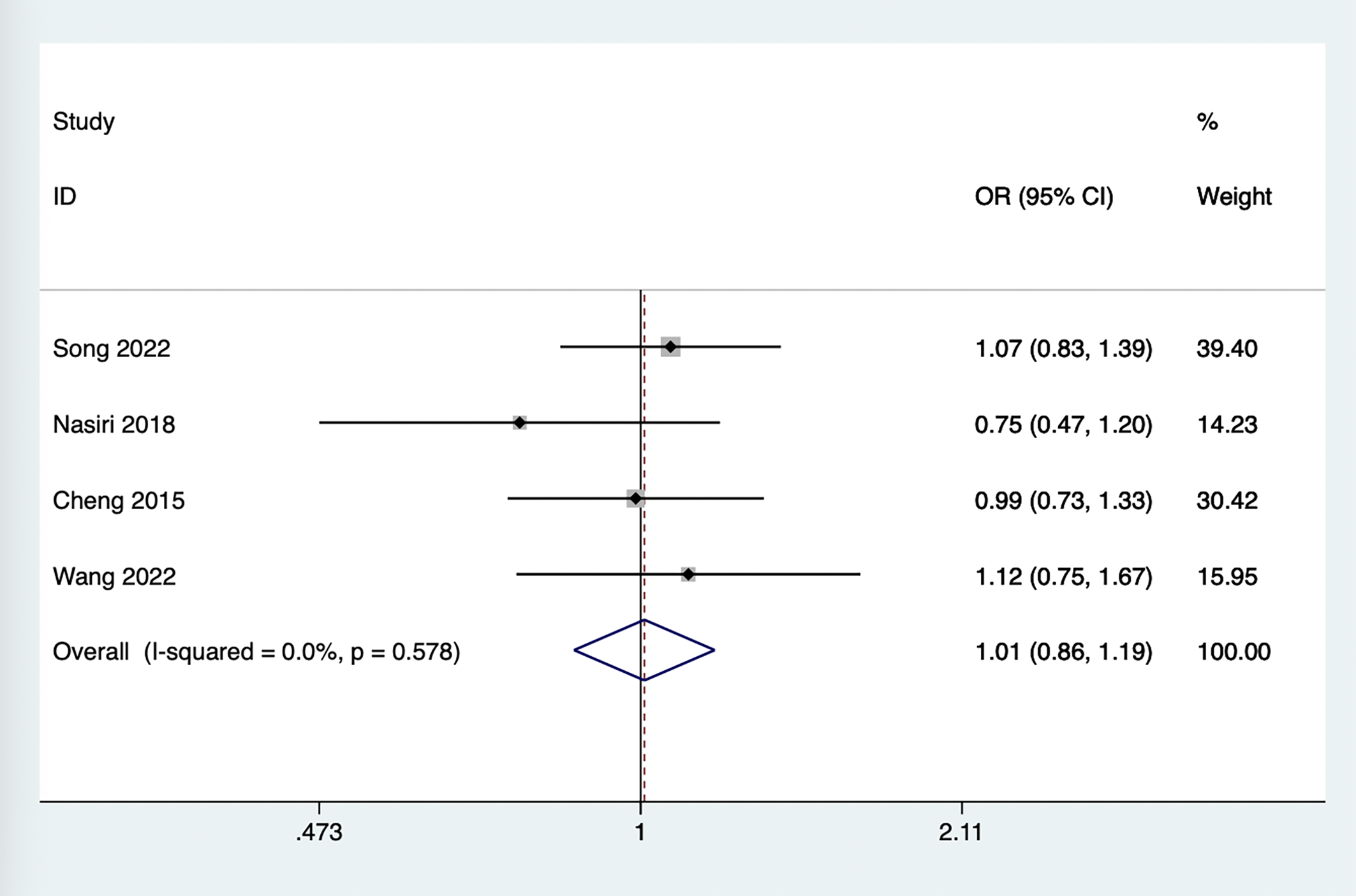


**Disease subgroup analysis for rs4746720 under the recessive model (TT vs CT+CC).**


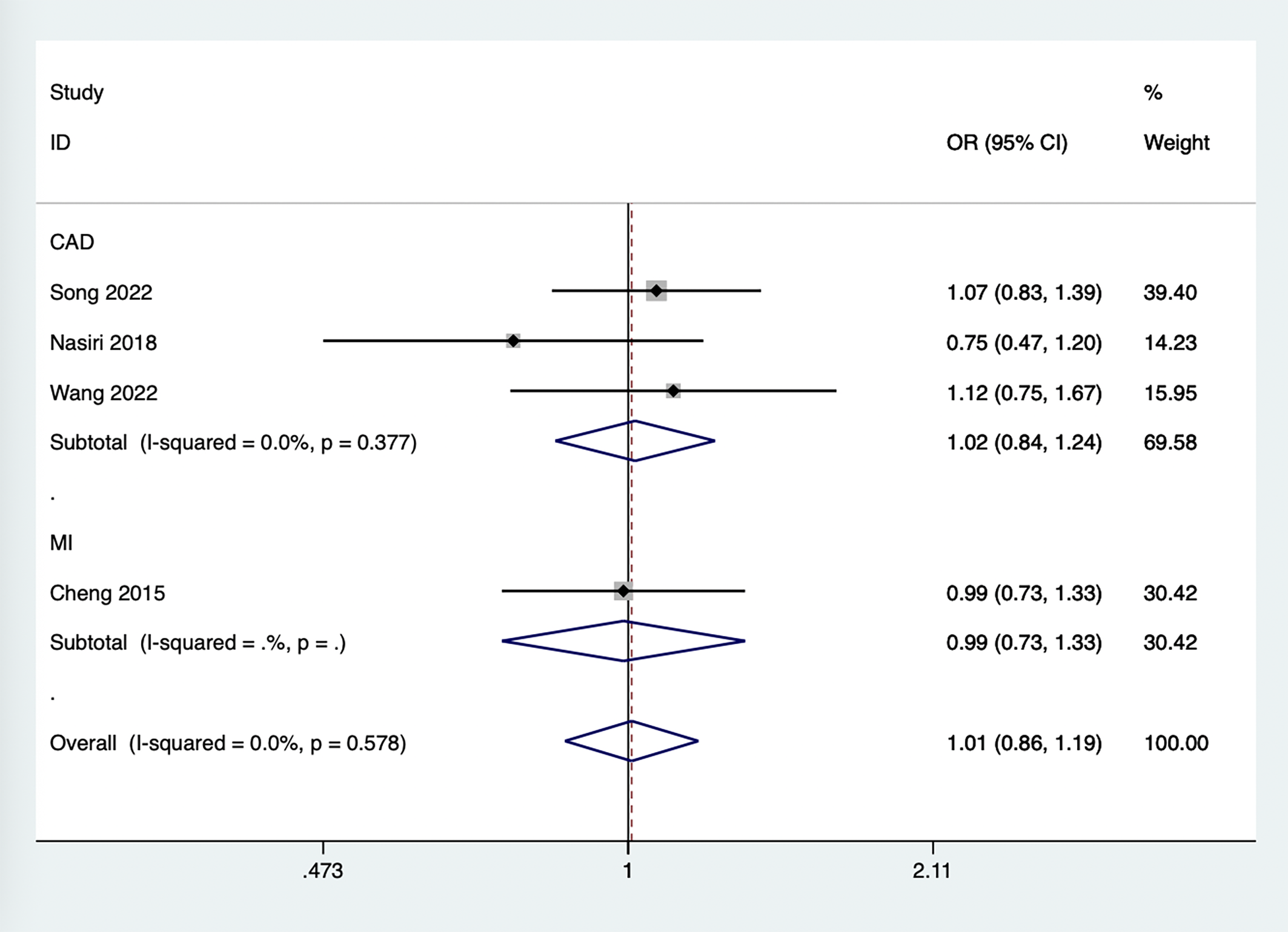


**Population subgroup analysis for rs4746720 under the recessive model (TT vs CT+CC).**


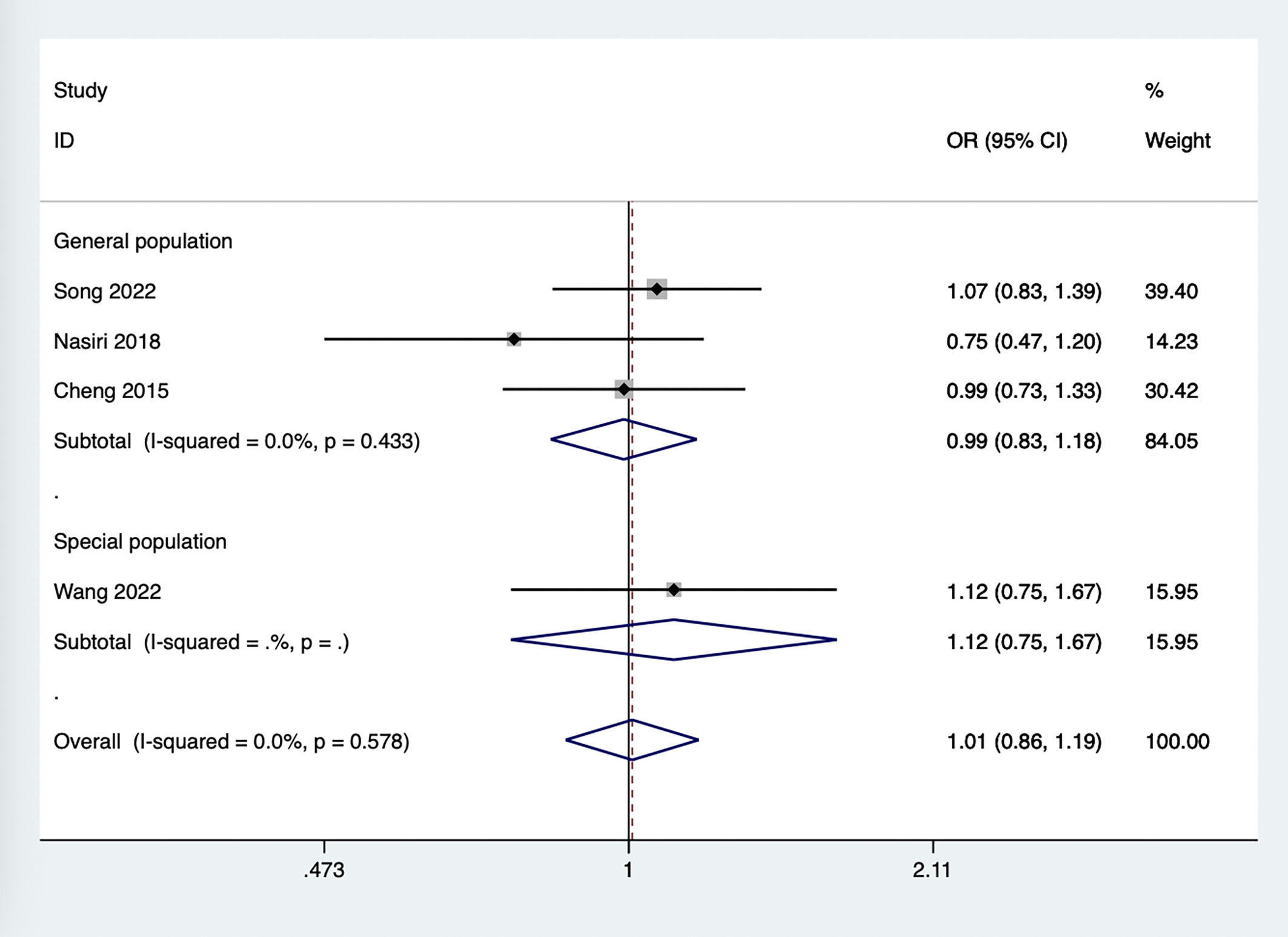


**Homozygote model (TT vs CC)**

**Overall meta-analysis for rs4746720 under the homozygote model (TT vs CC).**


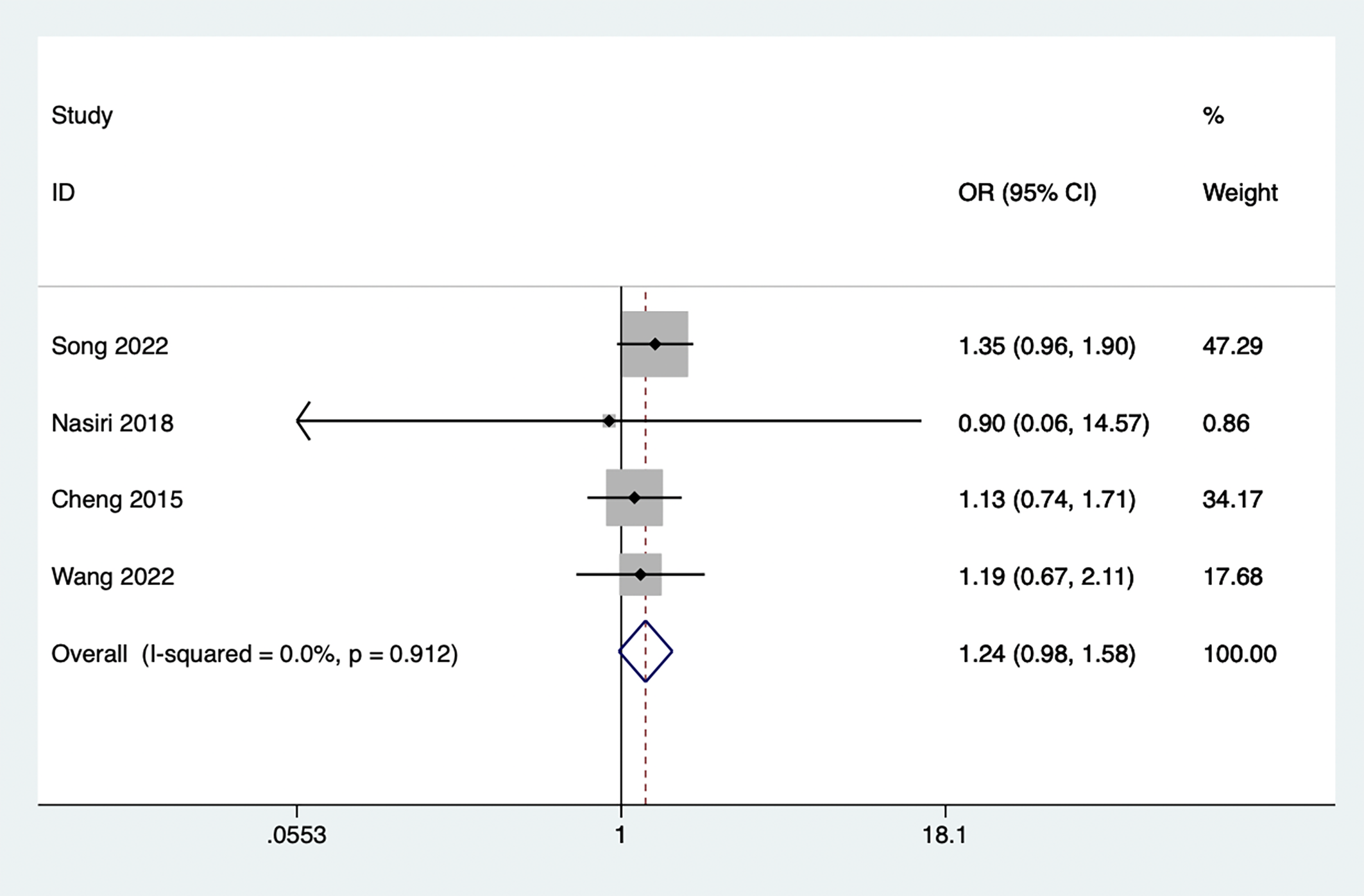


**Disease subgroup analysis for rs4746720 under the homozygote model (TT vs CC).**


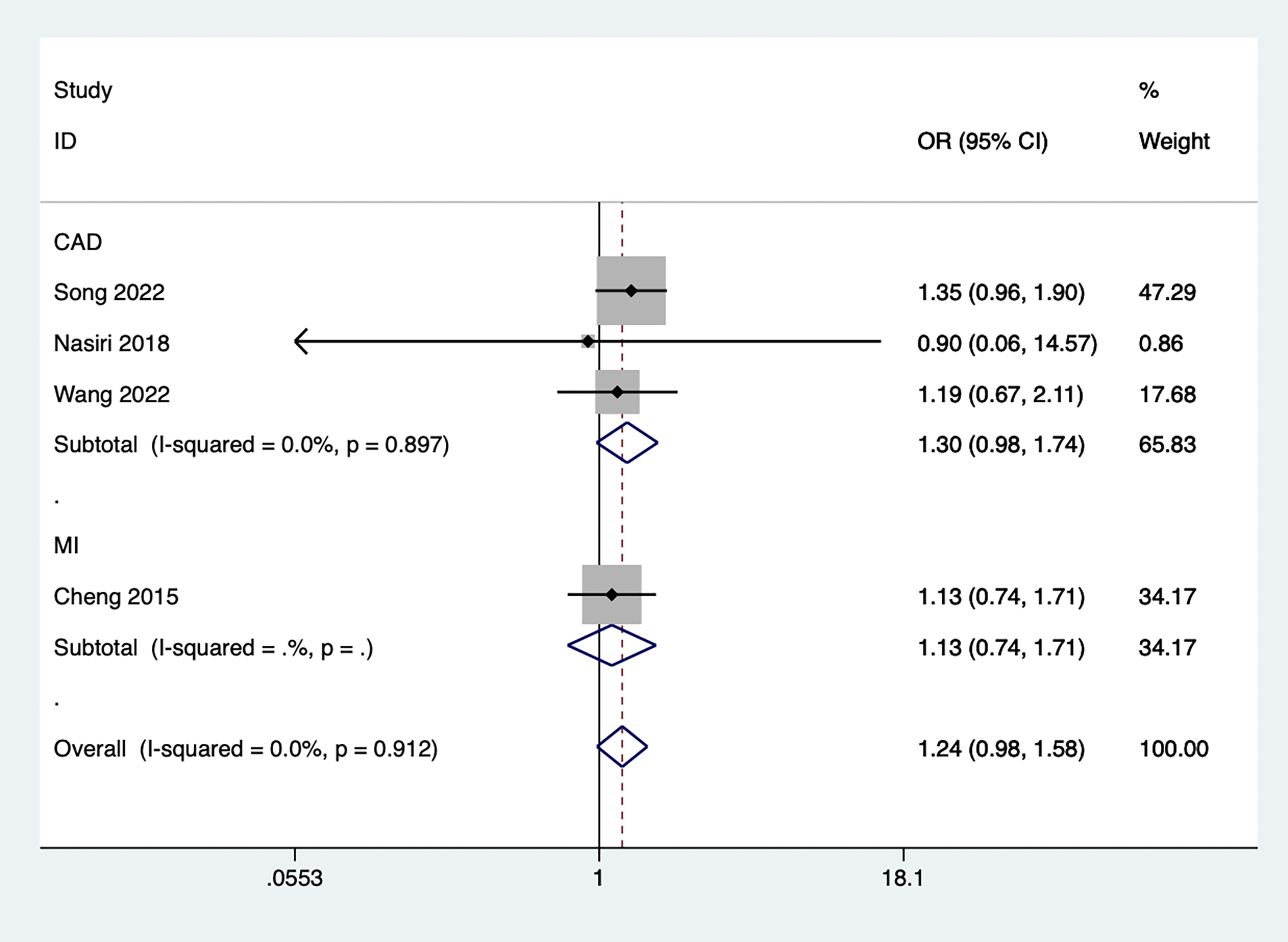


**Population subgroup analysis for rs4746720 under the homozygote model (TT vs CC).**


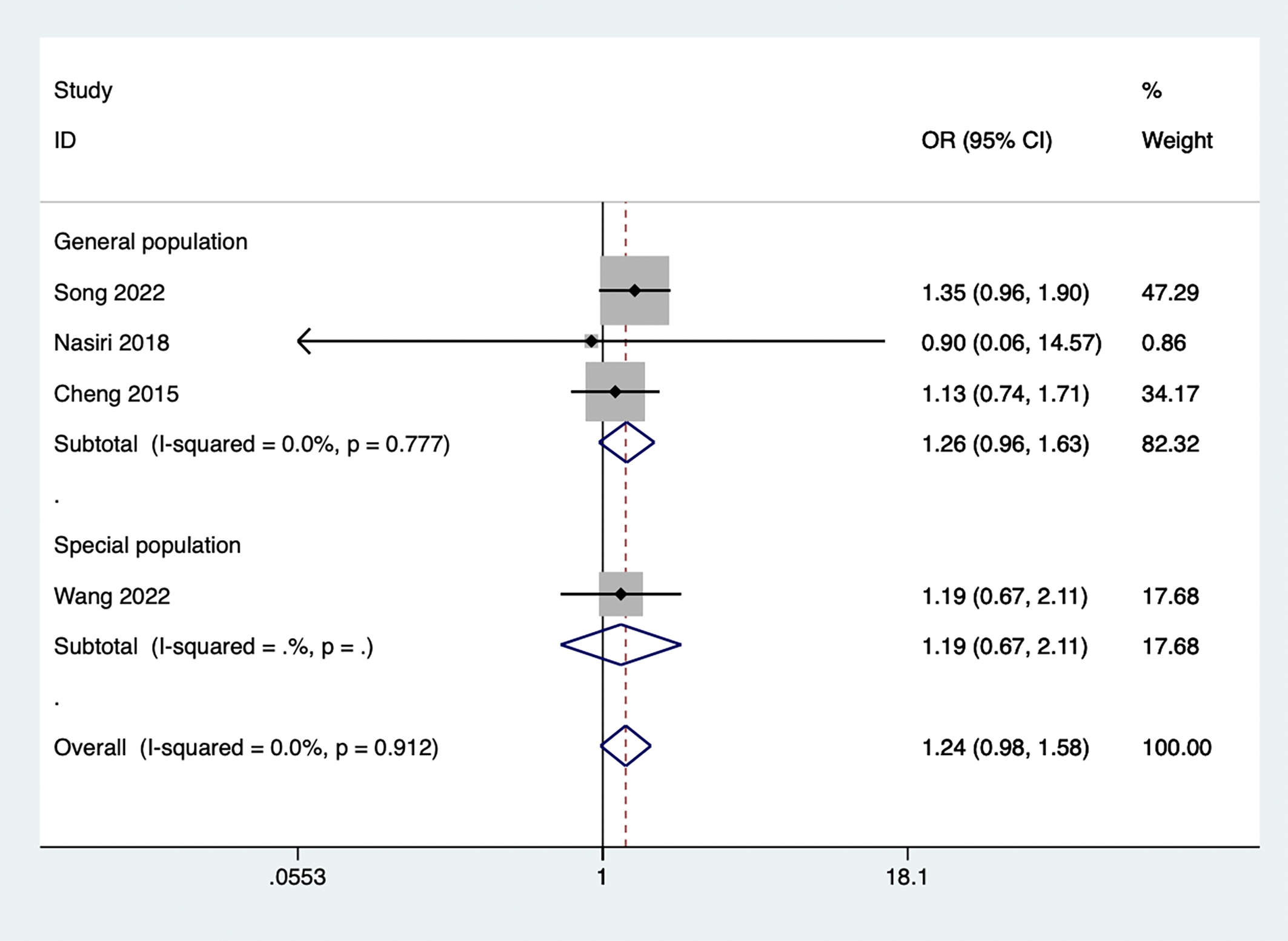


**Heterozygote model (CT vs CC)**

**Overall meta-analysis for rs4746720 under the heterozygote model (CT vs CC).**


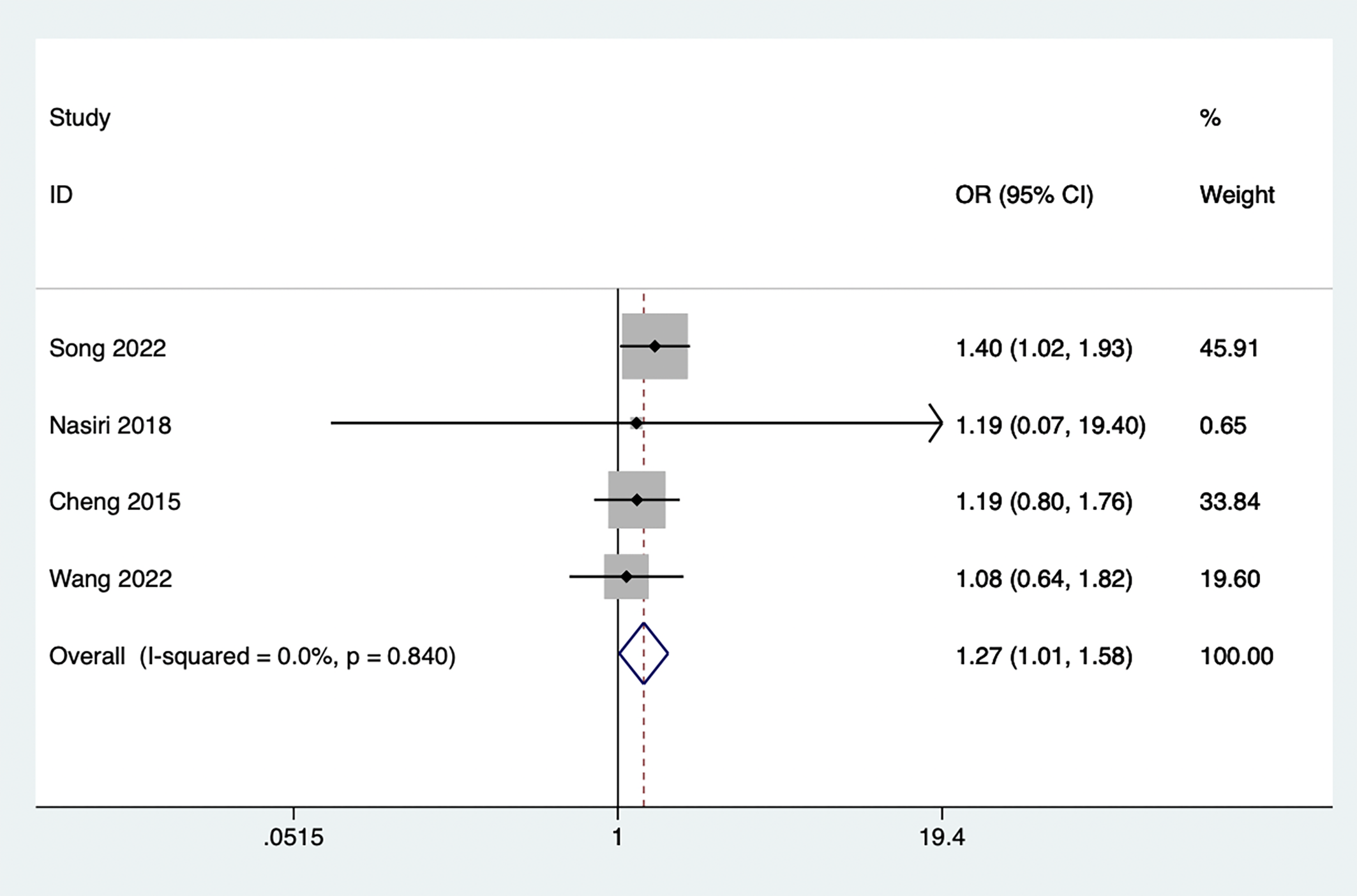


**Disease subgroup analysis for rs4746720 under the heterozygote model (CT vs CC).**


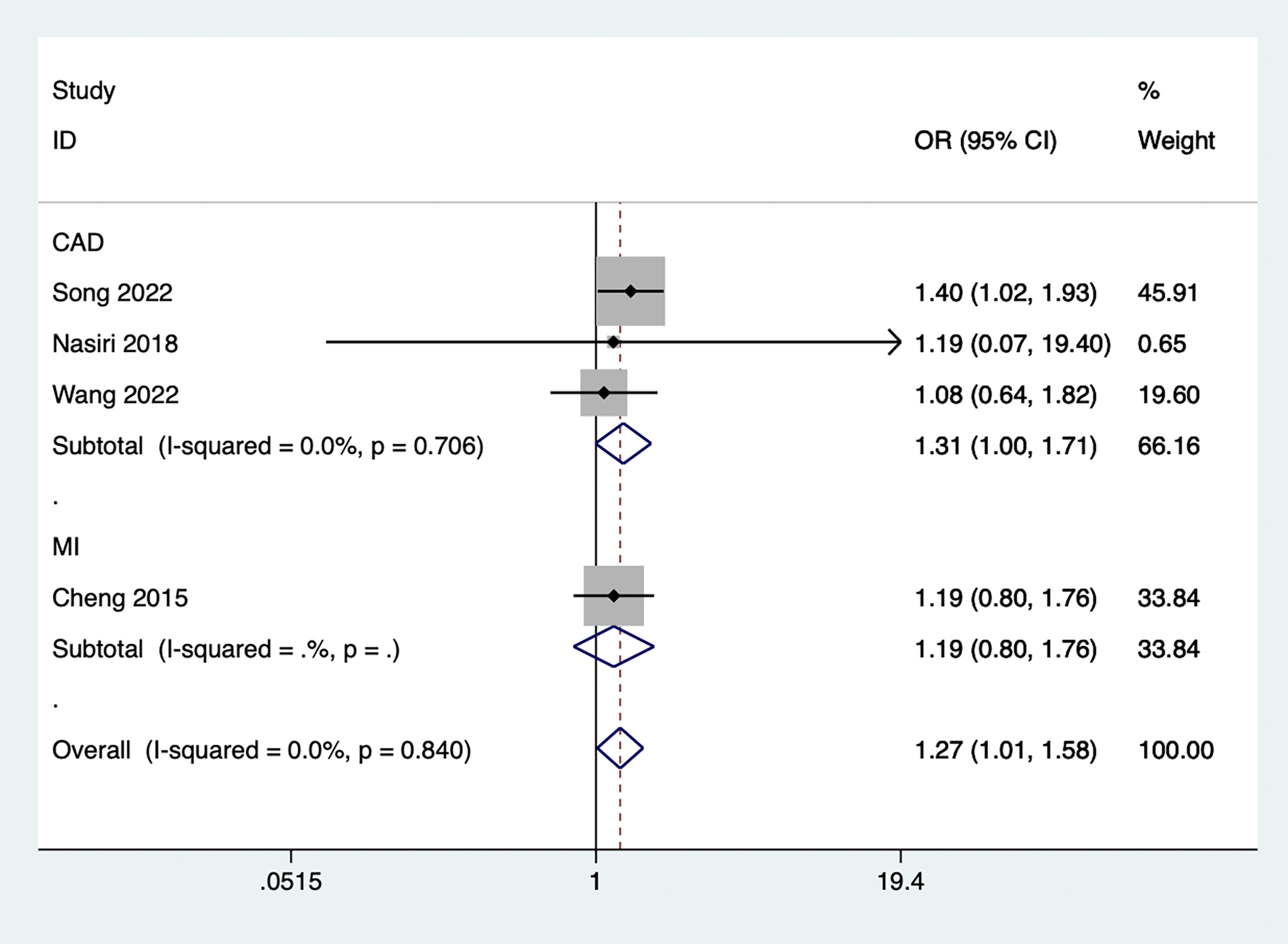


**Population subgroup analysis for rs4746720 under the heterozygote model (CT vs CC).**


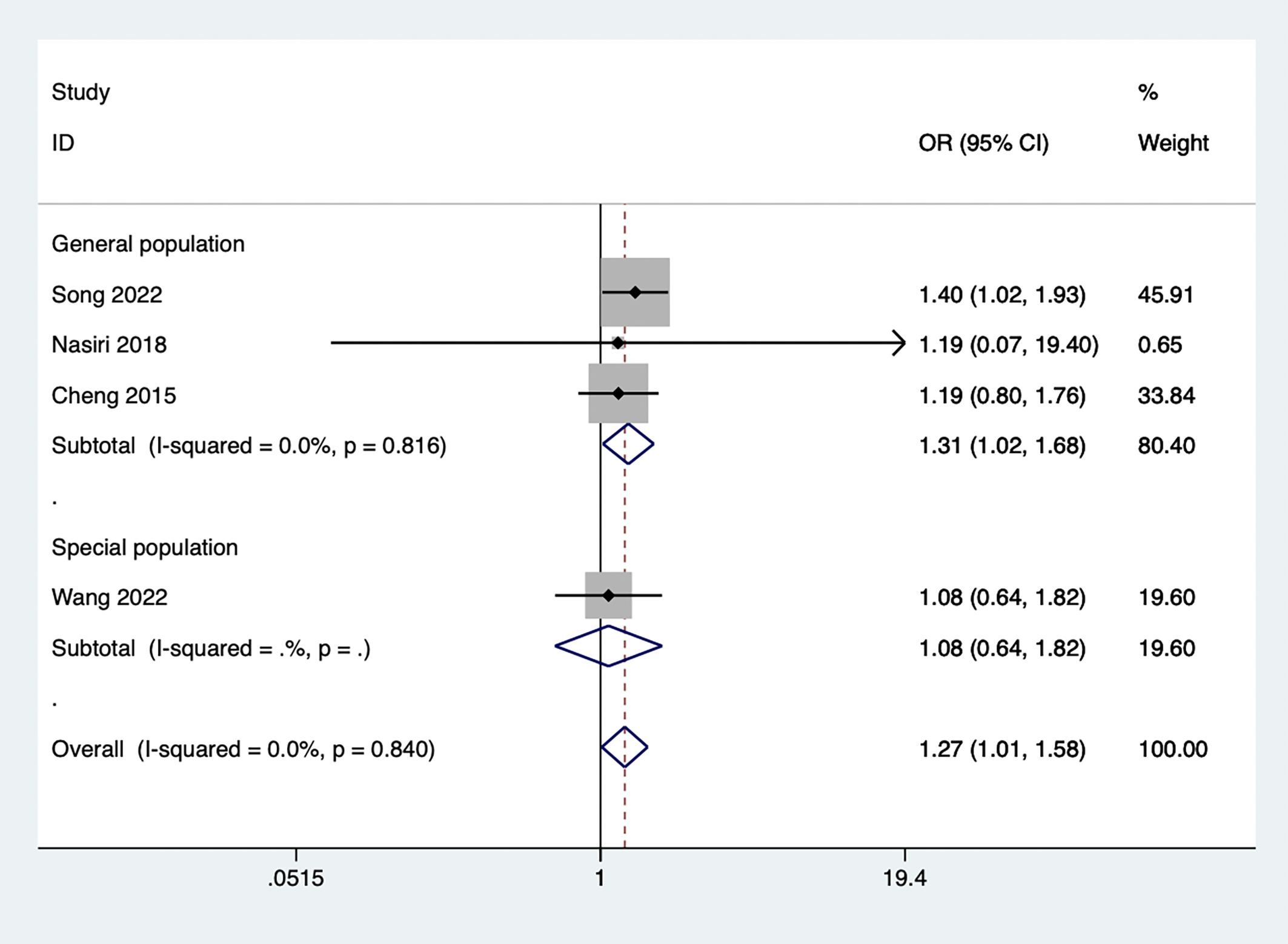

Supplement: Supplementary file 5 [file Table5.docx]
